# Quinoline-Based DNA Methyltransferase Inhibitors Featuring Basic Side Chains: Design, Synthesis, and Insight in Biochemical and Anticancer Cell Properties

**DOI:** 10.1021/acs.jmedchem.5c01029

**Published:** 2025-11-11

**Authors:** Clemens Zwergel, Chiara Lambona, Rossella Fioravanti, Alessia Raucci, Francesco Fiorentino, Guilaine Nchugoua Tchieh, Corinne Jallet, Laurent Lacroix, Michela Pierrettori, Francesca Romana Pellegrini, Yan Xiong, Jian Jin, Paola Arimondo, Daniela Trisciuoglio, Antonello Mai, Sergio Valente

**Affiliations:** † Department of Drug Chemistry and Technologies, 9311 Sapienza University of Rome, Piazzale Aldo Moro 5, Rome 00185, Italy; ‡ Université Paris Cité, CNRS UMR no 3523 Chem4Life, Epigenetic Chemical Biology, Department of Structural Biology and Chemistry, 27058 Institut Pasteur, F-75015 Paris, France; § IBENS, Département de biologie, École Normale Supérieure, Université PSL, CNRS, INSERM, Paris 75005, France; ∥ IBPM Institute of Molecular Biology and Pathology, 9327 CNR National Research Council of Italy, Via degli Apuli 4, Rome 00185, Italy; ⊥ Mount Sinai Center for Therapeutics Discovery, Departments of Pharmacological Science, Oncological Science and Neuroscience, Tisch Cancer Institute, 5925 Icahn School of Medicine at Mount Sinai, New York, New York 10029, United States

## Abstract

The quinoline DNMT inhibitors **4**–**21**, incorporating basic chains, were designed, synthesized, and evaluated for their ability to inhibit DNMT1 and DNMT3*A*/3L by directly measuring DNA methylation. Pharmacomodulation yielded nanomolar inhibitors with selectivity for either DNMT1 or DNMT3*A*/3L. The *meta*/*meta* analogs **7**–**14** exhibited the highest inhibition, with compounds **10** and **14** being the most potent and selective for DNMT3*A*/3L and DNMT1, respectively. DNA thermal denaturation experiments demonstrated for selected compounds strong DNA interaction. COBRA analysis in HCT-116 colon cancer cells revealed a selective reduction in P16^INK4A^ methylation, a tumor suppressor gene reactivated by DNMT inhibition. Among the tested cancer cell lines, HCT-116 was the most sensitive, and **14** showed the strongest antiproliferative effect. In isogenic HCT-116 P53^–/–^ cells, **14** exhibited reduced antiproliferative activity, lower apoptosis, and decreased levels of cleaved Caspase 3, P53, and γH2AX, confirming its P53-dependent mechanism of action linked to DNA damage.

## Graphical abstract

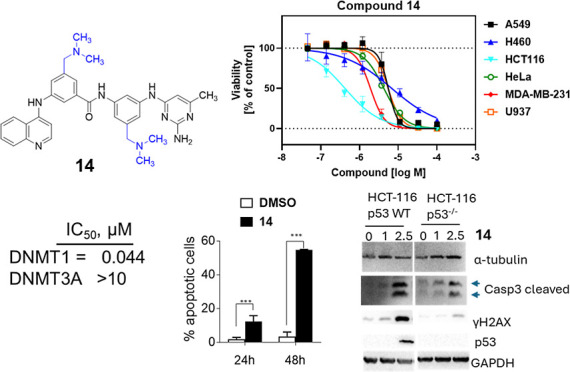

## Introduction

The onset and progression of cancer result from dysregulations linked to various interconnected factors, including lifestyle, environmental influences, and molecular mechanisms driven by genetic and/or epigenetic alterations.^1,2^ Epigenetic factors play a crucial role in the development of multiple human diseases, including cancer. These factors encompass aberrant expression or mutations in chromatin remodeling enzymes and alterations in the epigenome due to dysregulated writing, erasing, or reading activities of DNA- and histone-modifying proteins.^3–5^ DNA methylation is a key epigenetic mechanism that shapes chromatin structure and regulates gene expression, significantly impacting human genetics, development, and diseases.^4,6,7^ In mammals, this process predominantly occurs at the C5 position of cytosines within CpG dinucleotides and is mediated by a specialized group of 5-methylcytosine (5mC) methyltransferases known as DNA methyltransferases (DNMTs).^8^ In humans, three primary DNMT family members have been identified, playing essential roles in establishing (DNMT3A/B, *de novo* methyltransferases) or maintaining (DNMT1, maintenance methyltransferase) DNA methylation patterns in embryonic stem cells. These enzymes influence gene expression, genome reprogramming, organismal development, and cellular differentiation.^9–11^

Abnormal DNMT activity, including overexpression and/or mutations, has been implicated in a range of human diseases, including neurological,^12,13^ autoimmune,^14^ and metabolic disorders,^15^ as well as cancer.^16^ Specifically, tumor suppressor gene promoter hypermethylation leads to their silencing, while hypomethylation of proto-oncogenes results in their overexpression - both of which contribute to various solid and hematological malignancies.^17^ For instance, high expression levels of DNMT1 have been reported in some cancer cell lines including HCT-116 colon carcinoma, MDA-MB-231 triple negative breast cancer, and U937 histiocytic lymphoma.

Currently, azacytidine and decitabine are the only two nucleoside DNMT inhibitors (DNMTi) approved for clinical treatment of hematological malignancies. However, these agents suffer from chemical instability and severe side effects. Consequently, research efforts are increasingly focused on developing non-nucleoside DNMTi with distinct chemical structures and mechanisms of action.^18^

Among non-nucleoside DNMTi, SGI-1027 (**1**) is widely recognized and extensively used in various research contexts (https://pubmed.ncbi.nlm.nih.gov/?term=SGI-1027&sort=date). This molecule consists of four ring fragments (A–D, Figure 1) linked in a *para*/*para* orientation. Structural modifications, such as shifting the linkage position from *para* to *meta* or *ortho*, or duplicating the quinoline (fragment A) or pyrimidine (fragment D) moiety, led to a series of new regioisomers and analogs described by our research group in 2014.^19^ Among these derivatives, the *meta*/*meta* analog MC3343 (**2**) demonstrated greater potency and selectivity against DNMT1 and DNMT3A than **1**. In cancer cell panels, **2** exhibited slightly lower (U937 histiocytic lymphoma and MDA-MB-231 breast cancer) or comparable (RAJI Burkitt’s lymphoma and PC-3 prostate cancer) antiproliferative effects compared to **1**. Additionally, **2** was 2-fold less cytotoxic in peripheral blood mononuclear cells, used as a model for noncancerous healthy cells.^19^ In mouse medulloblastoma stem cells, **2** preferentially inhibited cell growth, while its *para*/*meta* regioisomer induced strong cell differentiation.^19^ In osteosarcoma (OS) and Ewing sarcoma (EWS), **2** effectively inhibited tumor proliferation in vitro (OS, EWS) and in vivo (OS). It also displayed synergistic effects when combined with doxorubicin and cisplatin (OS) or doxorubicin and talazoparib (EWS), a PARP inhibitor targeting DNA repair mechanisms.^20,21^ Further structural modifications of **2** led to derivatives with enhanced flexibility, achieved by introducing methylene units between different fragments or modifying the amide bond between fragments B and C. These compounds exhibited moderate activity in terms of DNMT inhibition and anticancer potential.^22^

**Figure 1:**
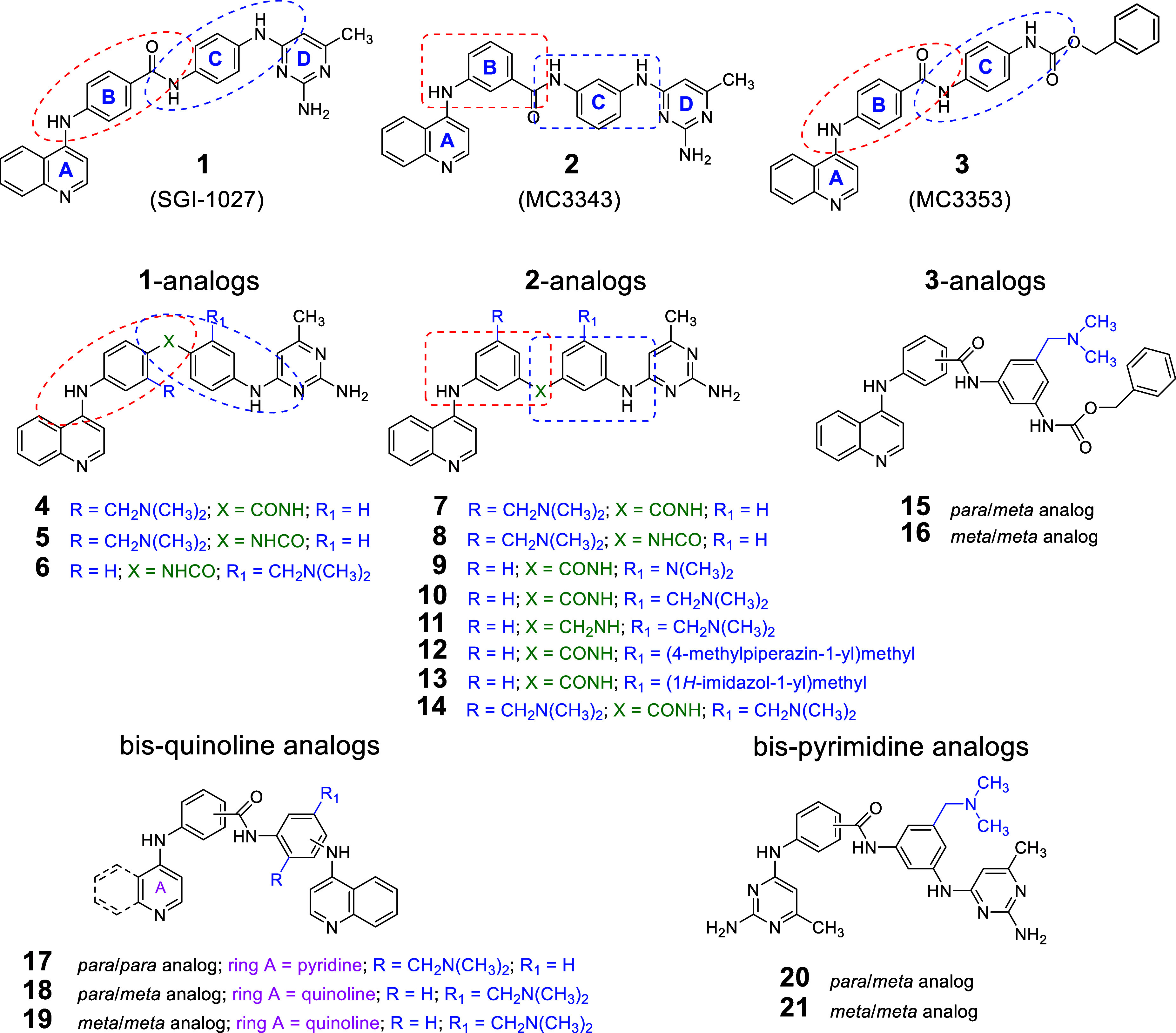
Structures of **1**–**3** and of the compounds **4**–**21** bearing basic side chain(s).

Lastly, replacing the 2,6-dimethylamino-4-methylpyrimidine moiety (fragment D) in **1** with a benzyl carbamate led to MC3353 (**3**, Figure 1). Although **3** exhibited weaker DNMT inhibitory activity than **1** and **2**, it demonstrates significant dose- and time-dependent antiproliferative activity in multiple cancer cell types, reaching submicromolar concentration.^23^ Notably, most of these compounds, including **1**–**3**, not only inhibited DNMT enzymatic activity but also led to DNMT protein downregulation in various cancer cell lines.^21–23^

Since some of these compounds exhibit low water solubility, we aimed to introduce a basic side chain into one of the phenyl rings of their structures. Among the synthesized compounds **4**–**21** (Figure 1), compounds **4**–**6** are analogs of **1** maintaining a *para*/*para* orientation between rings B and C, with a *N*,*N*-dimethylaminomethyl (CH_2_NMe_2_) side chain at either ring B (**4** and **5**) or ring C (**6**). Compound **5** differs from **4** in that it features an inverse amide function between the B/C rings instead of the carboxamide present in the **1** structure (Figure 1).

Compounds **7**–**14** are based on the **2** structure (*meta*/*meta* orientation of rings B and C), and show the insertion of a CH_2_NMe_2_ chain (with either the amide (**7**) or the inverse amide (**8**) function between B/C rings) at ring B. At ring C, a NMe_2_ (**9**), CH_2_NMe_2_ (**10**, with the amide linkage between the B/C rings, and **11**, with the methylamine at the same position), (*N*-methylpiperazin-1-yl)­methyl (**12**), or (1*H*-imidazole-1-yl)­methyl (**13**) basic side chain has been inserted. Compound **14** has two CH_2_NMe_2_ side chains, inserted at each of the B and C rings (Figure 1).

Compounds **15** and **16** are derived from the **3** structure, with **15** featuring a *para*/*meta* and **16** a *meta*/*meta* orientation between rings B and C, both incorporating a CH_2_NMe_2_ side chain at ring C (Figure 1).^22,23^

Additionally, the *bis*-quinoline and *bis*-pyrimidine templates^19,22^ were modified to introduce a CH_2_NMe_2_ side chain at ring C. Compounds **17**–**19** belong to the *bis*-quinoline group: in **17** the quinoline is simplified to pyridine linking fragments B and C in a *para*/*para* orientation, while **18** and **19** maintain the quinoline at ring A with a *para*/*meta* and *meta*/*meta* orientation at B and C rings, respectively. Finally, compounds **20** and **21** are *bis*-pyrimidine analogs incorporating the CH_2_NMe_2_ side chain at ring C, with **20** displaying a *para*/*meta* and **21** a *meta*/*meta* orientation (Figure 1).

In a previous study,^24^ we reported that six of these compounds (**7**, **9**, **10**, **12–14**) were able to inhibit DNMT1 at a low micromolar level. Some also exhibited activity against other DNA-interacting enzymes, such as base excision repair glycosylases and DNA/RNA polymerases. Compound **12** was found to induce a DNA damage response via p53 activation in A549 human nonsmall cell lung carcinoma (NSCLC) cells.

Here, we provide in full detail the synthetic procedures used to obtain all the cited compounds **4**–**21**, introducing **11**, **15**, and **17** for the first time. Their ability to induce hypomethylation in a cellular context was evaluated using a stable cellular reporter system (CMV-luc) in KG-1 human leukemia cells, in which luciferase expression is controlled by a methylated cytomegalovirus (CMV) promoter.^25^ Selected compounds were further assessed for their ability to demethylate p16^INK4A^, a tumor suppressor gene that can be reactivated by DNMT inhibition or deletion,^26^ in a solid cancer model, the HCT-116 colon carcinoma cell line, using the Combined Bisulfite Restriction Analysis (COBRA) assay.^27^ Thermal denaturation experiments were also conducted to evaluate the DNA-binding capabilities of selected derivatives, based on the detection of changes in DNA absorbance.^28^

Additionally, selected compounds were tested in a panel of cancer cell lines, including A549 and H460 (nonsmall cell lung cancer), HCT-116 (colon cancer), HeLa (cervical cancer), MDA-MB-231 (triple-negative breast cancer), and U937 (histiocytic lymphoma), to determine their antiproliferative potential. HCT-116 was the most sensitive cell line, prompting further investigation of compounds **9** and **14** regarding their effects on cell cycle progression and apoptosis induction. To explore the role of p53 pathway activation in its anticancer activity, compound **14** - the most potent derivative - was examined in both wild-type p53 and isogenic p53^–/–^ HCT-116 cell lines.

## Results

### Chemistry

The synthetic procedures used to prepare compounds **4**–**21** are depicted in Schemes 1–4.

**Scheme 1:**
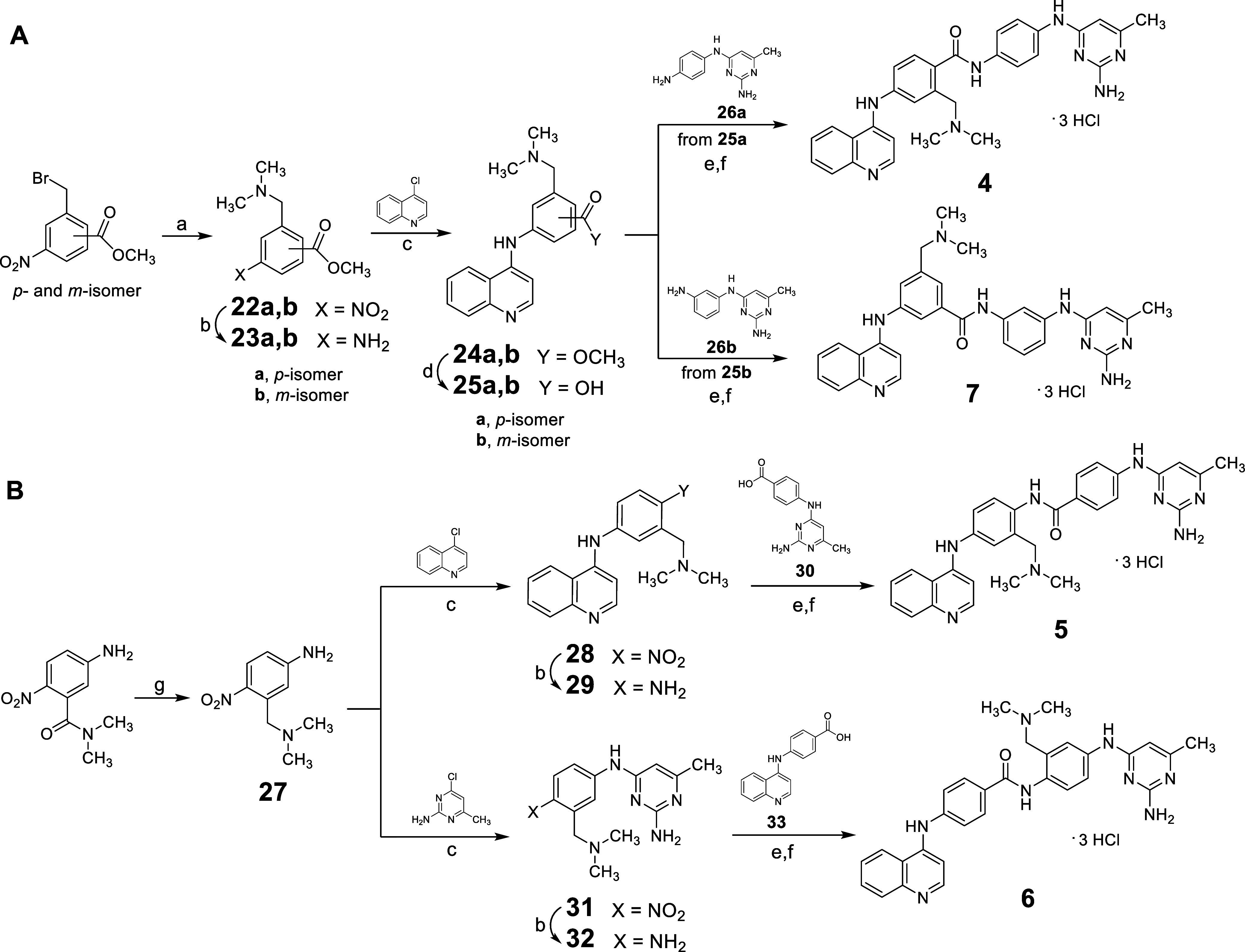
Synthesis of Compounds **4–7** ^a^

a (a) dimethylamine, dry THF, rt; (b) SnCl_2_.2H_2_O, 37% HCl, EtOH, rt, 1 h; (c) 37% HCl, EtOH, 75 °C, 1 h; (d) 2N KOH, EtOH, rt, overnight; (e) Et_3_N, PyBOP, dry DMF, N_2_, rt, 2.45 h; (f) 4 M HCl in dioxane, dry THF, 0 °C, 10 min; (g) BH_3_ in THF, THF, 70 °C, 6 h.

For the synthesis of **4** and **7**, commercially available methyl 2-(bromomethyl)-4-nitrobenzoate (for **4**) and methyl 3-(bromomethyl)-5-nitrobenzoate (for **7**) were treated with dimethylamine in THF to yield the nitro intermediates **22a**,**b**. These were subsequently reduced with stannous chloride to obtain the corresponding amines **23a**,**b**. Nucleophilic displacement of 4-chloroquinoline performed by **23a**,**b** in the presence of 37% hydrochloric acid (HCl) in ethanol at 75 °C afforded the methyl esters **24a**,**b**. Hydrolysis with 2N potassium hydroxide (KOH) yielded the corresponding carboxylic acids **25a**,**b**, which were then condensed with either *N* ^4^-(4-aminophenyl)- (**26a**)^19^ or *N* ^4^-(3-aminophenyl)- (**26b**)^19^ 6-methylpyrimidine-2,4-diamine in the presence of benzotriazol-1-yloxytripyrrolidinophosphonium hexafluorophosphate (PyBOP) and triethylamine in dry DMF. Final treatment with 4 M HCl in dioxane furnished the desired compounds **4** and **7** as hydrochlorides (Scheme 1A).

For the synthesis of **5** and **6**, the key intermediate **27** was obtained from commercially available 5-amino-*N*,*N*-dimethyl-2-nitrobenzamide via reduction with borane (BH_3_) in THF at 70 °C. Compound **27** was reacted with 4-chloroquinoline in the presence of 37% HCl in ethanol at 75 °C, yielding the nitro derivative **28,** which was subsequently reduced to the corresponding amine **29** using stannous chloride and 37% HCl in ethanol. Further condensation of **29** with 4-((2-amino-6-methylpyrimidin-4-yl)­amino)­benzoic acid **30** ^19^ in the presence of PyBOP and triethylamine in dry DMF afforded the reverse amide **5**, which was isolated as a hydrochloride following treatment with 4 M HCl. Alternatively, **27** was reacted with commercially available 2-amino-4-chloro-6-methylpyrimidine in the presence of 37% HCl in ethanol at 75 °C to generate the nitro intermediate **31**. Reduction with stannous chloride and 37% HCl in ethanol yielded amine **32**, which was then condensed with 4-(quinolin-4-ylamino)­benzoic acid **33** ^19^ in the presence of PyBOP and triethylamine in dry DMF. Final treatment with 4 M HCl in dioxane produced compound **6** as hydrochloride (Scheme 1B).

For the synthesis of compounds **8**–**14**, the key intermediates **36a**-**c** were prepared via an Appel reaction applied on commercially available di-*tert*-butyl (5-(hydroxymethyl)-1,3-phenylene) dicarbamate using tetrabromomethane and triphenylphosphine in dry THF. The resulting bromo derivative **34** was then treated with dimethylamine, *N*-methylpiperazine, or imidazole in dry THF to yield the protected amines **35a**-**c**, which were subsequently deprotected with 4 M HCl in dioxane to afford **36a**-**c**. Subsequent reaction of **36a** with the 3-((2-amino-6-methylpyrimidin-4-yl)­amino)­benzoic acid **37** ^19^ in the presence of triethylamine and PyBOP afforded the intermediate **38**, which was then treated with 4-chloroquinoline in an acidic medium to produce compound **8** as a hydrochloride. The reaction of **36a**-**c** with 3-(quinolin-4-ylamino)­benzoic acid **39**,^19^ triethylamine, and PyBOP yielded the intermediates **40a**-**c**, which were subsequently converted into **10**, **12**, and **13** as hydrochlorides via reaction with 2-amino-4-chloro-6-methylpyrimidine in an acidic medium. The reaction of **36a** with intermediate **25b** (see Scheme 1A) afforded intermediate **41**, which, upon treatment with 2-amino-4-chloro-6-methylpyrimidine, yielded the *bis*-dimethylaminomethyl derivative **14** (Scheme 2A).

**Scheme 2:**
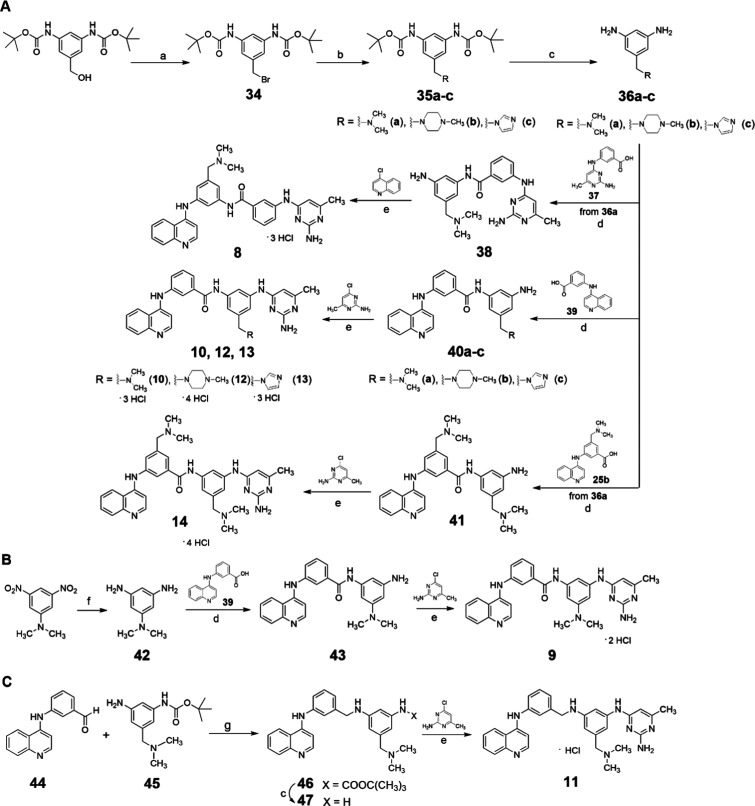
Synthesis of Compounds **8–13** ^a^

a (a) CBr_4_, Ph_3_P, N_2_, rt, 3 h; (b) appropriate amine, dry THF, rt; (c) 4 M HCl in dioxane, dry THF, 0 °C to rt; (d) Et_3_N, PyBOP, dry DMF, N_2_, rt, 2.45 h; (e) 37% HCl, EtOH, 75 °C, 1 h; (f) SnCl_2_·2H_2_O, 37% HCl, EtOH, rt, 1 h; (g) NaBH­(OAc)_3_, dry DCE, 60 °C, overnight.

For the synthesis of compound **9**, commercially available *N*,*N*-dimethyl-3,5-dinitroaniline was reduced with stannous chloride to yield the corresponding diamine **42**, which was reacted with **39** ^19^ to afford intermediate **43**. Subsequent treatment of **43** with 2-amino-4-chloro-6-methylpyrimidine furnished compound **9** (Scheme 2B).

Compound **11** was synthesized by reacting 3-(quinolin-4-ylamino)­benzaldehyde **44** ^22^ with *tert*-butyl (3-amino-5-((dimethylamino)­methyl)­phenyl)­carbamate **45** - obtained by halting the formation of the free phenylendiamine **36a** before completion (see Experimental Section) - in the presence of sodium acetoxyborohydride in dry dichloroethane. The resulting intermediate **46** was immediately deprotected with 4 M HCl to yield **47**, which was then treated with 2-amino-4-chloro-6-methylpyrimidine to afford compound **11** (Scheme 2C).

Treatment of the key intermediate **36a** with benzyl chloroformate and triethylamine in dry DCM furnished the benzyl (3-amino-5-((dimethylamino)­methyl)­phenyl)­carbamate **48**, which gave **15** or **16** after reaction with **33** ^19^ or **39**,^19^ respectively, according to the reported procedure (Scheme 3).

**Scheme 3:**
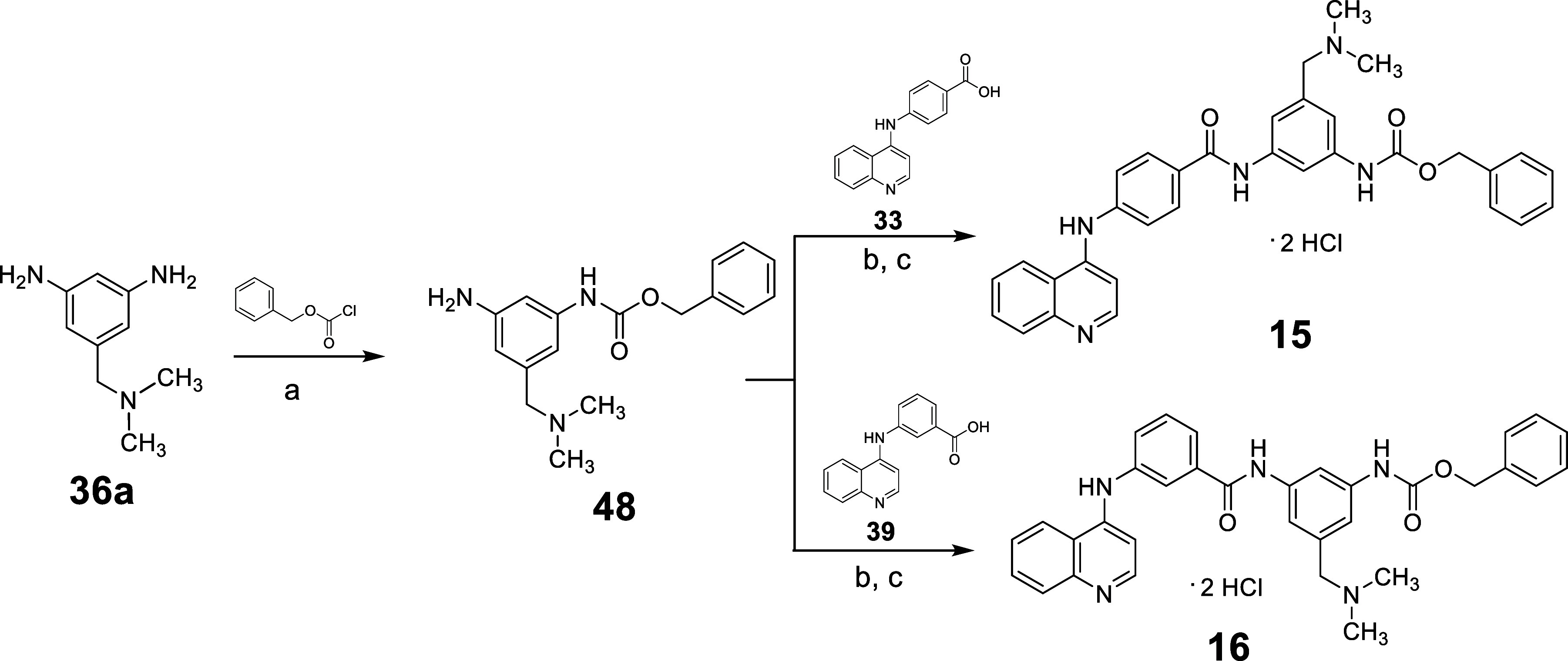
Synthesis of Compounds **15** and **16** ^a^

a (a) Et_3_N, dry DCM, rt, overnight; (b) Et_3_N, PyBOP, dry DMF, N_2_, rt, 2.45 h; (c) 4 M HCl in dioxane, dry THF, 0 °C, 10 min.

The synthesis of compound **17** was achieved by treating 4-(pyridin-4-ylamino)­benzoic acid **49** ^29^ with **29** in the presence of PyBOP and triethylamine in dry DMF, followed by treatment with 4 M HCl (Scheme 4A). For the synthesis of the *bis*-quinoline compounds **18** and **19**, 5-((dimethylamino)­methyl)­benzene-1,3-diamine **36a** was treated with 4-(quinolin-4-ylamino)­benzoic acid **33** ^19^ to yield intermediate **50**, which, upon treatment with 4-chloroquinoline, afforded the *para*/*meta* compound **18**. The *meta*/*meta* analog **19** was obtained through the reaction of **40a** and 4-chloroquinoline in 37% HCl and ethanol. Alternatively, the reaction of **36a** with ((2-amino-6-methylpyrimidin-4-yl)­amino)­benzoic acid **30** ^19^ produced intermediate **51**, which, upon treatment with 2-amino-4-chloro-6-methylpyrimidine, yielded the *para*/*meta bis*-pyrimidine analog **20**. Finally, treatment of intermediate **38** with 2-amino-4-chloro-6-methylpyrimidine furnished the *meta*/*meta* isomer **21** (Scheme 4B).

**Scheme 4:**
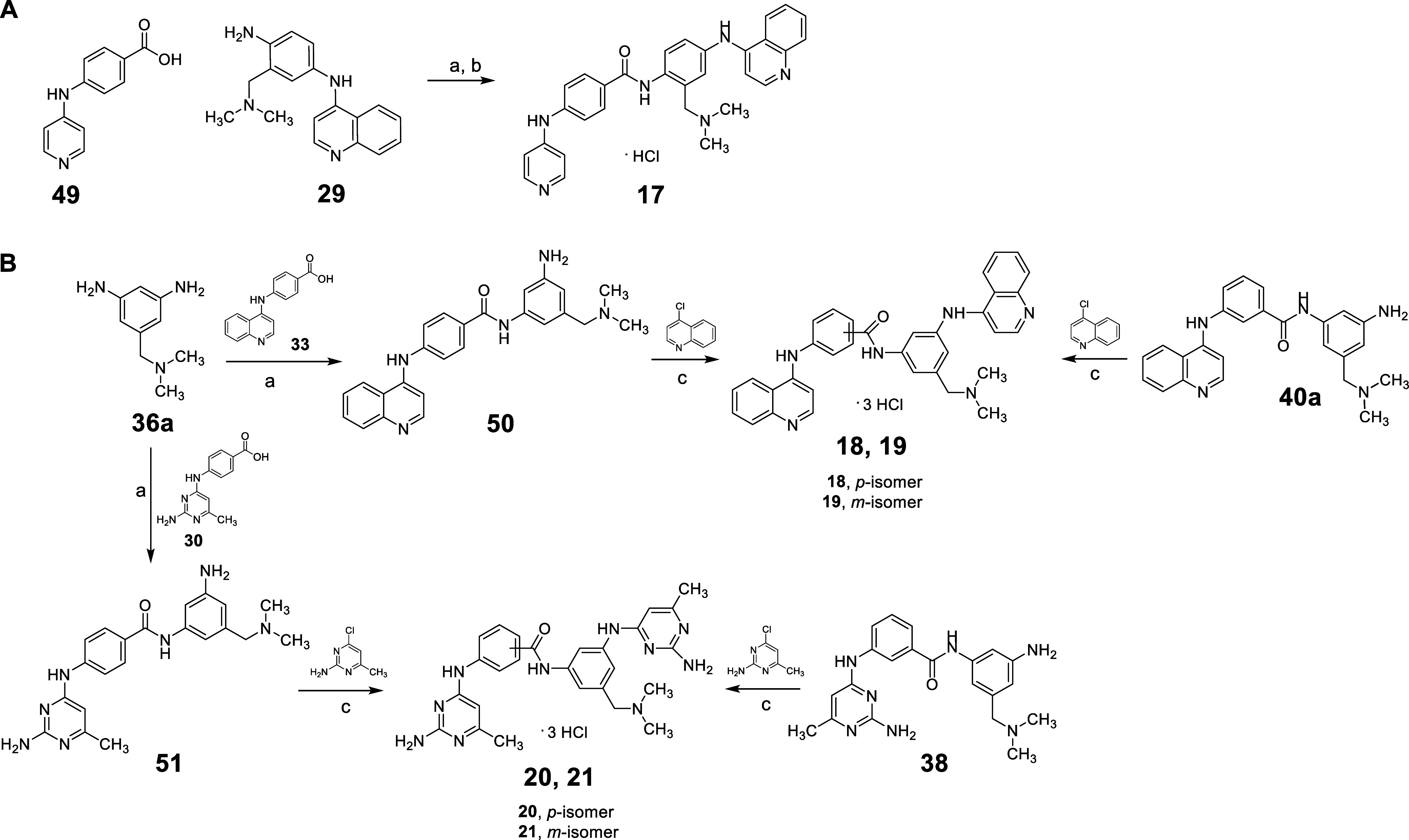
Synthesis of Compounds **17–21** ^a^

a (a) Et_3_N, PyBOP, dry DMF, N_2_, rt, 2.45 h; (b) 4 M HCl in dioxane, dry THF, 0 °C, 10 min; (c) 37% HCl, EtOH, 75 °C, 1 h.

Chemical and physical data for the final compounds **4**–**21** and the intermediates **22**–**25**, **28**–**32**, **34–36**, **38**, **40–51** are reported in the Experimental Section. Elemental analyses for **4**–**21** are reported in Supporting Information, Table S1. High-performance liquid chromatography (HPLC) traces for selected compounds **7**, **9**, **10**, **12**–**14** are reported in Supporting Information, Figures S1–S6.

### Human DNMT1 and DNMT3A Inhibition Were Assessed through Direct Measurement of the DNA Methylation on 40-mer DNA Duplexes

Recently, some of us reported the effects of 10 μM compounds **4–10**, **12–14**, **16**, and **18–21** against human DNMTs and other DNA-interacting enzymes, using an assay that measured the conversion of *S*-adenosyl-l-methionine (SAM) to *S*-adenosyl-l-homocysteine (SAH), which is subsequently transformed into ATP and detected via a luciferase reaction.^24^ In these assays, an [E]/[DNA] ratio of 1:10 ([DNA] = 5 μM) was used. Among the 15 tested derivatives, six compounds (**7**, **9**, **10**, **12–14**) completely abrogated DNMT1 activity at 10 μM, exhibiting IC_50_ values in the range of 1.9–3.5 μM. At the same concentration (10 μM), these compounds also reduced the activity of three adenine methyltransferases below 50%.^24^ Additionally, they inhibited glycosylases and polymerases.^24^ Against the full-length DNMT3A2–3L complex, these compounds were initially almost inactive. However, when tested at a different [E]/[DNA] ratio of 1:2 ([DNA] = 1 μM), all displayed >50% of inhibition at 10 μM.^24^

These findings prompted us to re-evaluate the effects of the entire set of compounds **4**–**21** (including **11**, **15**, and **17**) against human DNMT3A. For comparison, we also included compounds **1** and **2**, together with the 3-(quinolin-4-ylamino)-*N*-(3-(quinolin-4-ylamino)­phenyl)­benzamide [**2b**] previously reported by us as a DNMT3A-selective inhibitor.^22^ We employed an alternative method developed by some of us,^30^ which detects methylation at a single CpG site in a 40-mer DNA duplex using a restriction enzyme inhibited when the DNA is methylated. This method offers the advantage of using an optimal DNA substrate for human catalytic DNMT3*A*/3L (DNMT3Acat) and directly measuring the methylation event.

We further adapted the assay to evaluate the methylation activity of hDNMT1 on hemimethylated DNA. We optimized the DNA sequence and the restriction enzyme that, for this maintenance DNMT, needs to distinguish between hemimethylated DNA (the initial substrate) and methylated DNA (the product of the reaction). The assay was optimized using the reference inhibitors **1**,^31^ GSK3685032 ^32,33^ and compound #20 ^27^ (data not shown), and was subsequently applied to test compounds **4**–**21**. In addition to **4**–**21**, compounds **1**, **2**, and the DNMT1-selective inhibitors GSK-3484862 and GSK-3685032 were added.^32,33^

The IC_50_ values reported in Table 1 indicate that introducing a dimethylaminomethyl side chain at ring B of compound **1** resulted in a moderately active DNMT1/3A inhibitor (**4**), which was 9-fold less potent against DNMT3A and 2-fold more potent against DNMT1 than **1**. However, applying the same modification at ring C rendered the inactive compound **6**. Additionally, the inversion of the amide function linking the B/C rings in compound **4** (leading to compound **5**) significantly reduced its anti-DNMT activity.

**Table 1:**
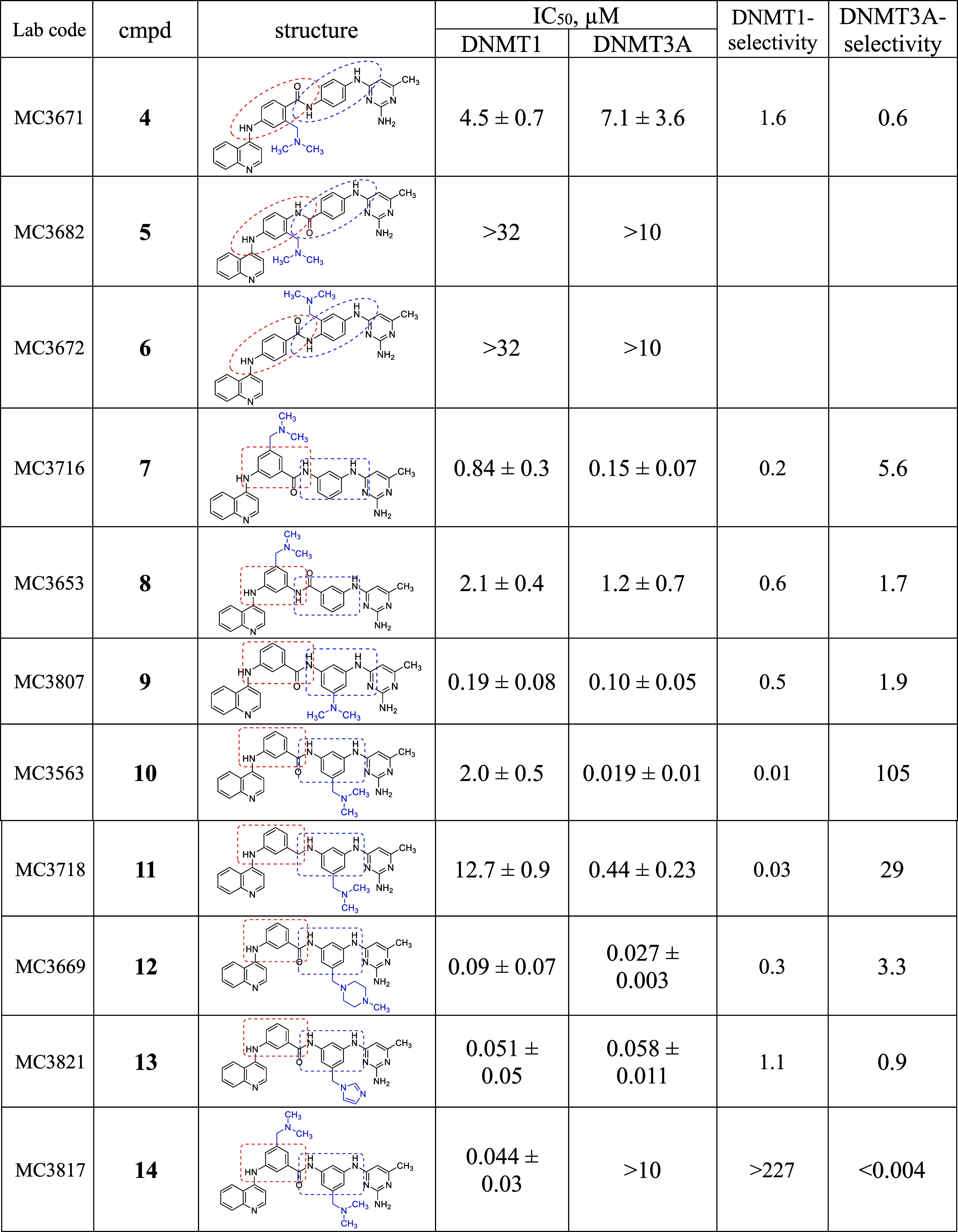

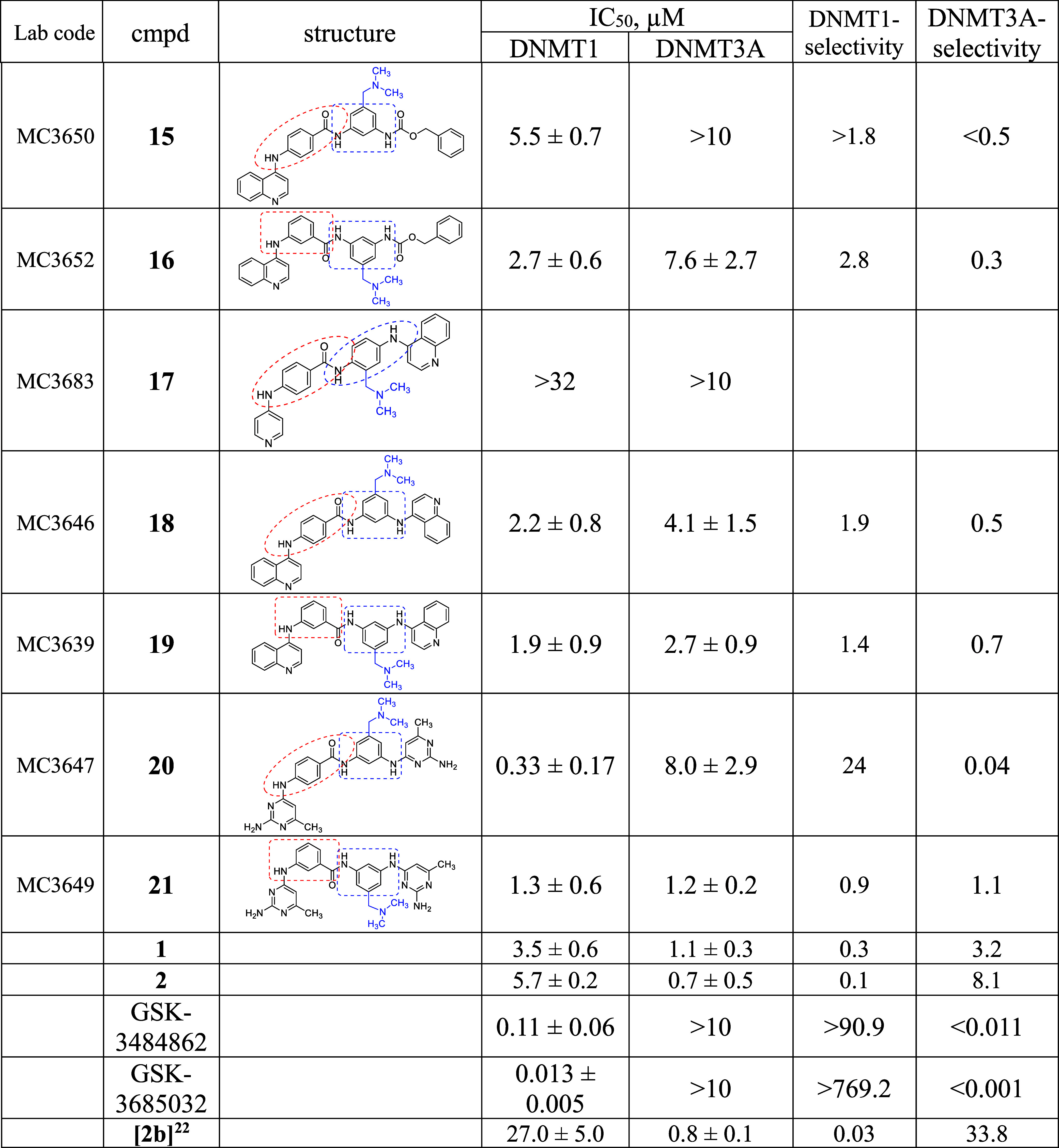
IC_50_ Values of Compounds **4**–**21** against DNMT1 and DNMT3*A*/3L Through the Detection of Direct DNA Methylation

In contrast, the presence of a basic side chain in prototype **2** produced potent DNMT inhibitors (**7**–**14**), with some selectivity for DNMT3A (**10**, **11**) or DNMT1 (**14**). Specifically, introducing the dimethylaminomethyl chain at ring B of **2** yielded compound **7**, potent at submicromolar level against both the DNMTs. However, inverting the amide function between the B/C rings (compound **8**) slightly decreased its potency. Among **2**-analogs with a basic chain at ring C, the most potent DNMT1/3A inhibitors were compound **9** (which contained a dimethylamino group and exhibited low submicromolar potency against both enzymes) and compound **10** (which replaced the dimethylamino group with a dimethylaminomethyl moiety, making it 10-fold more potent against DNMT3A and 10-fold less potent against DNMT1 than **9**, thereby achieving >100-fold selectivity for DNMT3A). Reducing the amide function between the B/C rings further diminished potency (compare **11** with **10**). Meanwhile, inserting (*N*-methylpiperazin-1-yl)­methyl (compound **12**) or (1*H*-imidazole-1-yl)­methyl (compound **13**) side chains produced highly potent inhibitors with nanomolar activity against both DNMTs. Finally, introducing two dimethylaminomethyl groups at rings B and C (compound **14**) maintained nanomolar potency against DNMT1 while drastically reducing activity against DNMT3A, making **14** a potent DNMT1-selective inhibitor (selectivity index >227 for DNMT1). To confirm its high selectivity toward DNMT1, **14** was also tested against G9a and PRMT1, as representative samples of lysine and arginine methyltransferases, respectively. The IC_50_ values of **14** were >50 μM for G9a, with selectivity index >1136, and >100 μM for PRMT1, with selectivity index >2272 (Table S2, Supporting Information).

The two analogs of compound **3** featuring a basic chain at ring C (**15** and **16**) displayed moderate activity against DNMT1 (**15**) or both DNMTs (**16**). In the *bis*-quinoline series, inserting a basic chain at ring C while replacing the quinoline with a pyridine ring reduced DNMT inhibition (**17**). However, reintroducing the quinoline ring restored activity, yielding compounds **18** and **19**, which were effective at low micromolar levels. The *meta*/*meta*-oriented **19** exhibited slightly greater potency than its *para*/*meta* analog **18**.

In the *bis*-pyrimidine series, adding a basic chain at ring C resulted in compounds **20** and **21**. Compound **20** showed submicromolar potency against DNMT1 and micromolar potency against DNMT3A, with a DNMT1 selectivity index of 24. Meanwhile, compound **21** displayed similar potency (∼1 μM) against both enzymes.

### Effect of Quinoline-Based DNMT Inhibitors on DNA Methylation in Cells

To assess the impact of the inhibitors on the DNA methylation levels in cells, we tested compounds **4**–**21** using a luciferase reporter construct under the control of a methylated cytomegalovirus (CMV) promoter integrated in KG-1 leukemia cells (CMV-luc assay).^22,23^ Previous studies by our group^21^ and others^32^ reported difficulties in detecting cellular hypomethylation with the lead compound **2** during 24–72 h of treatment, and several compounds from the **4**–**21** series failed to reduce methylation levels in murine embryonic stem cells (48 h of treatment).^24^ When tested in KG-1 cells at 3.2 μM for 24 h, most compounds **4**–**21** did not reactivate luciferase expression, confirming their inability to induce DNA hypomethylation in cells (data not shown). This could be attributed to their pharmacokinetic properties and/or their ability to inhibit other DNA-interacting enzymes,^24^ but also to the short duration of the experiment (24 h), during which only active DNA demethylation would be expected.^34^

Next, we examined selected compounds **7**, **9**, **10**, **12**–**14**, with IC_50_ values ≤150 nM against at least one DNMT, along with the structurally related prototype **2**, for their ability to demethylate an endogenous tumor suppressor gene in a solid cancer model, the colon carcinoma HCT-116 cell line. Compound **10** was chosen for its selectivity toward DNMT3*A*/3L, while compound **14** was selected for its DNMT1 selectivity. Compounds **12** and **13** were included because they exhibited high potency against both DNMT1 and DNMT3*A*/3L. We employed the Combined Bisulfite Restriction Analysis (COBRA) assay^27^ to monitor the methylation status of P16^INK4A^, a tumor suppressor gene reactivated by DNMT inhibition/deletion,^26^ and Long Interspersed Nucleotide Element 1 (LINE-1) repeats, a well-established surrogate measure of global DNA methylation.^35^ HCT-116 cells were treated for 10 days, with media changes and fresh compound additions every 3 days to allow for passive DNA demethylation and dilution of the DNA methylation signal.^34^ Decitabine (5-azadC) was used as a positive control. As shown in Figure 2, after 10 days of treatment at 0.32 and 1 μM, compound **12**, which demonstrated strong in vitro activity against both DNMT1 and DNMT3*A*/3L (Table 1), was the most effective in this assay specifically decreasing P16 methylation while having no effect on LINE-1 methylation. Compounds **7**, **9**, and **10** gave only slight effect in the same assay. Prototype **2** exhibited no hypomethylating effect on either P16 or LINE-1. The reference compound 5-azadC reduced both P16 (by approximately 40–50%) and LINE-1 (60%) methylation levels, confirming its broad, nonspecific demethylating activity. Compound **13** failed to reduce methylation levels of either LINE-1 or P16, while **14** was tested only at 0.32 μM due to its high cytotoxicity at higher concentration (Figure 2, and see below).

**Figure 2:**
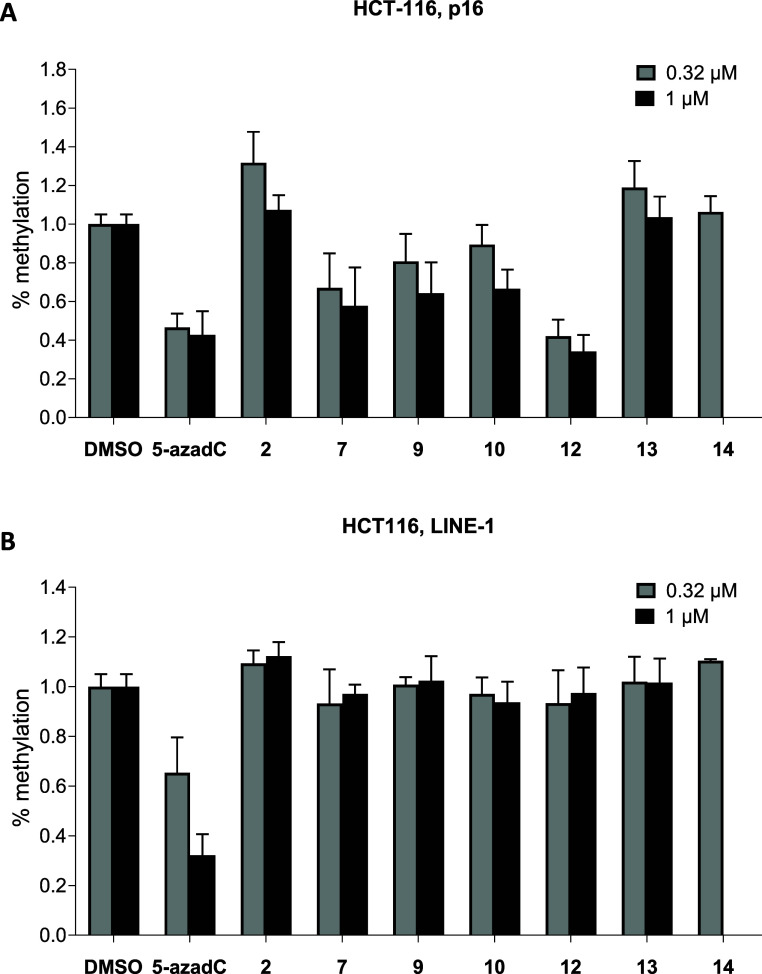
Effects of **2**, **7**, **9**, **10**, **12**, **13**, and **14** on the methylation status of the tumor suppressor gene P16^INK4A^ (**A**) and of the gene LINE-1 (global DNA) (**B**) in HCT-116 cells after 10 days of treatment at 0.32 and 1 μM.

### DNA Thermal Denaturation Experiments in the Presence of Quinoline Compounds

The previous studies by our group demonstrated that quinoline-based compounds **7**, **9**, **10**, **12**–**14** inhibit not only DNMTs but also DNA-interacting enzymes. Additionally, crystallographic experiments showed that compound **10** (**9** in ref.) binds in the minor groove of DNA.^24^ To investigate the potential mechanism underlying this broad inhibition activity, we conducted DNA thermal denaturation experiments, measuring changes in DNA absorbance as the transition occurs from the hairpin duplex state (at low temperature) to unfolded single-stranded DNA (at high temperature).^28^ Representative compounds **7**, **10**, **12**, and **14** were tested at 10 μM using a 2 μM DNA duplex, while compound **8**, a potent DNMT inhibitor (IC_50_ = 1–2 μM, see Table 1) but a weak inhibitor of other DNA-interacting enzymes,^24^ was included for comparison. Table 2 reports the differences in the melting temperature (*T*_m_) of the DNA duplex in the presence and in the absence of the tested compounds (Δ*T*_m_). The Δ*T*_m_ data indicate that **7**, **10**, **12**, and **14** significantly stabilized the DNA duplex, suggesting strong DNA interaction, whereas **8** did not (Figure 3). Structural analysis of **7** [*N*-(3-((2-amino-6-methylpyrimidin-4-yl)­amino)­phenyl)-3-((dimethylamino)­methyl)-5-(quinolin-4-ylamino)­benzamide] revealed that replacing the amide linker connecting the B/C rings with an inverted amide (compound **8**) abolished DNA binding ability and eliminated broad inhibition of DNA-interacting enzymes while maintaining DNMT inhibition. The prototype compounds **1** and **2** were previously tested under the same DNA thermal denaturation conditions and exhibited weak (**1**) or moderate (**2**) DNA binding properties.^31^ Both compounds were also weak or inactive against adenine methyltransferases.^24^

**Table 2:** Δ*T*_m_ Values Determined for **7**, **8**, **10**, **12**, and **14** at 10 μM Using the DNA Duplex at 2 μM

lab code	compd	Δ*T*_m_ (°C)
MC3716	**7**	11.3
MC3653	**8**	0.7
MC3563	**10**	12.5
MC3669	**12**	8.2
MC3817	**14**	15.8
SGI	**1**	1.0^a^
MC3343	**2**	3.0^a^

a Ref 31.

**Figure 3:**
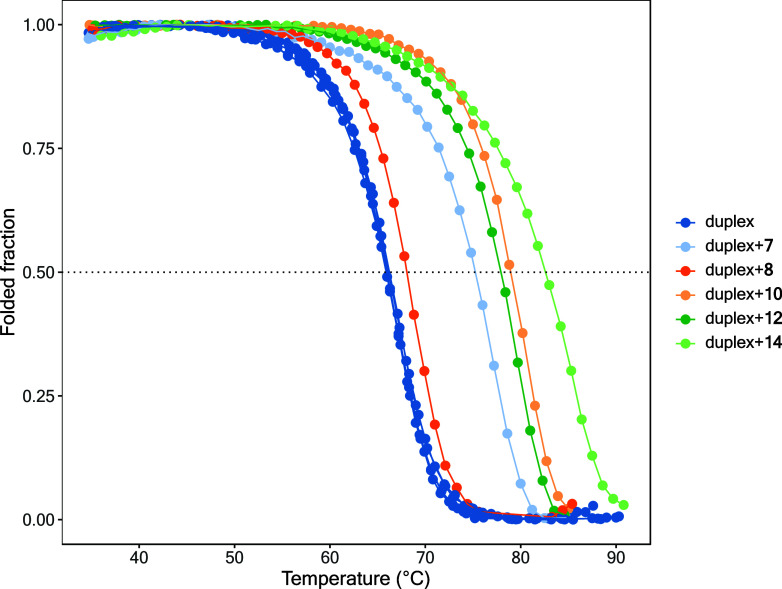
DNA thermal denaturation experiments performed on **7**, **8**, **10**, **12**, and **14** at 10 μM.

### Anticancer Effects of Selected Quinoline Derivatives

To evaluate their antiproliferative potential, selected compounds **7**, **9, 10**, **12**–**14** were tested against a panel of cancer cells, including A549 and H460 (nonsmall cell lung cancer), HCT-116 (colon cancer), HeLa (cervical cancer), MDA-MB-231 (triple-negative breast cancer), and U937 (histiocytic lymphoma). Prototype **2** was used for comparison. Cells were treated with increasing concentrations of each compound (0.05–100 μM) for 48 h, after which cell viability was assessed using the 3-(4,5-dimethylthiazol-2-yl)-2,5-diphenyltetrazolium bromide (MTT)-based colorimetric assay. The IC_50_ values for each compound in all cancer cell lines were determined. Figure 4 displays the proliferation inhibition curves, while Table 3 summarizes the IC_50_ values. Under these conditions, most compounds exhibited similar or higher potency than **2** in inhibiting cancer cell proliferation, mainly in specific cancer cell lines (HeLa, MDA-MB-231) where **2** was less effective. In detail, against the triple-negative breast cancer MDA-MB-231 cells, compounds **9**, **10**, **12**, and **14** demonstrated significantly enhanced potency, with IC_50_ values in the single-digit micromolar range (1.0–8.8 μM). Compound **9** was 35-fold more potent than **2**, while **10**, **12**, and **14** were 8-, 4-, and 19-fold more potent, respectively. Against HeLa cervical cancer, compound **14** was 4-fold more potent than **2**, exhibiting an IC_50_ of 4.2 μM. In A549 nonsmall cell lung cancer cells, compounds **9** and **14** displayed slightly increased potency than **2**. The most sensitive cell line was the HCT-116 colon cancer cell line, with **2** showing an IC_50_ of 2.3 μM. Compounds **7**, **9**, and **10** displayed similar potency, whereas **14** was 5-fold more effective, reaching submicromolar concentration (IC_50_ = 0.4 μM), in agreement with the COBRA analysis results (see above).

**Figure 4:**
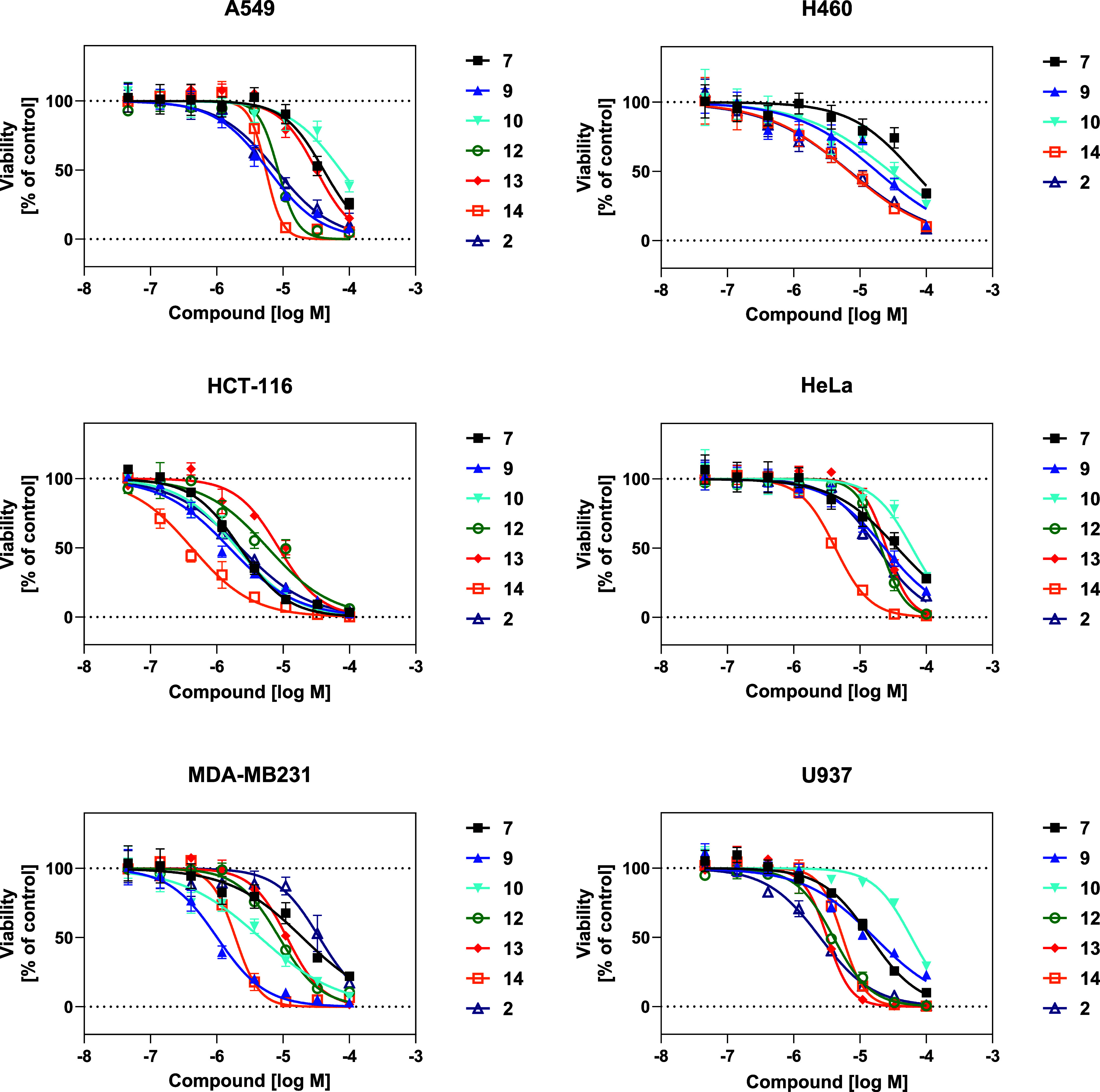
Dose-dependent effect curves of compounds **7**, **9**, **10**, **12–14**, along with the prototype **2**, on cell viability in A549, H460, HCT-116, HeLa, MDA-MB-231, and U937 cell lines upon 48 h of treatment. All experiments were performed in sextuplicate ±SD (standard deviation).

**Table 3:** Antiproliferative Activity (48 h Treatment) of Novel Quinoline-Based DNA-Targeting Enzyme Inhibitors against a Panel of Human Cancer Cell Lines

compd	antiproliferative activity (IC_50_, μM)^a^					
	A549 nonsmall cell lung cancer	H460 nonsmall cell lung cancer	HCT-116 colon cancer	HeLa cervical cancer	MDA-MB-231 triple negative breast cancer	U937 histiocytic lymphoma
**7**	41 ± 4	63 ± 12	2.2 ± 0.2	37 ± 4	20 ± 3	14 ± 2
**9**	6.0 ± 0.6	18 ± 5	1.5 ± 0.2	24 ± 1	1.0 ± 0.1	16 ± 3
**10**	74 ± 12	29 ± 5	2.0 ± 0.1	61 ± 9	4.5 ± 0.7	60 ± 8
**12**	8.5 ± 0.7	ND^b^	5.9 ± 1.2	21 ± 1	8.8 ± 0.7	4.0 ± 0.3
**13**	32 ± 4	ND	8.6 ± 1.4	25 ± 2	11.5 ± 0.8	3.2 ± 0.2
**14**	5.5 ± 0.5	6.8 ± 0.7	0.4 ± 0.1	4.2 ± 0.2	1.9 ± 0.2	5.5 ± 0.2
**2**	7.9 ± 0.9	6.9 ± 1.2	2.3 ± 0.2	18 ± 2	35 ± 5	2.5 ± 0.2

a The reported results represent the average ±SD (standard deviation) of sextuplicate experiments.

b ND, not determined.

### Cytotoxic Effects of Compounds **9** and **14** in Colorectal Cancer Cells

When tested in HCT-116 colorectal cancer cell line, compounds **9** and **14** displayed potent cytotoxic effects, with IC_50_ values of 1.5 and 0.4 μM, respectively, after 48 h of treatment (Table 3). The same two compounds **9** and **14**, the first equally potent against both the DNMT isoforms and the latter selective for DNMT1 (Table 1), were the most potent also against MDA-MB-231 cell line (Table 3), which expresses comparable levels of DNMT1 and DNMT3A to HCT-116 cells (Human Protein Atlas data, Table S3 in Supporting Information). Compound **17**, practically inactive against both DNMTs (Table 1), when tested in HCT-116 cells for 48 h displayed no cytotoxicity up to 2 μM (Figure S7, Supporting Information). To assess selectivity, compound **14** was tested against two noncancerous cell lines, retinal pigment epithelium (RPE) cells and BJ human fibroblasts. It displayed IC_50_ values of 5.0 and 8.5 μM, respectively, after 48 h (Figure S8, Supporting Information), showing a 12- to 22-fold selectivity window compared to HCT-116 cells.

### Cell Cycle and Apoptosis Analysis

Compounds **9** and **14** were further analyzed by fluorescence-activated cell sorting (FACS) to evaluate their effects on the HCT-116 cell cycle. Figure 5A–C present representative FACS histograms (Figure 5A) and relative quantification (Figure 5B,C) of HCT-116 cells treated with **9** and **14** compared to control cultures. At 2 μM, compound **9** induced a significant G0/G1 accumulation, shifting the proportion of cells in this phase from 47.3% to 70.6% after 24 h. Conversely, **14** caused a block in the S/G2M phase associated with apoptotic cell death, affecting over 50% of the cells after 48 h, as indicated by the pre-G1 peak (Figure 5C). Compound **17**, used as negative control, at 2 μM after 48 h showed a similar trend to the untreated sample (DMSO) (Figure S9, Supporting Information). The induction of apoptosis was further confirmed by Western blot analysis, showing a time-dependent increase in cleaved PARP levels in HCT-116 cells treated with **14** (2 μM) for 24 and 48 h (Figure 5D).

**Figure 5:**
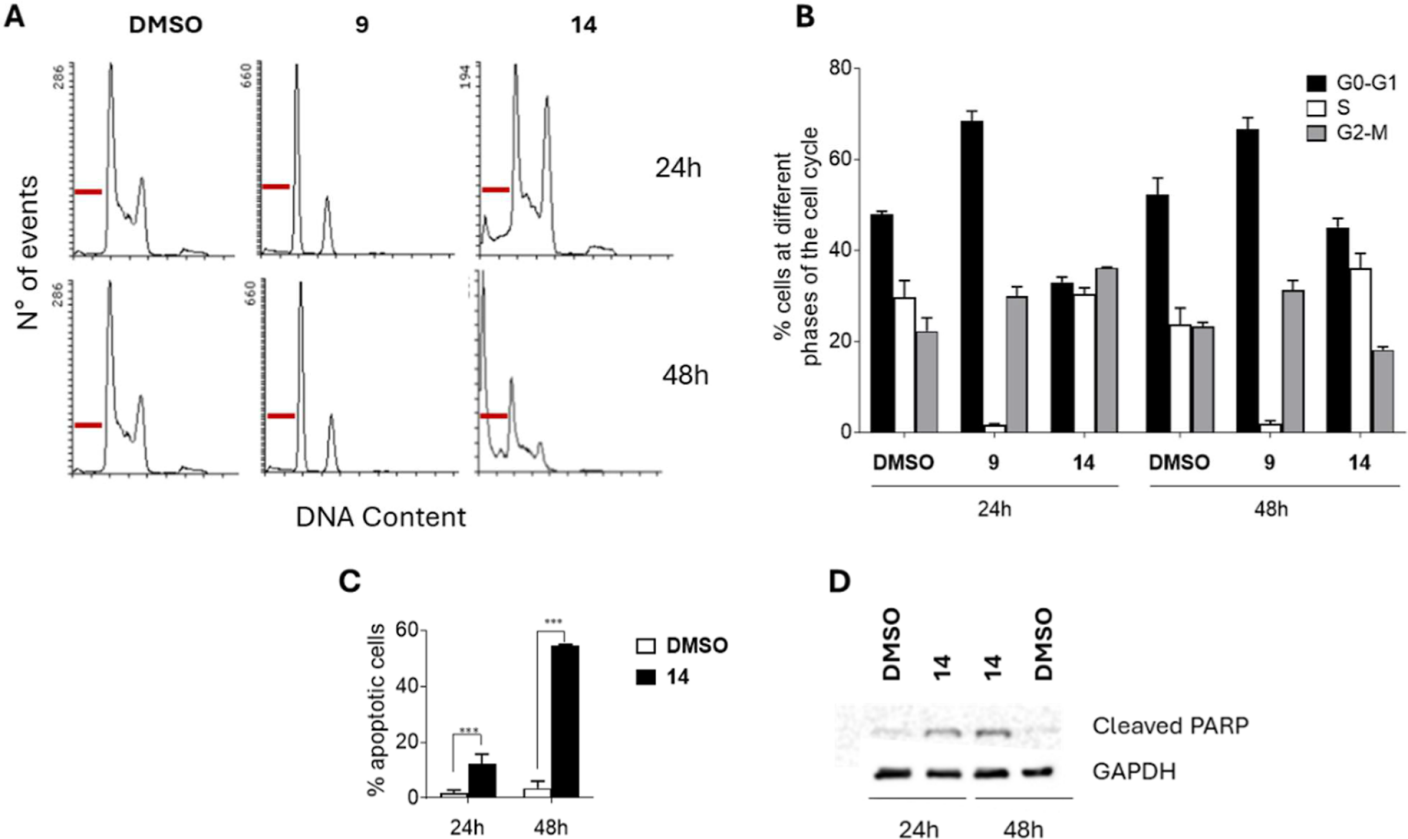
Effects of compounds **9** and **14** on HCT-116 cell cycle progression and apoptosis induction. (A,B) Representative histograms of DNA content (A) and relative quantification (B,C) using propidium iodide staining in HCT-116 cells exposed to **9** and **14** for the indicated time points. (A) Red bars indicate the pre-G1 peak. (D) Western blot analysis of cleaved PARP in HCT-116 cells exposed to **14** at the indicated time points. GAPDH was used as a loading control. Experiments were performed in duplicate (*n* = 2). Results are expressed as mean ± SD. Compounds were tested at 2 μM for 24 and 48 h, as indicated.

### Live Cell Imaging and Mitotic Progression Analysis

To further investigate these findings, we performed live-cell imaging of HCT-116 cultures exposed to **9** and **14** (2 μM), monitoring individual cell fates from the moment of drug administration. In untreated (DMSO) cells, mitosis was typically completed within 40 min, with cells entering a subsequent mitotic cycle 24 h later. Of the 79 cells treated with **9**, 22 entered mitosis. However, most exhibited a prolonged metaphase arrest. Among these, a small fraction (∼2%) underwent cell death, while others ceased cell cycle progression irreversibly after mitosis. In contrast, only 5 of the 66 cells exposed to **14** initiated mitosis, and all showed a considerable delay in completion time. The majority of **14**-treated cells underwent widespread apoptosis in interphase, primarily characterized by extensive membrane blebbing (Figure 6).

**Figure 6:**
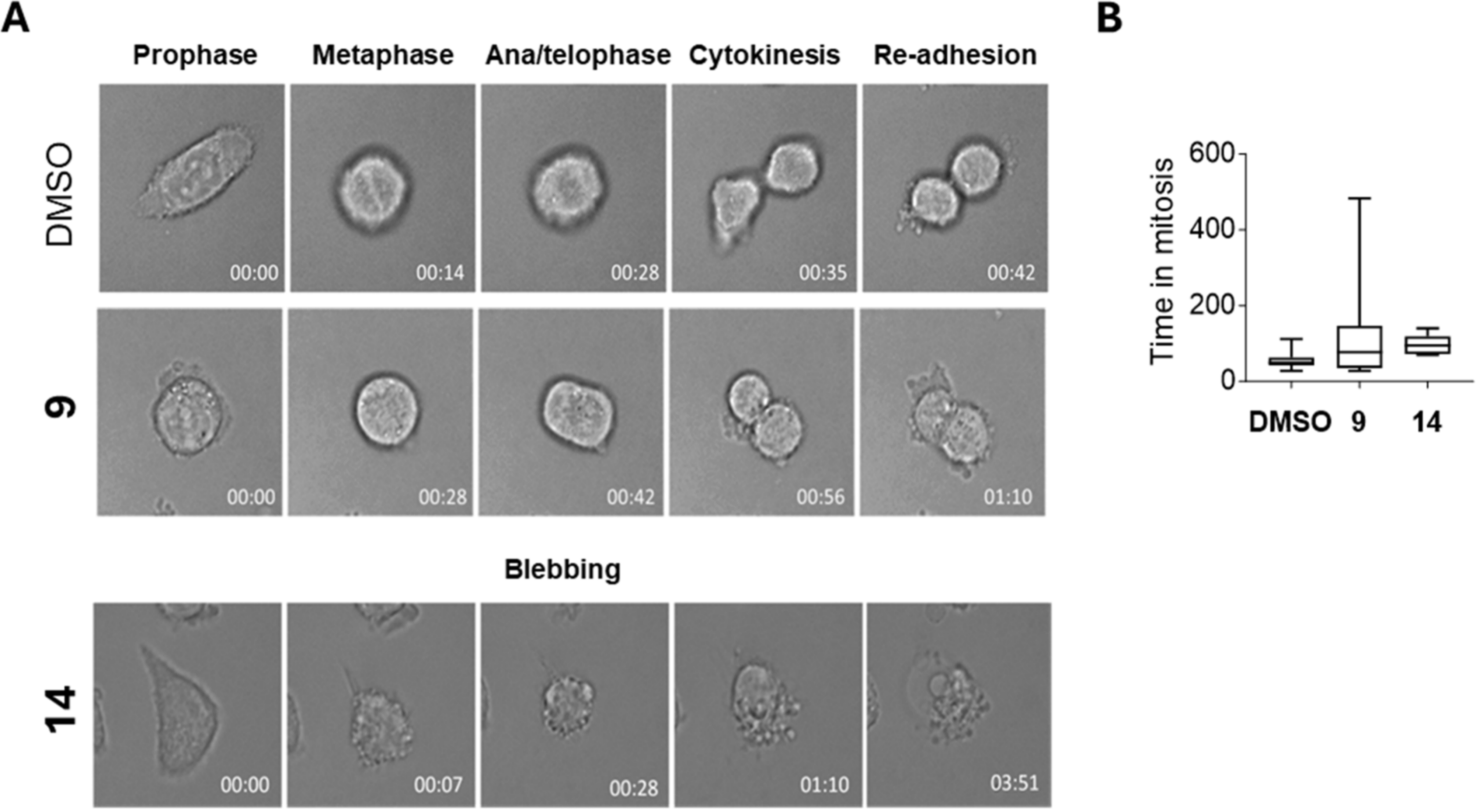
Live-cell imaging of HCT-116 cultures exposed to **9** and **14** (2 μM). (A) Still images of representative control (DMSO) and **9**-treated cells, or **14**-treated cells undergoing apoptotic cell death. Time is given in h/min. Cells undergoing mitosis (DMSO or **9**) or starting apoptosis (**14**) are shown at time 00.00. (B) Box-plots with whiskers showing the time spent in mitosis. DMSO: *n* = 93 cells; **9**: *n* = 79 cells; **14**: *n* = 66 cells.

### DNA Damage Response and p53 Activation

To assess whether **14** induces DNA damage, we examined H2AX phosphorylation (γH2AX)—a marker of DNA strand breaks—and P53 activation in HCT-116 cells following 16–48 h of treatment. Western blot analysis revealed a dose-dependent increase in γH2AX and p53 accumulation upon exposure to **14**, indicating P53 stabilization and activation in response to DNA damage (Figure 7). These results suggest that **14** triggers DNA damage and activates the p53/γH2AX pathway, leading to cell-cycle arrest and apoptosis in HCT-116 cells.

**Figure 7:**
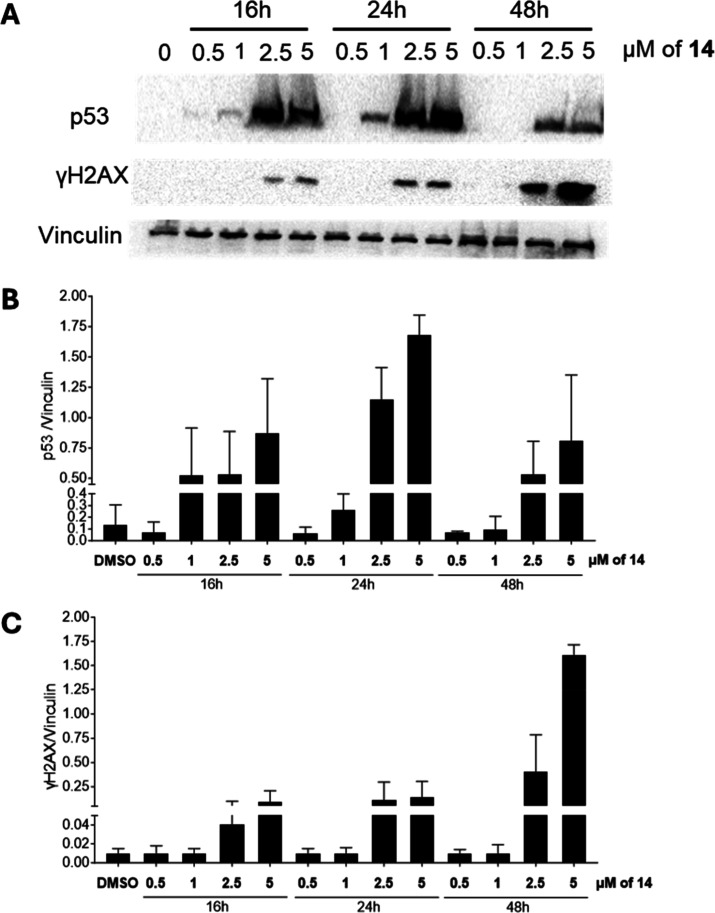
Western blot analysis and relative quantification of p53 and H2AX phosphorylation (γH2AX) in HCT-116 cell cultures exposed to **14** for the indicated doses and time points.

### P53-dependent Effects of Compound 14 in HCT-116 Cells

To confirm the role of P53 in the cellular response to **14**, isogenic variants of HCT-116 human colon cancer cells, including P53^–/–^ (P53-null) cells, were utilized. First, an MTT assay was performed on both P53 WT and P53^–/–^ HCT-116 cells to assess the effects of **14** on cell viability (Figure 8A). Notably, a significantly lower level of cell growth and viability was observed in HCT-116 P53^+/+^ cells compared with HCT-116 P53^–/–^ cells. To confirm whether this effect was attributed to apoptosis induced by **14**, Annexin V flow cytometry analysis was performed on both wild-type and P53-null cells. As shown in Figure 8B, upon exposure to 2.5 μM of **14** for 48 h, significant PI and Annexin V fluorescence was detected, indicating early apoptosis (lower right quadrant) and late apoptosis/necrosis (upper right quadrant). These results confirm that **14** induces apoptotic cell death pathways in both cell lines. However, a much higher level of apoptosis was detected in HCT-116 P53 WT cells compared with HCT-116 P53^–/–^ cells. Finally, Western blot analysis of cleaved Caspase 3, P53 accumulation, and γH2AX levels (Figure 8C) further confirmed that **14** induces DNA damage, leading to cell-cycle arrest and apoptosis in HCT-116 cells in a P53-dependent manner.

**Figure 8:**
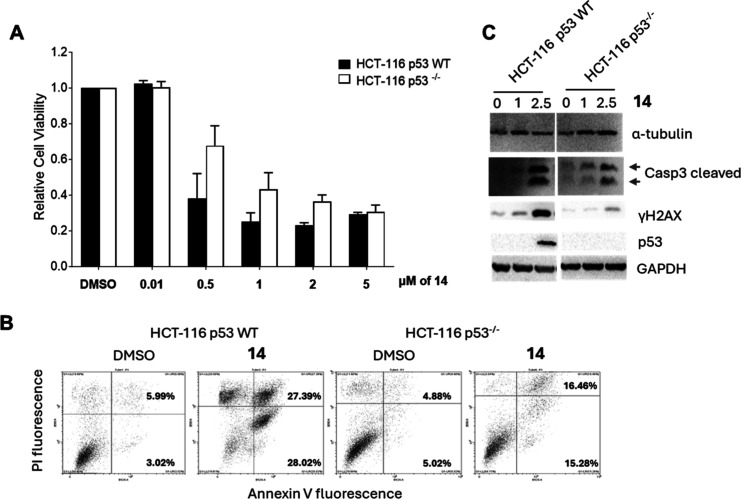
P53-dependent effects of **14** on cell viability and apoptosis in HCT-116 cells. (A) Effect of **14** on cell viability of P53 WT and P53-null HCT-116 cells. One day after seeding, cells were treated with increasing concentrations of **14** for 48 h, and proliferation was measured by using the MTT assay. Results are expressed as mean ± SE (*n* = 2) and are presented as a percentage of vehicle-treated controls. (B) Annexin V/PI staining to assess apoptosis in cells treated with **14** (2.5 μM for 48 h), analyzed via flow cytometry. (C) Western blot analysis of cleaved Caspase 3, P53, and γH2AX in HCT-116 cells treated with **14** for 48 h. Representative results from three independent experiments are shown. α-Tubulin and GAPDH were used as loading controls.

Since some quinoline analogs have been shown to induce DNMT protein degradation in addition to enzymatic inhibition,^20–22^ we investigated the effect of **14** on DNMT1 and DNMT3A protein level in HCT-116 cells, in comparison with MS9024, a recently reported specific DNMT1 degrader.^36^ When tested at 0.1, 0.5, and 1 μM for 24 h, **14** failed to induce degradation of either DNMT proteins, whereas MS9024 selectively reduced DNMT1 levels, as expected (Figure S10, Supporting Information).

## Discussion and Conclusions

Quinoline-based compounds represent a well-established class of small-molecule DNMTi with significant anticancer activity across various cancer models. Some of these compounds have been shown to induce DNMT protein degradation in addition to enzymatic inhibition,^20–22^ while others inhibit DNA-interacting enzymes, including DNA glycosylases and DNA polymerases, potentially contributing to their anticancer effects.^24^

Here, we provide a detailed report on the design and synthesis of our latest series of quinoline-based DNMTi (**4**–**21**, Figure 1), incorporating basic side chains to improve water solubility and biological activity. These new compounds were developed based on prototypes **1** (compounds **4**–**6**), **2** (compounds **7**–**14**), **3** (compounds **15**, **16**), as well as their *bis*-quinoline (compounds **17**–**19**) and *bis*-pyrimidine (compounds **20**, **21**) analogs.

Compounds **4**–**21** were evaluated against human DNMT1 and human DNMT3Acat, through a direct measure of the methylation event, obtained by using a restriction enzyme-based methylation assay (Table 1).^30^ Among these, *para*/*para*-oriented compounds **4**–**6** exhibited a loss of DNMT inhibition compared to prototype **1**, while *meta*/*meta* compounds **7**–**14** demonstrated enhanced potency against one or both DNMT isoforms. Compound **10** was the most potent and selective DNMT3A inhibitor (IC_50_ = 0.019 μM, selectivity index = 105). Compound **14**, the only derivative with two basic chains, was the most potent and selective DNMT1 inhibitor (IC_50_ = 0.044 μM, selectivity index = >227). Compounds **15** and **16**, basic-chained analogs of **3**, were potent at the low micromolar level against DNMT1 (**15**) or both enzymes (**16**). *Bis*-quinoline analogs (**17**–**19**) exhibited variable potency, with **18** and **19** inhibiting DNMTs in the low micromolar range. *Bis*-pyrimidine analog **20** was highly potent against DNMT1 (submicromolar potency, selectivity index = 24), whereas **21** inhibited, at approximately 1 μM, both the two DNMT isoforms.

Previous studies, reported by us^21^ and others,^32^ reported unsuccessful detection of hypomethylation in cells treated with prototype **2**. Similarly, several derivatives in series **4**–**21** failed to reduce methylation in murine embryonic stem cells^24^ or reactivate luciferase expression in KG-1 leukemia cells (data not shown). Thus, compounds with IC_50_ ≤ 150 nM against at least one DNMT isoform (**7**, **9**, **10**, **12**–**14**) were further evaluated in HCT-116 colon cancer cells using the COBRA assay.^27^ After 10 days of treatment, compound **12** was the most effective to selectively demethylate P16^INK4A^ (about 60%), leaving LINE-1 levels unchanged (Figure 2). Decitabine (5-azadC), used as a positive control, reduced both the P16 and LINE-1 methylation, confirming its nonspecific demethylating activity.

Further DNA thermal denaturation experiments^28^ revealed that **7**, **10**, **12**, and **14** strongly bound to DNA and stabilized the DNA duplex, whereas **8**, an amide-inverted analog of **7**, did not (Figure 3, Table 2). Interestingly, **8**, like prototypes **1** and **2**,^31^ showed weaker DNA binding, and was less effective against other DNA-interacting enzymes than **7**, **10**, **12**, and **14**.^24^ The interference with a wide range of DNA-interacting enzymes might make it difficult to interpret cellular effects, but it could also be of added value for compounds with anticancer activity, since it covers different pathways related to the disease.

Compounds **7**, **9, 10**, and **12**–**14** were tested against a panel of cancer cell lines (A549, H460, HCT-116, HeLa, MDA-MB-231, and U937) in comparison with prototype **2** (Figure 4 and Table 3). Compounds **9**, **10**, and **14** exhibited stronger potency than **2**, particularly in MDA-MB-231 and HCT-116 cells. In HCT-116 cells, **9** and **14** displayed IC_50_ values of 1.5 μM and 0.4 μM, respectively, after 48 h. Compound **14** showed higher selectivity for cancer cells, with IC_50_ values against two noncancerous cell lines of 5.0 μM (RPE) and 8.5 μM (BJ fibroblasts), corresponding to a 12- to 22-fold selectivity window over HCT-116 cells.

Further studies in HCT-116 cells demonstrated that **9** caused a G0/G1 block, while **14** induced an S/G2M arrest and apoptosis (>50% pre-G1 peak, increased cleaved PARP levels, Figure 5). Live-cell imaging revealed that **14** caused extensive interphase apoptosis with membrane blebbing, whereas **9** induced prolonged metaphase arrest with limited cell death (Figure 6).

We recently reported that, in EWS, treatment with **2** gave DNA damage, as revealed by the induction of the γH2AX foci. Such an effect stimulated activation of P53-dependent signaling and apoptosis in P53 WT cells, while in P53-mutated cells persistent micronuclei and increased DNA instability were observed.^21^ Among **4**–**21**, the (*N*-methylpiperazin-1-yl)­methyl compound **12** determined arrest of proliferation in A549 human nonsmall cell lung carcinoma (NSCLC), joined to increased levels of γH2AX, P53, and P21 as a response to DNA damage. Differently, in P53-mutated or -deleted NSCLC cells we observed no changes in P53/P21 levels and little or no cytotoxicity.^24^

Here, we compare the effects of **14** in the HCT-116 P53 WT cell line and in its isogenic variant HCT-116 P53^–/–^ cell line, determining cytotoxicity, apoptosis induction, and stimulation of DNA damage. With respect to what was observed in HCT-116 P53 WT cells, in HCT-116 P53^–/–^ cells **14** showed reduced impact on cell viability, decreased apoptosis and lower levels of cleaved Caspase 3, P53, and γH2AX accumulation, confirming a P53-dependent mechanism of action in this cell line (Figures 7 and 8) linked to DNA damage.

Altogether, these data suggest that quinoline-based DNMT inhibitors represent a promising therapeutic strategy for targeting P53 WT tumors, leveraging P53 activation to induce DNA damage, cell-cycle arrest, and apoptosis.

## Experimental Section

### Chemistry

Melting points were determined on a Buchi 530 melting point apparatus and are uncorrected. ^1^H NMR spectra were recorded at 400 MHz on a Bruker AC 400 spectrometer; chemical shifts are reported in δ (ppm) units relative to the internal reference tetramethylsilane (Me_4_Si). All compounds were routinely checked by TLC and ^1^H NMR. TLC was performed on aluminum-backed silica gel plates (Merck DC, Alufolien Kieselgel 60 F_254_) with spots visualized by UV light. All solvents were reagent grade and, when necessary, were purified and dried by standard methods. Concentration of solutions after reactions and extractions involved the use of a rotary evaporator operating at a reduced pressure of ca. 20 Torr. Organic solutions were dried over anhydrous sodium sulfate. The purity of the final compounds **4**–**21** was analyzed by elemental analysis and for selected compounds (**7–9**, **12–14**) also by HPLC. The elemental analysis has been performed on Thermo Fisher FlashSmart CNHS/O. The HPLC system consisted of a Dionex UltiMate 3000 UHPLC (Thermo Fisher) system equipped with an automatic injector and column heater and coupled with a Diode Array Detector DAD-3000 (Thermo Fisher). The analytical controls were performed on a Hypersil GOLD C18 Selectivity 5 μm (4.6 × 250 mm) HPLC Column (Thermo Fisher) in gradient elution. Eluents: (A) H_2_O + 0.1% TFA; (B) CH_3_CN + 0.1% TFA. A 5 min isocratic elution at 10% solvent B was followed by a 15 min linear gradient elution from 10% to 90% solvent B and 5 min at 90% B. The flow rate was 1.0 mL/min, and the column was kept at a constant temperature of 30 °C. Samples were dissolved in their respective solvent A at a concentration of 1.0 mg/mL, and the injection volume was 1 μL. By analyzing the HPLC traces at both 254 and 280 nm, a chemical purity >96% was recorded for all molecules. Analytical results are within ±0.40% of the theoretical values. All chemicals were purchased from Sigma-Aldrich Chemistry, Milan (Italy), Fluorochem, Manchester (UK), Enamine, Kyiv (Ukraine), or from AlfaAesar, Karlsruhe (Germany), and were of the highest purity.

#### General Procedure for the Synthesis of the Final Compounds **4–7**, **15–17**, and of the Amide Intermediates **38**, **40a**–**c**, **41**, **43**, **50**, and **51**

Triethylamine (3.98 mmol, 8.5 equiv) and PyBOP (0.57 mmol, 1.2 equiv) were added to a solution of the appropriate benzoic acid **25a,b**, **30**,^19^ **33**,^19^ **37**,^19^ **39**,^19^ or **49** ^29^ (0.47 mmol, 1 equiv) in dry DMF (3 mL) under a nitrogen atmosphere. The resulting mixture was stirred for 30 min at room temperature (rt); afterward, the appropriate aniline **26a,b**,^19^ **36a**–**c**, or **42** (0.47 mmol, 1 equiv) was added under a nitrogen atmosphere, and the reaction was stirred overnight. After, the reaction was quenched with water (50 mL) and extracted with ethyl acetate (3 × 30 mL). The combined organic extracts were dried over sodium sulfate, and the residue obtained upon solvent evaporation was purified by column chromatography (SiO_2_ eluting with chloroform/methanol/NH_3_:10/1/0.2) to provide the pure product **4–7**, **1–17**, **38**, **40a**-**c**, **41**, **43**, **50** or **51**.

#### *N*-(4-((2-amino-6-methylpyrimidin-4-yl)­amino)­phenyl)-2-((dimethylamino)­methyl)-4-(quinolin-4-ylamino)­benzamide hydrochloride (**4**, MC3671)

Prepared starting from **25a** (0.47 mmol, 0.15 g) and **26a** ^19^ (0.47 mmol, 0.10 g). Mp: 198–200 °C (acetonitrile/methanol); yield: 71%. ^1^H NMR (400 MHz; DMSO-*d*_6_) δ: 2.28 (s, 3H, –CH_3_), 2.83 (s, 6H, –N­(CH_3_)_2_), 4.49 (s, 2H, –CH_2_N­(CH_3_)_2_), 6.24 (s, 1H, pyrimidine proton), 7.14 (d, 1H, *J* = 7.2 Hz, quinoline proton), 7.80–7.88 (m, 6H, benzene and quinoline protons), 7.97 (s, 1H, benzene proton), 8.06–8.10 (m, 2H, benzene proton), 8.15–8.18 (m, 1H, benzene proton), 8.68 (d, 1H, *J* = 6.8 Hz, quinoline proton), 9.00 (d, 1H, *J* = 8.8 Hz, quinoline proton), 9.43 (br s, 1H, –NH–pyrimidine) 10.86 (br s, 1H, NH- quinoline), 10.91 (br s, 2H, –CONH−), 11.42 (br s, 1H, hydrochloride), 12.83 (br s, 1H, hydrochloride), 14.99 (br s, 1H, hydrochloride) ppm. ^13^C NMR (100 MHz, DMSO-*d*_6_) δ: 23.8, 44.8 (2C), 61.6, 97.5, 105.5, 117.1, 118.5, 119.8, 120.1 (2C), 123.3, 124.2 (2C), 125.5, 126.3, 127.3, 128.3, 131.0, 133.7, 135.0, 137.5, 144.2, 145.5, 147.3, 148.0, 158.2, 162.4, 166.0, 166.5.

#### 4-((2-Amino-6-methylpyrimidin-4-yl)­amino)-*N*-(2-((dimethylamino)­methyl)-4-(quinolin-4-ylamino)­phenyl)­benzamide hydrochloride (**5**, MC3682)

Prepared starting from **29** (0.47 mmol, 0.14 g) and **30** ^19^ (0.47 mmol, 0.11 g). Mp: 239–241 °C (methanol); yield: 76%. ^1^H NMR (400 MHz, DMSO-*d*_6_) δ: 2.32 (s, 3H, –CH_3_), 2.77 (s, 6H, –N­(CH_3_)_2_), 4.36 (s, 2H, –CH_2_N­(CH_3_)_2_), 6.39 (s, 1H, pyrimidine proton), 7.07 (d, 1H, *J* = 6.4 Hz, quinoline proton), 7.66 (s, 2H, benzene proton), 7.82–7.93 (m, 2H, quinoline protons), 8.05–8.21 (m, 6H, benzene and quinoline protons), 8.57 (d, 1H, *J* = 6.8 Hz, quinoline proton), 8.94 (d, 1H, *J* = 8.4 Hz, quinoline proton), 10.62 (br s, 1H, –NH–pyrimidine) 11.22–28 (bm, 3H, NH-quinoline, −CONH-, hydrochloride), 13.15 (br s, 1H, hydrochloride), 14.87 (br s, 1H, hydrochloride) ppm. ^13^C NMR (100 MHz, DMSO-*d*_6_) δ: 23.8, 44.8 (2C), 60.8, 97.5, 105.5, 117.5 (2C), 119.6, 119.9, 120.1, 121.0, 123.3, 123.9, 125.5, 126.3, 126.9, 128.3, 129.9 (2C), 135.8, 136.8, 143.9, 145.6, 147.3, 148.0, 158.2, 162.4, 166.0, 166.5.

#### *N*-(4-((2-Amino-6-methylpyrimidin-4-yl)­amino)-2-((dimethylamino)­methyl)­phenyl)-4-(quinolin-4-ylamino)­benzamide hydrochloride (**6**, MC3672)

Prepared starting from **32** (0.47 mmol, 0.13 g) and **33** ^19^ (0.47 mmol, 0.12 g). Mp: 218–220 °C (acetonitrile/methanol); yield: 68%. ^1^H NMR (400 MHz, DMSO-*d*_6_) δ: 2.32 (s, 3H, –CH_3_), 2.78 (s, 6H, –N­(CH_3_)_2_), 4.37 (s, 2H, –CH_2_N­(CH_3_)_2_), 6.30 (s, 1H, pyrimidine proton), 7.01 (d, 1H, *J* = 7.2 Hz, quinoline proton), 7.47 (d, 1H, *J* = 8.8 Hz, benzene protons), 7.68–7.72 (m, 2H, benzene protons), 7.87 (t, 1H, *J* = 7.6 Hz, quinoline proton), 8.05–8.15 (m, 2H, benzene and quinoline protons),8.35–8.37 (m, 3H, benzene and quinoline protons), 8.63 (d, 1H, *J* = 6.8 Hz, quinoline proton), 8.93 (d, 1H, *J* = 8.4 Hz, quinoline proton), 10.60 (br s, 1H, –NH–pyrimidine) 10.91 (br s, 1H, NH-quinoline), 11.02 (br s, 1H, –CONH−), 11.25 (br s, 1H, hydrochloride), 12.91 (br s, 1H, hydrochloride), 14.89 (br s, 1H, hydrochloride) ppm. ^13^C NMR (100 MHz, DMSO-*d*_6_) δ: 23.8, 44.8 (2C), 60.8, 97.4, 105.5, 118.0 (2C), 119.3, 119.9, 120.3, 121.3, 123.3, 123.9, 125.5, 126.3, 128.0, 128.3, 130.1 (2C), 135.9, 136.6, 145.1, 145.6, 147.3, 148.0, 158.2, 162.4, 166.0, 166.5.

#### *N*-(3-((2-Amino-6-methylpyrimidin-4-yl)­amino)­phenyl)-3-((dimethylamino)­methyl)-5-(quinolin-4-ylamino)­benzamide hydrochloride (**7**, MC3716)

Prepared starting from **25b** (0.47 mmol, 0.15 g) and **26b** ^19^ (0.47 mmol, 0.10 g). Mp: 182–184 °C (acetonitrile/methanol); yield: 67%. ^1^H NMR (400 MHz, DMSO-*d*_6_) δ: 2.29 (s, 3H, –CH_3_), 2.82 (s, 6H, –N­(CH_3_)_2_), 4.48 (s, 2H, –CH_2_N­(CH_3_)_2_), 6.24 (s, 1H, pyrimidine proton), 7.19 (d, 1H, *J* = 6.8 Hz, quinoline proton), 7.37 (t, 1H, *J* = 8.0 Hz, benzene proton), 7.53–7.56 (m, 1H, benzene proton), 7.76 (d, 1H, *J* = 8.4 Hz, benzene proton), 7.86 (t, 1H, *J* = 7.6 Hz, quinoline proton), 7.97 (s, 1H, benzene proton), 8.05–8.18 (m, 3H, benzene and quinoline protons), 8.40–8.44 (m, 2H, quinoline protons), 8.58 (d, 1H, *J* = 6.8 Hz, quinoline proton), 8.92 (d, 1H, *J* = 8.8 Hz, quinoline proton), 10.64 (br s, 1H, –NH–pyrimidine), 10.76 (br s, 1H, NH-quinoline), 11.03 (br s, 1H, –CONH−), 11.27 (br s, 1H, hydrochloride), 12.86 (br s, 1H, hydrochloride), 14.80 (br s, 1H, hydrochloride) ppm. ^13^C NMR (100 MHz, DMSO-*d*_6_) δ: 23.8, 45.2 (2C), 63.3, 97.4, 105.5, 109.4, 116.3, 116.8, 117.6, 119.9, 120.4, 123.3, 125.5, 125.5, 126.3, 128.3, 128.8, 133.2, 139.3, 140.0, 141.8, 142.4, 145.7, 147.3, 148.0, 158.1, 162.4, 166.0, 166.0. HPLC: 98.2% pure (retention time 12.08 min) (Supporting Information Figure S1).

#### Benzyl (3-((dimethylamino)­methyl)-5-(4-(quinolin-4-ylamino)­benzamido)­phenyl)­carbamate hydrochloride (**15**, MC3650)

Prepared starting from **33** ^19^ (0.47 mmol, 0.12 g) and **48** (0.47 mmol, 0.14 g). Mp: 154–156 °C (acetonitrile); yield: 70%. ^1^H NMR (400 MHz, DMSO-*d*_6_) δ: 2.71 (s, 6H, –N­(CH_3_)_2_), 4.23 (s, 2H, –CH_2_N­(CH_3_)_2_), 5.16 (s, 2H, –CH_2_−), 7.20 (d, 1H, *J* = 5.2 Hz, quinoline proton), 7.35–7.49 (m, 8H, benzene and quinoline protons), 7.60 (d, 1H, *J* = 7.2 Hz, quinoline proton), 7.67–7.76 (m, 3H, benzene and quinoline protons), 7.94 (d, 1H, *J* = 8.0 Hz, benzene proton), 8.00 (d, 1H, *J* = 8.4 Hz, quinoline proton benzene proton), 8.38 (d, 1H, *J* = 8.4 Hz, quinoline proton), 8.58 (d, 1H, *J* = 5.2 Hz, quinoline proton), 9.23 (br s, 1H, -N*H*-quinoline), 9.72 (br s, 1H, –OCONH−), 10.08 (br s, 1H, −CON*H*-) ppm. ^13^C NMR (100 MHz, DMSO-*d*_6_) δ: 45.2 (2C), 63.1, 66.7, 105.5, 111.1, 116.1, 116.3, 118.0 (2C), 119.9, 123.3, 124.2, 125.5, 126.3, 128.0 (3C), 128.1, 128.3, 128.4 (2C), 130.1 (2C), 136.3, 138.9, 139.4, 139.6, 145.1, 145.6, 147.3, 148.0, 153.9, 166.2.

#### Benzyl (3-((Dimethylamino)­methyl)-5-(3-(quinolin-4-ylamino)­benzamido)­phenyl)­carbamate hydrochloride (**16**, MC3652)

Prepared starting from **39** ^19^ (0.47 mmol, 0.12 g) and **48** (0.47 mmol, 0.14 g). Mp: 233–235 °C (methanol); yield: 66%. ^1^H NMR (400 MHz, DMSO-*d*_6_) δ: 2.64 (s, 6H, –N­(CH_3_)_2_), 4.10 (s, 2H, –CH_2_N­(CH_3_)_2_), 5.18 (s, 2H, –CH_2_−), 6.98 (d, 1H, *J* = 5.2 Hz, quinoline proton), 7.32–7.42 (m, 6H, benzene and quinoline protons), 7.60–7.72 (m, 4H, benzene and quinoline protons), 7.92–8.04 (m, 5H, quinoline and benzene protons), 8.54 (s, 1H, quinoline proton), 8.68 (s, 1H, quinoline proton), 10.71 (br s, 1H, –OCONH−), 10.23 (br s, 1H, –NH–quinoline), 10.52 (br s, 1H, –CONH−) ppm. ^13^C NMR (100 MHz, DMSO-*d*_6_) δ: 45.2 (2C), 63.1, 66.7, 105.5, 111.2, 116.1, 116.3, 117.6, 119.9, 122.7, 123.1, 123.3, 125.5, 126.3, 128.0 (3C), 128.1, 128.3, 128.4, 128.5, 133.7, 136.3, 138.8, 139.2, 139.6, 142.0, 145.7, 147.3, 148.0, 153.9, 165.7.

#### *N*-(2-((Dimethylamino)­methyl)-4-(quinolin-4-ylamino)­phenyl)-4-(pyridin-4-ylamino)­benzamide hydrochloride (**17**, MC3683)

Prepared starting from **29** (0.47 mmol, 0.14 g) and **49** ^29^ (0.47 mmol, 0.11 g). Mp: 225–227 °C (methanol); yield: 72%. ^1^H NMR (400 MHz, DMSO-*d*_6_) δ: 2.77 (s, 6H, –N­(CH_3_)_2_), 4.37 (s, 2H, –CH_2_N­(CH_3_)_2_), 7.09 (d, 1H, *J* = 4.4 Hz, quinoline proton), 7.37 (s, 2H, benzene protons), 7.48 (d, 2H, *J* = 7.2 Hz, pyridine protons) 7.66 (s, 2H, benzene proton), 7.80–7.84 (m, 1H, quinoline proton), 7.94 (s, 1H, quinoline proton), 8.05–8.10 (m, 1H, benzene proton), 8.16–8.18 (m, 1H, quinoline proton), 8.30–8.37 (m, 4H, benzene and pyridine protons), 8.53 (d, 1H, *J* = 3.6 Hz, quinoline proton), 9.03 (d, 1H, *J* = 8.0 Hz, quinoline proton), 10.73 (br s, 1H, –NH–pyridine), 11.29 (bm, 1H, –CONH−), 11.43 (bm, 1H, NH-quinoline), 11.44 (bm, 1H, hydrochloride), 14.41 (br s, 1H, hydrochloride), 15.02 (br s, 1H, hydrochloride) ppm. ^13^C NMR (100 MHz, DMSO-*d*_6_) δ: 44.8 (2C), 60.8, 105.5, 110.2 (2C), 116.7 (2C), 119.6, 119.9, 120.1, 121.0, 123.3, 123.8, 125.5, 126.3, 126.9, 128.3, 130.0 (2C), 135.8, 136.8, 145.6, 146.9, 147.3, 147.9, 148.0, 148.6 (2C), 166.5.

#### *N*-(3-Amino-5-((dimethylamino)­methyl)­phenyl)-3-((2-amino-6-methylpyrimidin-4-yl)­amino)­benzamide (**38**)

Prepared starting from **37** ^19^ (0.47 mmol, 0.12 g) and **36a** (0.47 mmol, 0.08 g). Mp: 235–237 °C (methanol); yield: 67%. ^1^H NMR (400 MHz, CD_3_OD) δ: 2.21 (s, 3H, –CH_3_), 2.29 (s, 6H, N­(CH_3_)_2_), 3.41 (s, 2H, –CH_2_N­(CH_3_)_2_), 6.00 (s, 1H, pyrimidine proton), 6.11 (br s, 1H, –NH−), 6.52 (s, 1H, benzene proton), 6.90 (s, 1H, benzene proton), 7.17 (s, 1H, benzene proton), 7.42–7.46 (m, 1H, benzene proton), 7.53–7.56 (m, 1H, benzene proton), 7.77 (d, 1H, *J* = 7.6 Hz, benzene proton), 8.30 (s, 1H, benzene proton) ppm.

#### *N*-(3-Amino-5-((dimethylamino)­methyl)­phenyl)-3-(quinolin-4-ylamino)­benzamide (**40a**)

Prepared starting from **36a** (0.47 mmol, 0.08 g) and **39** ^**19**^ (0.47 mmol, 0.12 g). Mp: 200–202 °C (acetonitrile/methanol); yield: 75%. ^1^H NMR (400 MHz, DMSO-*d*_6_) δ: 2.18 (s, 6H, N­(CH_3_)_2_), 3.19 (s, 2H, –CH_2_N­(CH_3_)_2_), 5.05 (br s, 2H, –NH_2_), 6.43 (s, 1H, benzene proton), 6.95 (s, 1H, benzene proton), 7.14 (d 1H, *J* = 5.2 Hz, quinoline proton), 7.26 (s, 1H, benzene proton), 7.52–7.61 (m, 3H, benzene and quinoline protons), 7.70–7.78 (m, 3H, benzene and quinoline protons), 8.03 (s, 1H, quinoline proton), 8.36 (d, 1H, *J* = 7.6 Hz, quinoline proton), 8.57 (d, 1H, *J* = 4.8 Hz, quinoline proton), 9.30 (br s, 1H, –NH−), 10.38 (br s, 1H, –CONH−) ppm.

#### *N*-(3-Amino-5-((4-methylpiperazin-1-yl)­methyl)­phenyl)-3-(quinolin-4-ylamino)­benzamide (**40b**)

Prepared starting from **36b** (0.47 mmol, 0.10 g) and **39** ^19^ (0.47 mmol, 0.12 g). Mp: 126–129 °C (toluene); yield: 75%. ^1^H NMR (400 MHz, DMSO) δ: 2.14 (s, 3H, –CH_3_), 2.33 (s, 8H, piperazine protons), 3.26 (s, 2H, –CH_2_−), 5.08 (br s, 2H, –NH_2_), 6.29 (s, 1H, benzene proton), 6.81 (s, 1H, benzene proton), 7.52–7.60 (m, 3H, benzene and quinoline protons), 7.69–7.76 (m, 2H, benzene and quinoline protons), 7.90–7.92 (m, 2H, benzene and quinoline protons), 8.41 (d, 1H, *J* = 8.4 Hz, quinoline proton), 8.51 (d, 1H, *J* = 4.0 Hz, quinoline proton), 9.14 (br s, 1H, –NH−), 9.97 (br s, 1H, –CONH−) ppm.

#### *N*-(3-((1H-Imidazole-1-yl)­methyl)-5-aminophenyl)-3-(quinolin-4-ylamino)­benzamide (**40c**)

Prepared starting from **36c** (0.47 mmol, 0.09 g) and **39** ^19^ (0.47 mmol, 0.12 g). Mp: 130–132 °C (toluene); yield: 81%. ^1^H NMR (400 MHz, DMSO-*d*_6_) δ: 5.04 (s, 2H, –CH_2_−), 5.12 (br s, 2H, –NH_2_), 6.15 (s, 1H, benzene proton), 6.77 (s, 1H, benzene proton), 7.04 (s, 2h, benzene protons), 7.55–7.50 (m, 4H, benzene, imidazole and quinoline protons), 7.69–7.77 (m, 3H, benzene, imidazole and quinoline protons), 7.91–7.96 (m, 3H, benzene, imidazole and quinoline protons), 8.43 (d, 1H, *J* = 7.6 Hz, quinoline proton), 8.52–8.57 (m, 1H, quinoline proton), 9.28 (br s, 1H, –NH−), 10.03 (br s, 1H, –CONH−) ppm.

#### *N*-(3-Amino-5-((dimethylamino)­methyl)­phenyl)-3-((dimethylamino)­methyl)-5-(quinolin-4-ylamino)­benzamide (**41**)

Prepared starting from **36a** (0.47 mmol, 0.08 g) and **25b** (0.47 mmol, 0.15 g). Mp: 220–222 °C (acetonitrile/methanol); yield: 65%. ^1^H NMR (400 MHz, DMSO-*d*_6_) δ: 2.15 (s, 6H, N­(CH_3_)_2_), 2.21 (s, 6H, N­(CH_3_)_2_), 3.22 (s, 2H, –CH_2_N­(CH_3_)_2_), 3.49 (s, 2H, –CH_2_N­(CH_3_)_2_), 5.06 (br s, 2H, –NH_2_), 6.29 (s, 1H, benzene proton), 6.82 (s, 1H, benzene proton), 7.03–7.05 (m, 2H, benzene and quinoline protons), 7.47–7.50 (m, 1H, benzene proton), 7.54–7.57 (m, 1H, quinoline proton),7.62 (s, 1H, benzene proton), 7.70–7.74 (m, 1H, quinoline proton), 7.83 (s, 1H, benzene proton), 7.90 (d, 1H, *J* = 8.8 Hz, quinoline proton), 8.42 (d, 1H, *J* = 7.6 Hz, quinoline proton), 8.51 (d, 1H, *J* = 4.8 Hz, quinoline proton), 9.12 (br s, 1H, –NH−), 9.93 (br s, 1H, –CONH−) ppm.

#### *N*-(3-Amino-5-(dimethylamino)­phenyl)-3-(quinolin-4-ylamino)­benzamide (**43**)

Prepared starting from **39** ^19^ (0.47 mmol, 0.12 g) and **42** (0.47 mmol, 0.07 g). Mp: 115–117 °C (toluene); yield: 72%. ^1^H NMR (400 MHz, DMSO-*d*_6_) δ: 2.81 (s, 6H, 2x-CH_3_), 4.86 (br s, 2H, –NH_2_), 5.78 (s, 1H, benzene proton), 6.39 (s, 1H, benzene proton), 6.59 (s, 1H, benzene proton), 7.03 (d, 1H, *J* = 5.6 Hz, benzene proton), 7.52–7.58 (m, 3H, benzene and quinoline protons), 7.67–7.77 (m, 2H, benzene and quinoline protons), 7.90–7.92 (m, 2H, benzene and quinoline protons), 8.41 (d, 1H, *J* = 9.2 Hz, quinoline proton), 8.51 (d, 1H, *J* = 4.8 Hz, quinoline proton), 9.12 (br s, 1H, –NH−), 9.79 (br s, 1H, –CONH−) ppm.

#### *N*-(3-Amino-5-((dimethylamino)­methyl)­phenyl)-4-(quinolin-4-ylamino)­benzamide (**50**)

Prepared starting from **33** ^19^ (0.47 mmol, 0.12 g) and **36a** (0.47 mmol, 0.08 g). Mp: 181–183 °C (acetonitrile/methanol); yield: 80%. ^1^H NMR (400 MHz, DMSO-*d*_6_) δ: 2.15 (s, 6H, N­(CH_3_)_2_), 3.18 (s, 2H, –CH_2_N­(CH_3_)_2_), 5.04 (br s, 2H, –NH_2_), 6.28 (s, 1H, benzene proton), 6.85 (s, 1H, benzene proton), 7.05 (t, 1H, *J* = 2.0 Hz, benzene proton), 7.20 (d, 1H, *J* = 5.2 Hz, quinoline proton), 7.46 (d, 2H, *J* = 8.8 Hz, benzene proton), 7.60 (t, 1H, *J* = 2.0 Hz, benzene proton), 7.74 (t, 1H, *J* = 2.0 Hz, benzene proton), 7.93 (d, 1H, *J* = 8.4 Hz, benzene proton), 7.98 (d, 2H, *J* = 8.8 Hz, quinoline protons), 8.38 (d, 1H, *J* = 7.6 Hz, quinoline proton), 8.58 (d, 1H, *J* = 5.2 Hz, quinoline proton), 9.21 (br s, 1H, –NH−), 9.82 (br s, 1H, –CONH−) ppm.

#### *N*-(3-Amino-5-((dimethylamino)­methyl)­phenyl)-4-((2-amino-6-methylpyrimidin-4-yl)­amino)­benzamide (**51**)

Prepared starting from **30** ^19^ (0.47 mmol, 0.11 g) and **36a** (0.47 mmol, 0.08 g). mp: >250 °C (methanol); yield: 70%. ^1^H NMR (400 MHz, CD_3_OD) δ: 2.25 (s, 3H, −C*H*_3_), 2.33 (s, 6H, N­(CH_3_)_2_), 3.45 (s, 2H, –CH_2_N­(CH_3_)_2_), 6.04 (s, 1H, pyrimidine proton), 6.12 (br s, 1H, –NH−), 6.51 (s, 1H, benzene proton), 6.89 (s, 1H, benzene proton), 7.15 (s, 1H, benzene proton), 7.87–7.88 (m, 4H, benzene protons) ppm.

#### General Procedure for the Synthesis of the Final Compounds **8–14**, **17–21** and of the Amine Intermediate Compounds **24a**,**b**, **28**, and **31**

A mixture containing either 4-chloroquinoline (0.39 mmol, 1 equiv) or 2-amino-4-chloro-6-methylpyrimidine (0.39 mmol, 1 equiv), the appropriate amino derivative (0.39 mmol, 1 equiv) and catalytic amount (4 drops) of 37% HCl in ethanol (30 mL) was refluxed for 2 h. Then, the mixture was allowed to cool down to rt, and the precipitated solid was filtered off, washed with water (3 × 5 mL), and purified by column chromatography (SiO_2_ eluting with chloroform/methanol/NH_3_:20/1/0.1) to afford the pure product.

#### 3-((2-Amino-6-methylpyrimidin-4-yl)­amino)-*N*-(3-((dimethylamino)­methyl)-5-(quinolin-4-ylamino)­phenyl)­benzamide hydrochloride (**8**, MC3653)

Prepared starting from 4-chloroquinoline (0.39 mmol, 0.06 g) and **38** (0.39 mmol, 0.15 g). Mp: 219–221 °C (acetonitrile/methanol); yield: 73%. ^1^H NMR (400 MHz, DMSO-*d*_6_) δ: 2.29 (s, 3H, –CH_3_), 2.77 (s, 6H, –N­(CH_3_)_2_), 4.35 (s, 2H, –CH_2_N­(CH_3_)_2_), 6.27 (s, 1H, pyrimidine proton), 7.14 (d, 1H, *J* = 5.6 Hz, quinoline proton), 7.53 (s, 2H, benzene protons), 7.78–7.83 (m, 2H, benzene protons),7.97 (s, 1H, benzene proton), 8.05–8.20 (m, 5H, benzene and quinoline protons), 8.58 (d, 1H, *J* = 6.4 Hz, quinoline proton), 8.90 (d, 1H, *J* = 8.4 Hz, quinoline proton), 10.82 (br s, 1H, –NH– pyrimidine quinoline), 10.95 (br s, 1H, –NH– quinoline), 11.20 (br s, 1H, –CONH−) ppm. ^13^C NMR (100 MHz, DMSO-*d*_6_) δ: 23.8, 45.2 (2C), 63.2, 97.4, 105.6, 110.8, 115.3, 116.2, 117.5, 119.9, 122.2, 122.6, 123.3, 125.5, 126.3, 128.3, 128.6, 134.1, 138.9, 139.8, 141.3, 143.1, 145.7, 147.3, 148.0, 158.1, 162.4, 165.6, 166.0.

#### *N*-(3-((2-Amino-6-methylpyrimidin-4-yl)­amino)-5-(dimethylamino)­phenyl)-3-(quinolin-4-ylamino)­benzamide hydrochloride (**9**, MC3807)

Prepared starting from 2-amino-4-chloro-6-methylpyrimidine (0.39 mmol, 0.06 g) and **43** (0.39 mmol, 0.15 g). Mp: >250 °C (methanol); yield: 59%. ^1^H NMR (400 MHz, DMSO-*d*_6_) δ: 2.29 (s, 3H, –CH_3_), 2.99 (s, 6H, –N­(CH_3_)_2_), 6.21 (s, 1H, pyrimidine proton), 6.94 (d, 1H, *J* = 6.8 Hz, quinoline proton), 7.27–7.36 (br s, 1H, benzene proton), 7.74–7.78 (m, 3H, benzene and quinoline protons), 7.87 (t, 1H, *J* = 8.0 Hz, quinoline proton), 8.05–8.14 (m, 5H, benzene and quinoline protons), 8.56–8.58 (m, 1H, quinoline proton), 8.98 (d, 1H, *J* = 8.4 Hz, quinoline proton), 10.48 (br s, 1H, –NH–pyrimidine) 10.65 (br s, 1H, NH- quinoline), 11.19 (br s, 1H, –CONH−), 12.84 (br s, 1H, hydrochloride), 14.74 (br s, 1H, hydrochloride) ppm. ^13^C NMR (100 MHz, DMSO-*d*_6_) δ: 23.8, 41.4 (2C), 97.4, 101.8, 102.9, 105.5, 107.1, 117.6, 119.9, 122.7, 123.1, 123.3, 125.5, 126.3, 128.3, 128.5, 133.6, 141.5, 142.0, 143.8, 145.7, 147.3, 148.0, 153.3, 157.8, 162.4, 165.7, 166.0. HPLC: 98.2% pure (retention time 12.51 min) (Supporting Information Figure S2).

#### *N*-(3-((2-Amino-6-methylpyrimidin-4-yl)­amino)-5-((dimethylamino)­methyl)­phenyl)-3- (quinolin-4-ylamino)­benzamide (**10**, MC3563)

Prepared starting from 2-amino-4-chloro-6-methylpyrimidine (0.39 mmol, 0.06 g) and **40a** (0.39 mmol, 0.16 g). Mp: 195–197 °C (acetonitrile/methanol); yield: 68%. ^1^H NMR (400 MHz, DMSO-*d*_6_) δ: 2.09 (s, 3H, –CH_3_), 2.18 (s, 6H, –N­(CH_3_)_2_), 3.35 (s, 2H, –CH_2_N­(CH_3_)_2_), 5.92 (s, 1H, pyrimidine proton), 6.02 (br s, 2H, –NH_2_-pyrimidine), 7.06–7.07 (d, 1H, *J* = 7.6 Hz, quinoline proton), 7.34 (s, 1H, benzene proton), 7.38 (s, 1H, quinoline proton), 7.55–7.58 (m, 3H, benzene and quinoline protons), 7.71–7.74 (m, 2H, benzene protons), 7.91 (d, 1H, *J* = 8.4 Hz, quinoline proton), 7.96 (s, 1H, benzene proton), 8.04 (s, 1H, benzene proton), 8.41 (d, 1H, *J* = 8.0 Hz, quinoline proton), 8.52 (d, 1H, *J* = 5.2 Hz, quinoline proton), 8.99 (br s, 1H, –NH– pyrimidine), 9.15 (br s, 1H, –NH–quinoline), 10.14 (br s, 1H, –CONH−) ppm. ^13^C NMR (100 MHz, DMSO-*d*_6_) δ: 23.8, 45.2 (2C), 63.2, 97.4, 105.5, 110.7, 115.2, 116.2, 117.6, 119.9, 122.7, 123.1, 123.3, 125.5, 126.3, 128.3, 128.5, 133.7, 138.9, 139.6, 142.0, 142.3, 145.7, 147.3, 148.0, 157.9, 162.4, 165.7, 166.0. HPLC: 97.9% pure (retention time 12.12 min) (Supporting Information Figure S3).

#### N^4^-(3-((dimethylamino)­methyl)-5-((3-(quinolin-4-ylamino)­benzyl)­amino)­phenyl)-6-methylpyrimidine-2,4-diamine (**11**, MC3718)

Prepared starting from 2-amino-4-chloro-6-methylpyrimidine (0.39 mmol, 0.06 g) and **47** (0.39 mmol, 0.15 g). mp: >250 °C (methanol); yield: 60%. ^1^H NMR (400 MHz, DMSO-*d*_6_) δ: 2.30 (s, 3H, –CH_3_), 2.75 (s, 6H, –N­(CH_3_)_2_), 2.82 (s, 6H, –N­(CH_3_)_2_), 4.33 (s, 2H, –CH_2_N­(CH_3_)_2_), 4.48 (s, 2H, –CH_2_N­(CH_3_)_2_), 6.33 (s, 1H, pyrimidine proton), 7.21 (d, 1H, *J* = 6.8 Hz, quinoline proton), 7.80–7.87 (m, 2H, benzene and quinoline proton), 8.00–8.09 (m, 3H, benzene and quinoline protons), 8.15–8.19 (m, 2H, benzene and quinoline proton), 8.39 (s, 2H, benzene protons), 8.58 (d, 1H, *J* = 6.0 Hz, quinoline proton), 8.98 (d, 1H, *J* = 8.4 Hz, quinoline proton), 10.75 (br s, 1H, –NH–pyrimidine) 10.86 (br s, 1H, NH- quinoline), 10.97 (br s, 1H, hydrochloride), 11.14 (br s, 1H, hydrochloride), 11.37 (br s, 1H, –CONH−), 12.93 (br s, 1H, hydrochloride), 14.90 (br s, 1H, hydrochloride) ppm. ^13^C NMR (100 MHz, DMSO-*d*_6_) δ: 23.8, 45.2 (3C), 45.3, 63.2, 63.3, 97.4, 105.5, 110.7, 115.2, 116.2, 116.8, 119.9, 120.4, 123.3, 125.5, 125.5, 126.3, 128.3, 133.2, 138.8, 139.3, 139.5, 141.8, 142.3, 145.7, 147.3, 148.0, 157.9, 162.4, 165.9, 166.0.

#### *N*-(3-((2-Amino-6-methylpyrimidin-4-yl)­amino)-5-((4-methylpiperazin-1-yl)­methyl) phenyl)-3-(quinolin-4-ylamino)­benzamide (**12**, MC3669)

Prepared starting from 2-amino-4-chloro-6-methylpyrimidine (0.39 mmol, 0.06 g) and **40b** (0.39 mmol, 0.18 g). Mp: 243–245 °C (methanol); yield: 67%. ^1^H NMR (400 MHz, DMSO-*d*_6_) δ: 2.09 (s, 3H, –CH_3_, pyrimidine), 2.16 (s, 2H, –CH_3_ piperazine), 2.34–2.41 (m, 8H, piperazine protons), 3.43 (s, 2H, –CH_2_), 5.92 (s, 1H, pyrimidine proton), 6.01 (br s, 2H, –NH_2_), 7.06 (d, 1H, *J* = 4.8 Hz, quinoline proton), 7.35 (s, 2H, benzene protons), 7.55–7.58 (m, 3H, benzene and quinoline protons), 7.71–7.74 (m, 2H, benzene and quinoline protons), 7.90–7.96 (m, 2H, benzene protons), 8.04 (s, 1H, quinoline protons), 8.42 (d, 1H, *J* = 5.4 Hz, quinoline proton), 8.52 (d, 1H, *J* = 5.2 Hz, quinoline proton), 8.99 (br s, 1H, –NH– pyrimidine), 9.14 (br s, 1H, –NH– quinoline), 10.14 (s, 1H, –CONH−) ppm. ^13^C NMR (100 MHz, DMSO-*d*_6_) δ: 23.8, 45.4, 53.0 (2C), 54.7 (2C), 59.4, 97.4, 105.5, 110.9, 115.6, 116.5, 117.6, 119.9, 122.7, 123.1, 123.3, 125.5, 126.3, 128.3, 128.5, 133.7, 138.2, 139.6, 142.0, 142.3, 145.7, 147.3, 148.0, 157.9, 162.4, 165.7, 166.0. HPLC: 96.4% pure (retention time 11.96 min) (Supporting Information Figure S4).

#### *N*-(3-((1H-Imidazole-1-yl)­methyl)-5-((2-amino-6-methylpyrimidin-4-yl)­amino)­phenyl)-3-(quinolin-4-ylamino)­benzamide hydrochloride (**13**, MC3821)

Prepared starting from 2-amino-4-chloro-6-methylpyrimidine (0.39 mmol, 0.06 g) and **40c** (0.39 mmol, 0.17 g). Mp: >250 °C (methanol); yield: 61%. ^1^H NMR (400 MHz, DMSO-*d*_6_) δ: 2.29 (s, 3H, –CH_3_), 5.55 (s, 2H, –CH_2_–imidazole), 6.29 (s, 1H, pyrimidine proton), 6.95 (d, 1H, *J* = 6.8 Hz, quinoline proton), 7.69–7.86 (m, 8H, benzene imidazole, and quinoline protons), 8.03–8.16 (m, 4H, benzene and quinoline protons), 8.30 (s, 1H, imidazole proton), 8.55 (d, 1H, *J* = 7.6 Hz, quinoline proton), 8.96 (d, 1H, *J* = 8.4 Hz, quinoline proton), 9.49 (s, 1H, imidazole proton), 10.79 (br s, 1H, –NH–pyrimidine) 10.97 (br s, 1H, NH- quinoline), 11.30 (br s, 1H, –CONH−), 13.05 (br s, 1H, hydrochloride), 14.91 (br s, 1H, hydrochloride) ppm. ^13^C NMR (100 MHz, DMSO-*d*_6_) δ: 23.8, 49.0, 97.4, 105.5, 110.9, 116.2, 117.1, 117.6, 119.9, 121.0, 122.7, 123.1, 123.3, 125.5, 126.3, 128.3, 128.5, 129.1, 133.7, 136.6, 137.3, 139.8, 142.0, 142.0, 145.7, 147.3, 148.0, 157.9, 162.4, 165.7, 166.0. HPLC: 99.4% pure (retention time 12.17 min) (Supporting Information Figure S5).

#### *N*-(3-((2-Amino-6-methylpyrimidin-4-yl)­amino)-5-((dimethylamino)­methyl)­phenyl)-3-((dimethylamino)­methyl)-5-(quinolin-4-ylamino)­benzamide hydrochloride (**14**, MC3817)

Prepared starting from 2-amino-4-chloro-6-methylpyrimidine (0.39 mmol, 0.06 g) and **41** (0.39 mmol, 0.18 g). Mp: >250 °C (methanol); yield: 60%. ^1^H NMR (400 MHz, DMSO-*d*_6_) δ: 2.30 (s, 3H, –CH_3_), 2.75 (s, 6H, –N­(CH_3_)_2_), 2.82 (s, 6H, –N­(CH_3_)_2_), 4.33 (s, 2H, –CH_2_N­(CH_3_)_2_), 4.48 (s, 2H, –CH_2_N­(CH_3_)_2_), 6.33 (s, 1H, pyrimidine proton), 7.21 (d, 1H, *J* = 6.8 Hz, quinoline proton), 7.80–7.87 (m, 2H, benzene and quinoline proton), 8.00–8.09 (m, 3H, benzene and quinoline protons), 8.15–8.19 (m, 2H, benzene and quinoline proton), 8.39 (s, 2H, benzene protons), 8.58 (d, 1H, *J* = 6.0 Hz, quinoline proton), 8.98 (d, 1H, *J* = 8.4 Hz, quinoline proton), 10.75 (br s, 1H, –NH–pyrimidine) 10.86 (br s, 1H, NH- quinoline), 10.97 (br s, 1H, hydrochloride), 11.14 (br s, 1H, hydrochloride), 11.37 (br s, 1H, –CONH−), 12.93 (br s, 1H, hydrochloride), 14.90 (br s, 1H, hydrochloride) ppm. ^13^C NMR (100 MHz, DMSO-*d*_6_) δ: 23.8, 45.2 (3C), 45.3, 63.2, 63.3, 97.4, 105.5, 110.7, 115.2, 116.2, 116.8, 119.9, 120.4, 123.3, 125.5, 125.5, 126.3, 128.3, 133.2, 138.8, 139.3, 139.5, 141.8, 142.3, 145.7, 147.3, 148.0, 157.9, 162.4, 165.9, 166.0. HPLC: 97.6% pure (retention time 11.30 min) (Supporting Information Figure S6).

#### *N*-(3-((Dimethylamino)­methyl)-5-(quinolin-4-ylamino)­phenyl)-4-(quinolin-4-ylamino) benzamide hydrochloride (**18**, MC3646)

Prepared starting from 4-chloroquinoline (0.39 mmol, 0.06 g) and **50** (0.39 mmol, 0.16 g). Mp: >250 °C (methanol); yield: 71%. ^1^H NMR (400 MHz, DMSO-*d*_6_) δ: 2.80 (s, 6H, –N­(CH_3_)_2_), 4.37 (s, 2H, –CH_2_N­(CH_3_)_2_), 7.03 (d, 1H, *J* = 6.8 Hz, quinoline proton), 7.15 (d, 1H, *J* = 6.8 Hz, quinoline proton), 7.55 (s, 1H, benzene proton), 7.71 (d, 2H, *J* = 8.4 Hz, benzene protons), 7.84–7.88 (m, 2H, benzene protons), 8.03–8.12 (m, 6H, quinoline and benzene protons), 8.21 (d, 2H, *J* = 8.4 Hz, benzene protons), 8.59–8.64 (m, 2H, quinoline protons), 8.83–8.89 (m, 2H, quinoline protons), 10.86 (br s, 2H, −CONH- and –NH–quinoline), 11.12 (br s, 2H, –NH– quinoline and hydrochloride), 14.66 (br s, 2H, 2x hydrochloride) ppm. ^13^C NMR (100 MHz, DMSO-*d*_6_) δ: 45.2 (2C), 63.2, 105.5, 105.6, 110.9, 115.3, 116.2, 118.0 (2C), 119.9 (2C), 123.3, 123.3, 124.2, 125.5, 125.6, 126.3, 126.4, 128.3, 128.4, 130.1 (2C), 138.6, 139.8, 143.1, 145.1, 145.6, 145.7, 147.3, 147.3, 148.0, 148.0, 166.2.

#### *N*-(3-((Dimethylamino)­methyl)-5-(quinolin-4-ylamino)­phenyl)-3-(quinolin-4-ylamino)­benzamide hydrochloride (**19**, MC3639)

Prepared starting from 4-chloroquinoline (0.39 mmol, 0.06 g) and **40a** (0.39 mmol, 0.16 g). Mp: >250 °C (methanol); yield: 73%. ^1^H NMR (400 MHz, DMSO-*d*_6_) δ: 2.20 (s, 6H, –N­(CH_3_)_2_), 3.41 (s, 2H, –CH_2_N­(CH_3_)_2_), 7.03–7.06 (m, 3H, benzene and quinoline protons), 7.51–7.59 (m, 5H, benzene and quinoline protons), 7.68–7.75 (m, 3H, benzene and quinoline protons), 7.87–7.92 (m, 3H, benzene and quinoline protons), 7.99 (s, 1H, benzene proton), 8.40–8.43 (m, 2H, quinoline protons), 8.48–8.52 (m, 2H, quinoline protons) 9.02 (br s, 1H, –NH– quinoline), 9.16 (br s, 1H, –NH–quinoline), 10.33 (br s, 1H, –CONH−) ppm. ^13^C NMR (100 MHz, CD_3_OD) δ: 45.3 (2C), 63.7, 105.3, 105.4, 110.5, 115.4, 116.4, 117.6, 119.9, 120.0, 122.6, 123.0, 123.0, 123.2, 125.9, 125.9, 126.3, 126.4, 128.3, 128.3, 128.6, 133.9, 139.0, 139.9, 142.1, 143.1, 145.9, 146.2, 147.2, 147.2, 147.4, 147.4, 166.3.

#### 4-((2-Amino-6-methylpyrimidin-4-yl)­amino)-*N*-(3-((2-amino-6-methylpyrimidin-4-yl) amino)-5-((dimethylamino)­methyl)­phenyl)­benzamide hydrochloride (**20**, MC3647)

Prepared starting from 2-amino-4-chloro-6-methylpyrimidine (0.39 mmol, 0.06 g) and **51** (0.39 mmol, 0.15 g). Mp: 149–151 °C (acetonitrile); yield: 65%. ^1^H NMR (400 MHz, CD_3_OD) δ: 2.20 (s, 6H, 2x-CH_3_), 2.36 (s, 6H, –N­(CH_3_)_2_), 3.55 (s, 2H, –CH_2_N­(CH_3_)_2_), 6.02 (s, 1H, pyrimidine proton), 6.04 (s, 1H, pyrimidine proton), 7.28 (s, 1H, benzene proton), 7.39 (s, 1H, benzene proton), 7.86–7.94 (m, 4H, benzene protons), 8.12 (br s, 1H, –CONH−), ppm. ^13^C NMR (100 MHz, DMSO-*d*_6_) δ: 23.8 (2C), 45.2 (2C), 63.2, 97.4, 97.5, 110.7, 115.2, 116.2, 117.5 (2C), 124.2, 129.9, 138.9, 139.5, 142.3, 143.9, 158.0, 158.2, 162.4, 162.4, 166.0 (2C), 166.1, 166.2.

#### 3-((2-Amino-6-methylpyrimidin-4-yl)­amino)-*N*-(3-((2-amino-6-methylpyrimidin-4-yl) amino)-5-((dimethylamino)­methyl)­phenyl)­benzamide hydrochloride (**21**, MC3649)

Prepared starting from 2-amino-4-chloro-6-methylpyrimidine (0.39 mmol, 0.06 g) and **38** (0.39 mmol, 0.15 g). Mp: >250 °C (methanol); yield: 69%. ^1^H NMR (400 MHz, DMSO-*d*_6_) δ: 2.30 (s, 6H, 2x-CH_3_), 2.76 (s, 6H, –N­(CH_3_)_2_), 4.33 (s, 2H, –CH_2_N­(CH_3_)_2_), 6.29 (s, 2H, pyrimidine protons), 7.52–7.56 (m, 2H, benzene protons), 7.72–7.80 (m, 3H, benzene protons), 8.07–8.20 (m, 4H, benzene protons), 10.68 (br s, 1H, –NH– pyrimidine), 10.77 (br s, 1H, –NH– pyrimidine), 10.91 (br s, 1H, –CONH−), 10.99 (bds, 1H, hydrochloride), 12.92 (br s, 2H, 2x hydrochloride) ppm. ^13^C NMR (100 MHz, DMSO-*d*_6_) δ: 23.8 (2C), 45.2 (2C), 63.2, 97.4, 97.4, 110.7, 115.2, 116.2, 117.5, 122.2, 122.6, 128.6, 134.1, 138.9, 139.6, 141.3, 142.3, 158.0, 158.1, 162.4, 162.4 (2C), 165.6, 166.0 (2C), 166.1.

#### Methyl 2-((dimethylamino)­methyl)-4-(quinolin-4-ylamino)­benzoate (**24a**)

Prepared starting from 4-chloroquinoline (0.39 mmol, 0.06 g) and **23a** (0.39 mmol, 0.08 g). Mp: >250 °C (methanol); yield: 75%. ^1^H NMR (400 MHz, CDCl_3_) δ: 2.30 (s, 6H, –N­(CH_3_)_2_), 3.83 (s, 2H, –CH_2_N­(CH_3_)_2_), 3.91 (s, 3H, –CH_3_), 7.02 (br s, 1H, –NH−), 7.23–7.25 (m, 2H, benzene protons), 7.46 (s, 1H, quinoline proton), 7.52–7.55 (m, 1H, quinoline proton), 7.70–7.74 (m, 1H, benzene proton), 7.93 (d, 1H, *J* = 8.4 Hz, quinoline proton), 7.99 (d, 1H, *J* = 8.4 Hz, quinoline proton), 8.09 (d, 1H, *J* = 4.4 Hz, quinoline proton), 8.67 (d, 1H, *J* = 4.8 Hz, quinoline proton) ppm.

#### Methyl 3-((dimethylamino)­methyl)-5-(quinolin-4-ylamino)­benzoate (**24b**)

Prepared starting from 4-chloroquinoline (0.39 mmol, 0.06 g) and **23b** (0.39 mmol, 0.08 g). Mp: 99–101 °C (toluene); yield: 78%. ^1^H NMR (400 MHz, CDCl_3_) δ: 2.30 (s, 6H, –N­(CH_3_)_2_), 3.51 (s, 3H, –CH_3_), 3.95 (s, 2H, –CH_2_N­(CH_3_)_2_), 6.71 (br s, 1H, –NH−), 7.04–7.07 (m, 1H, benzene proton), 7.51–53 (m, 1H, benzene proton), 7.54–7.58 (m, 1H, quinoline proton), 7.72–7.79 (m, 2H, benzene and quinoline protons), 7.92–7.95 (m, 2H, quinoline protons), 8.10 (d, 1H, *J* = 8.4 Hz, quinoline proton), 8.65 (d, 1H, *J* = 5.2 Hz, quinoline proton) ppm.

#### *N*-(3-((Dimethylamino)­methyl)-4-nitrophenyl)­quinolin-4-amine (**28**)

Prepared starting from 4-chloroquinoline (0.39 mmol, 0.06 g) and **27** (0.39 mmol, 0.08 g). Mp: >250 °C (methanol); yield: 74%. ^1^H NMR (400 MHz, CD_3_OD) δ: 2.27 (s, 6H, –N­(CH_3_)_2_), 3.79 (s, 2H, –CH_2_N­(CH_3_)_2_), 7.33 (s, 1H, benzene proton), 7.38 (d, 1H, *J* = 8.4 Hz, quinoline proton), 7.51 (s, 1H, benzene proton), 7.54–7.61 (m, 1H, quinoline proton), 7.71–7.78 (m, 1H, quinoline proton), 7.94 (d, 1H, *J* = 8.4 Hz, benzene proton), 8.04 (d, 1H, *J* = 8.4 Hz, quinoline proton), 8.25 (d, 1H, *J* = 7.6 Hz, quinoline proton), 8.56 (s, 1H, quinoline proton) ppm.

#### *N* ^4^-(3-((Dimethylamino)­methyl)-4-nitrophenyl)-6-methylpyrimidine-2,4-diamine (**31**)

Prepared starting from 2-amino-4-chloro-6-methylpyrimidine (0.39 mmol, 0.06 g) and **27** (0.39 mmol, 0.08 g). Mp: >250 °C (methanol); yield: 70%. ^1^H NMR (400 MHz, CDCl_3_) δ: 1.65 (s, 3H, –CH_3_), 2.30 (s, 2H, –CH_2_N­(CH_3_)_2_), 3.82 (s, 2H, –CH_2_N­(CH_3_)_2_), 4.91 (br s, 2H, NH_2_), 6.04 (s, 1H, pyrimidine proton), 7.73 (br s, 1H, –NH−), 7.73–7.65 (m, 1H, benzene proton), 8.04 (d, 1H, *J* = 9.2 Hz, benzene proton) ppm.

#### General Procedure for the Synthesis of the Amine Intermediate Compounds **22a,b**, **35a–c**

The appropriate alkyl bromide (0.75 mmol, 1 equiv) was dissolved in dry THF and added with the appropriate amine (2.25 mmol, 3 equiv). After 5 h, the reaction was quenched at rt with water (20 mL), and the mixture was extracted with ethyl acetate (3 × 30 mL) and washed with brine (3 × 30 mL). The combined organic extracts were dried over sodium sulfate, and upon solvent evaporation, the amine product was used without further purification.

#### Methyl 2-((Dimethylamino)­methyl)-4-nitrobenzoate (**22a**)

Prepared starting from methyl 2-(bromomethyl)-4-nitrobenzoate (0.75 mmol, 0.20 g) and dimethylamine (2.25 mmol, 0.10 g). Mp: 110–112 °C (toluene); yield: 69%. ^1^H NMR (400 MHz, CDCl_3_) δ: 2.35 (s, 6H, –N­(CH_3_)_2_), 3.84 (s, 3H, –CH_3_), 4.00 (s, 2H, –CH_2_N­(CH_3_)_2_), 8.22–8.25 (m, 2H, benzene protons), 8.33–8.35 (m, 1H, benzene proton) ppm.

#### Methyl 3-((Dimethylamino)­methyl)-5-nitrobenzoate (**22b**)

Prepared starting from methyl 3-(bromomethyl)-5-nitrobenzoate (0.75 mmol, 0.20 g) and dimethylamine (2.25 mmol, 0.10 g). Mp: 134–136 °C (toluene); yield: 63%. ^1^H NMR (400 MHz, CDCl_3_) δ: 2.30 (s, 6H, –N­(CH_3_)_2_), 3.58 (s, 2H, –CH_2_N­(CH_3_)_2_), 4.00 (s, 3H, –CH_3_), 8.33–8.35 (m, 1H, benzene proton), 8.41–8.43 (m, 1H, benzene proton), 8.76–8.78 (m, 1H, benzene proton) ppm.

#### Di*tert*-butyl (5-((Dimethylamino)­methyl)-1,3-phenylene)­dicarbamate (**35a**)

Prepared starting from **34** (0.75 mmol, 0.30 g) and dimethylamine (2.25 mmol, 0.10 g). Mp: 197–199 °C (acetonitrile/methanol); yield: 77%. ^1^H NMR (400 MHz, CDCl_3_) δ: 1.43 (s, 18H, 2x C­(CH_3_)_3_), 2.16 (s, 6H, –N­(CH_3_)_2_), 3.29 (s, 2H, –CH_2_N­(CH_3_)_2_), 6.53 (br s, 2H, –OCONH−), 6.93 (s, 2H, benzene protons), 7.36 (s, 1H, benzene proton) ppm.

#### Di*tert*-butyl (5-((4-Methylpiperazin-1-yl)­methyl)-1,3-phenylene)­dicarbamate (**35b**)

Prepared starting from **34** (0.75 mmol, 0.30 g) and *N*-methylpiperazine (2.25 mmol, 0.22 g). Mp: 220–222 °C (acetonitrile/methanol); yield: 71%. ^1^H NMR (400 MHz, DMSO-*d*_6_) δ: 1.47 (s, 18H, C­(CH_3_)_3_), 2.70 (s, 3H, –CH_3_), 3.33 (s, 8H, piperazine protons), 3.40 (s, 2H, –CH_2_−), 7.05 (s, 2H, benzene protons) 7.48 (s, 1H, benzene proton), 9.27 (br s, 2H, 2x–OCONH−) ppm.

#### Di*tert*-butyl (5-((1H-Imidazole-1-yl)­methyl)-1,3-phenylene)­dicarbamate (**35c**)

Prepared starting from **34** (0.75 mmol, 0.30 g) and imidazole (2.25 mmol, 0.15 g). Mp: 226–228 °C (acetonitrile/methanol); yield: 73%. ^1^H NMR (400 MHz, CDCl_3_) δ: 1.51 (s, 18H, 2x C­(CH_3_)_3_), 5.06 (s, 2H, –CH_2_−), 6.64 (br s, 2H, 2x–OCONH−), 6.88 (s, 2H, benzene protons), 6.92 (s, 1H, imidazole proton), 7.10 (s, 1H, imidazole proton), 7.44 (s, 1H, imidazole proton), 7.55 (s, 1H, benzene proton) ppm.

#### General Procedure for the Synthesis of the Amine Intermediate Compounds **23a,b**, **29**, **32**, **42**

A solution of 37% HCl (0.2 mL) was slowly added at 0 °C to a cooled solution of the appropriate nitro derivative (0.03 mmol, 1 equiv) and stannous chloride dihydrate (0.15 mmol, 0.03 g, 5 equiv) in ethanol (5 mL). The reaction was then kept at 80 °C for 1 h. Afterward, the reaction was quenched at rt by 2N sodium carbonate solution (20 mL), and the mixture was extracted with ethyl acetate (3 × 30 mL) and washed with brine (3 × 30 mL). The combined organic extracts were dried over sodium sulfate, and upon solvent evaporation, the residue was purified by column chromatography (SiO_2_ eluting with ethyl acetate/methanol/NH_3_:10/1/0.1) to afford the desired amine product.

#### Methyl 4-Amino-2-((dimethylamino)­methyl)­benzoate (**23a**)

Prepared starting from **22a** (0.03 mmol, 0.007 g). Mp: 150–153 °C (acetonitrile); yield: 78%. ^1^H NMR (400 MHz, DMSO-*d*_6_) δ: 2.76 (s, 6H, –N­(CH_3_)_2_), 3.78 (s, 3H, –CH_3_), 4.36 (s, 2H, –CH_2_N­(CH_3_)_2_), 4.73 (br s, 2H, –NH_2_),6.63–6.68 (m, 2H, benzene proton), 7.79–7.81 (m, 1H, benzene proton) ppm.

#### Methyl 3-Amino-5-((dimethylamino)­methyl)­benzoate (**23b**)

Prepared starting from **22b** (0.03 mmol, 0.007 g). Mp: 148–149 °C (acetonitrile); yield: 77%. ^1^H NMR (400 MHz, CDCl_3_) δ: 2.26 (s, 6H, –N­(CH_3_)_2_), 3.77 (br s, 2H, –NH_2_), 3.49 (s, 2H, –CH_2_N­(CH_3_)_2_), 3.90 (s, 3H, –CH_3_), 6.88–6.89 (m, 1H, benzene proton), 7.26–7.29 (m, 1H, benzene proton), 7.35–7.37 (m, 1H, benzene proton) ppm.

#### 3-((Dimethylamino)­methyl)-N^1^-(quinolin-4-yl)­benzene-1,4-diamine (**29**)

Prepared starting from **28** (0.03 mmol, 0.01 g). Mp: 145–148 °C (acetonitrile); yield: 75%. ^1^H NMR (400 MHz, CD_3_OD) δ: 2.25 (s, 6H, –N­(CH_3_)_2_), 3.45 (s, 2H, –CH_2_N­(CH_3_)_2_),5.03 (br s, 2H, –NH_2_), 6.61 (s, 1H, benzene proton), 6.85 (d, 1H, *J* = 8.4 Hz, quinoline proton), 7.03 (s, 1H, benzene proton), 7.10 (d, 1H, *J* = 7.6 Hz, benzene proton), 7.49–7.54 (m, 1H, quinoline proton), 7.66–7.73 (m, 1H, quinoline proton), 7.86 (d, 1H, *J* = 7.6 Hz, quinoline proton), 8.23–8.31 (m, 2H, quinoline protons) ppm.

#### *N* ^4^-(4-Amino-3-((dimethylamino)­methyl)­phenyl)-6-methylpyrimidine-2,4-diamine (**32**)

Prepared starting from **31** (0.03 mmol, 0.01 g). Mp: 96–98 °C (toluene); yield: 70%. ^1^H NMR (400 MHz, CDCl_3_) δ: 2.07 (s, 3H, –CH_3_), 2.30 (s, 2H, –CH_2_N­(CH_3_)_2_), 3.43 (s, 2H, –CH_2_N­(CH_3_)_2_), 5.05 (br s, 4H, 2x -N*H*_2_), 5.70 (s, 1H, pyrimidine proton), 6.67 (br s, 1H, –NH−), 6.65 (d, d, 1H, *J* = 8.4 Hz, benzene proton), 6.88 (s, 1H, benzene proton), 6.98–7.00 (m, 1H, benzene proton) ppm.

#### *N* ^1^,*N* ^1^-Dimethylbenzene-1,3,5-Triamine (**42**)

Prepared starting from *N,N*-dimethyl-3,5-dinitroaniline (0.03 mmol, 0.006 g). mp: >250 °C (methanol); yield: 76%. ^1^H NMR (400 MHz, CDCl_3_) δ: 2.89 (s, 6H, 2x-CH_3_), 3.52 (br s, 4H, 2xNH_2_), 5.54 (s, 1H, benzene proton), 5.59 (s, 2H, benzene protons) ppm.

#### General Procedure for the Synthesis of the Acid Intermediate Compounds **25a,b**

A solution of the appropriate ester (1.14 mmol, 1 equiv) and 2N KOH (4.56 mmol, 0.26 g, 4 equiv) in ethanol (15 mL) was stirred overnight at rt. Then, the solvent was evaporated, and 2 N HCl was slowly added until the aqueous phase reached a pH value of 5.0. The solid was filtered, washed with water (2 × 10 mL), and recrystallized from methanol to obtain the pure carboxylic acid.

#### 2-((Dimethylamino)­methyl)-4-(quinolin-4-ylamino)­benzoic Acid (**25a**)

Prepared starting from **24a** (1.14 mmol, 0.38 g). Mp: >250 °C (methanol); yield: 88%. ^1^H NMR (400 MHz, DMSO-*d*_6_) δ: 2.66 (s, 6H, N­(CH_3_)_2_), 4.21 (s, 2H, –CH_2_N­(CH_3_)_2_), 7.16 (d, 1H, *J* = 5.6 Hz, benzene proton), 7.50–7.55 (m, 2H, benzene and quinoline protons), 7.63–69 (m, 1H, benzene proton), 7.83–7.89 (m, 1H, quinoline proton), 8.02 (d, 2H, *J* = 8.4 Hz, quinoline protons), 8.58 (d, 1H, *J* = 5.6 Hz, quinoline proton), 8.63 (d, 1H, *J* = 7.6 Hz, quinoline proton), 10.16 (br s, 1H, –NH−), 12.03 (br s, 1H, –COOH) ppm.

#### 3-((Dimethylamino)­methyl)-5-(quinolin-4-ylamino)­benzoic Acid (**25b**)

Prepared starting from **24b** (1.14 mmol, 0.38 g). Mp: >250 °C (methanol); yield: 83%. ^1^H NMR (400 MHz, DMSO-*d*_6_) δ: 2.63 (s, 6H, –N­(CH_3_)_2_), 4.25 (s, 2H, –CH_2_N­(CH_3_)_2_), 7.02 (br s, 1H, –NH−), 7.21–7.24 (m, 2H, benzene protons), 7.52–7.55 (m, 1H, quinoline protons), 7.68–7.72 (m, 1H, benzene proton), 7.93–7.95 (m, quinoline proton), 8.05 (d, 1H, *J* = 8.4 Hz, quinoline proton), 8.62 (d, 1H, *J* = 7.6 Hz, quinoline proton), 8.66 (d, 1H, *J* = 5.2 Hz, quinoline proton), 10.19 (br s, 1H, –NH–), 12.03 (br s, 1H, –COOH) ppm.

#### Synthesis of 3-((dimethylamino)­methyl)-4-nitroaniline (**27**)

5-Amino-*N*,*N*-dimethyl-2-nitrobenzamide (0.45 mmol, 0.09 g, 1 equiv) was dissolved in dry THF (20 mL), and 1 M borane tetrahydrofuran complex solution (2.72 mmol, 2.72 mL, 6 equiv) was added slowly at 0 °C and subsequently the reaction was heated to 70 °C. After 6 h, the reaction was complete, and water (20 mL) was added at rt. The mixture was extracted with ethyl acetate (3 × 30 mL) and washed with brine (3 × 30 mL), and the combined organic extracts were dried over sodium sulfate. Upon evaporation of the solvent, the crude product was purified by column chromatography (SiO_2_ eluting with ethyl acetate/methanol 5:1), giving the pure compound **27**. Mp: 214–216 °C (acetonitrile/methanol); yield: 78%. ^1^H NMR (400 MHz, CD_3_OD) δ: 2.29 (s, 6H, –N­(CH_3_)_2_), 3.78 (s, 2H, –CH_2_N­(CH_3_)_2_), 6.58 (d, 1H, *J* = 8.8 Hz, benzene proton), 6.70 (s, 1H, benzene proton), 7.97 (d, 1H, *J* = 8.8 Hz, benzene proton) ppm.

#### Synthesis of Di*tert*-butyl (5-(Bromomethyl)-1,3-phenylene)­dicarbamate (**34**)

Di-*tert*-butyl (5-(hydroxymethyl)-1,3-phenylene)­dicarbamate (2.96 mmol, 1.00 g, 1 equiv) was dissolved in dry THF (10 mL) under a nitrogen atmosphere, and tetrabromomethane (7.5 mmol, 2.49 g, 2.5 equiv) was added slowly. Next, a solution of triphenylphosphine (8.9 mmol, 2.33 g, 3 equiv) in anhydrous THF (5 mL) was added dropwise to the reaction mixture at 0 °C. The reaction was complete after leaving the mixture overnight at rt. The residue obtained upon solvent evaporation was purified by column chromatography (SiO_2_ eluting with ethyl acetate/hexane:1/3), providing the pure **34**. Mp: 180–182 °C (acetonitrile/methanol); yield: 69%; ^1^H NMR (400 MHz, CDCl_3_) δ: 1.53 (s, 18H, 2x C­(CH_3_)_3_), 4.61 (s, 2H, –CH_2_Br), 6.53 (br s, 2H, –OCONH−), 7.16 (s, 2H, benzene protons), 7.38 (s, 1H, benzene proton) ppm.

#### General Procedure for the Synthesis of the Amine Intermediate Compounds **36a-c, 45, 47**

A solution of 4 N HCl in dioxane (0.2 mL, 30 equiv) was slowly added at 0 °C to a cooled solution of the appropriate di-*tert*-butyl (1,3-phenylene)­dicarbamate **35a**–**c** (0.03 mmol, 1 equiv) in dry THF (3 mL). The reaction was allowed to warm to room temperature and stirred for 10 h; then the mixture was quenched with diethyl ether, and the resulting solid was filtered off. The obtained crude product was purified by column chromatography (SiO_2_ eluting with ethyl acetate/methanol:1/1) to afford the pure free amine product (**36a**, **36b**, or **36c**). For **35a** only, the reaction was not stirred upon completion (10 h), but it was interrupted before to obtain the mono-*tert*-butyl carbamate derivative **45**. When the TLC carried out on the mixture showed the formation of the monocarbamate, the reaction was quenched at room temperature by 2N sodium bicarbonate solution (20 mL), and the mixture was extracted with DCM (3 × 30 mL) and washed with brine (3 × 30 mL). The combined organic layers were then dried with anhydrous sodium sulfate, filtered, and concentrated under reduced pressure to give a crude product, which was purified by column chromatography (SiO_2_ eluting with ethyl acetate/methanol 1/1) to afford the pure **45**.

#### 5-((Dimethylamino)­methyl)­benzene-1,3-diamine (**36a**)

Prepared starting from **35a** (0.03 mmol, 0.01 g). Mp: 230–232 °C (methanol); yield: 82%. ^1^H NMR (400 MHz, DMSO-*d*_6_) δ: 2.67 (s, 6H, –N­(CH_3_)_2_), 4.13 (s, 2H, –CH_2_N­(CH_3_)_2_), 3.57 (br s, 4H, 2x –NH_2_), 6.66 (s, 1H, benzene proton), 6.70 (s, 2H, benzene protons), 10.54 (br s, 1H, –N­(CH_3_)_2_ hydrochloride proton) ppm.

#### 5-((4-Methylpiperazin-1-yl)­methyl)­benzene-1,3-diamine (**36b**)

Prepared starting from **35b** (0.03 mmol, 0.01 g). Mp: >250 °C (methanol); yield: 85%. ^1^H NMR (400 MHz, CDCl_3_) δ: 2.21 (s, 3H, –CH_3_), 2.38 (s, 8H, piperazine protons), 3.42 (s, 2H, –CH_2_−), 3.57 (br s, 4H, 2x –NH_2_), 5.86 (s, 1H, benzene proton), 6.04 (s, 2H, benzene protons) ppm.

#### 5-((1H-Imidazole-1-yl)­methyl)­benzene-1,3-diamine (**36c**)

Prepared starting from **35c** (0.03 mmol, 0.01 g). Mp: >250 °C (methanol); yield: 80%. ^1^H NMR (400 MHz, DMSO-*d*_6_) δ: 4.63 (br s, 4H, 2x –NH_2_), 5.38 (s, 2H, –CH_2_−), 6.55 (s, 2H, benzene protons), 6.66 (s, 1H, benzene proton), 7.74–7,77 (m, 2H, imidazole protons), 9.34 (s, 1H, imidazole proton) ppm.

#### *Tert*-butyl (3-amino-5-((dimethylamino)­methyl)­phenyl)­carbamate (**45**)

Prepared starting from **35a** (0.03 mmol, 0.01 g) before completion of the reaction. Mp: 235–240 °C (methanol); yield: 50%; ^1^H NMR (400 MHz, CDCl_3_) δ: 1.51 (s, 9H, –C­(CH_3_)_3_), 2.35 (s, 6H, –N­(CH_3_)_2_), 3.44 (s, 2H, –CH_2_N­(CH_3_)_2_), 3.99 (br s, 2H, –NH_2_), 6.40 (s, 1H, benzene proton), 6.60–6.62 (s and bs, 2H, −OCONH- and benzene proton), 6.88 (s, 1H, benzene proton) ppm.

#### 5-((Dimethylamino)­methyl)-N^1^-(3-(quinolin-4-ylamino)­benzyl)­benzene-1,3-diamine (**47**)

Prepared starting from **46** (0.3 mmol, 0.16 g) Mp: >250 °C (methanol); yield: 95%. ^1^H NMR (400 MHz, DMSO-*d*_6_) δ: 2.15 (s, 6H, N­(CH_3_)_2_), 3.18 (s, 2H, –CH_2_N­(CH_3_)_2_), 4.18 (d, 2H, *J* = 5.2 Hz, CH_2_), 5.04 (br s, 2H, –NH_2_), 6.24 (s, 1H, benzene proton), 6.39 (s, 1H, benzene proton), 6.44 (s, 1H, benzene proton), 6.73 (s, 1H, benzene proton), 6.92–6.94 (d, 2H, benzene and quinoline proton), 7.14 (d, 1H, *J* = 6.8 Hz, benzene proton), 7.20 (d, 1H, *J* = 8.0 Hz, benzene proton), 7.35–7.38 (m, 2H, benzene proton and NH), 7.52 (t,1H, *J* = 7.6 Hz, quinoline proton), 7.68 (t, 1H, *J* = 8.0 Hz, quinoline proton), 7.85 (d, 1H, *J* = 8.4 Hz, quinoline proton), 8.34–8.38 (m, 2H, quinoline protons), 8.95 (br s, 1H, -NH-) ppm.

#### Synthesis of *tert*-butyl (3-((dimethylamino)­methyl)-5-((3-(quinolin-4-ylamino)­benzyl)­amino)­phenyl)­carbamate (**46**)

The 3-(quinolin-4-ylamino)­benzaldehyde **44** ^22^ (0.81 mmol, 0.20 g) and the mono-*tert*-butyl carbamate **45** (0.80 mmol, 0.21 g) were stirred in anhydrous DCE (5 mL) for 5 min. Afterward, sodium triacetoxyborohydride (1.05 mmol, 0.29 g) was added, and the resulting mixture was stirred overnight at 60 °C. The reaction was quenched with 10 mL of water and extracted with DCM (3 × 20 mL). The organic layer was washed with saturated sodium chloride (20 mL) and dried with sodium sulfate. Upon evaporation of the solvent, the crude product was purified by column chromatography (SiO_2_ eluting with ethyl acetate/methanol 5:1), giving the pure compound **46**. Mp: >250 °C (methanol); yield: 50%. ^1^H NMR (400 MHz, CDCl_3_) δ: 1.40 (s, 9H, C­(CH_3_)_3_), 2.13 (s, 6H, –N­(CH_3_)_2_), 3.21 (s, 2H, –CH_2_N­(CH_3_)_2_), 4.04 (br s, 1H, –CH_2_NH−), 4.28 (d, 2H, –CH_2_NH−), 6.24 (s, 1H, benzene proton), 6.39 (s, 1H, benzene proton), 6.44 (s, 1H, benzene proton), 6.73 (s, 2H, benzene protons), 7.06 (d 1H, *J* = 7.6 Hz, quinoline proton), 7.13 (d 1H, *J* = 8.0 Hz, benzene proton), 7.29 (t, 1H, *J* = 7.6 Hz, benzene proton), 7.41–7.45 (m, 1H, quinoline proton), 7.59–7.63 (m, 1H, quinoline proton), 7.89 (d, 1H, *J* = 8.4 Hz, quinoline proton), 7.97 (d, 1H, *J* = 8.4 Hz, quinoline proton), 8.47 (d, 1H, *J* = 7.2 Hz, quinoline proton) ppm.

#### Synthesis of benzyl (3-amino-5-((dimethylamino)­methyl)­phenyl)­carbamate (**48**)

Benzyl chloroformate (1.09 mmol, 0.16 mL, 1 equiv) was added to a solution of 5- ((dimethylamino)­methyl)­benzene-1,3-diamine (**36a**) (1.09 mmol, 0.3 g, 1 equiv) and triethylamine (8.19 mmol, 1.14 mL, 7.5 equiv) in dry THF (7 mL). The resulting mixture was stirred overnight at rt; then, the reaction was quenched with NaHCO_3_ (20 mL) and extracted with DCM (3 × 30 mL). The combined organic extracts were dried, and the residue obtained upon solvent evaporation was purified by column chromatography (SiO_2_ eluting with ethyl acetate/methanol:1.5/2) to provide pure **48**. Mp: >250 °C (methanol); yield: 52%. ^1^H NMR (400 MHz, CDCl_3_) δ: 2.22 (s, 6H, N­(CH_3_)_2_), 3.30 (s, 2H, –CH_2_N­(CH_3_)_2_), 3.61 (br s, 2H, –NH_2_), 5.11 (s, 2H, –CH_2_−), 6.34 (s, 1H, benzene proton), 6.50 (s, 1H, benzene proton), 6.60 (s, 1H, benzene proton), 6.82 (s, 1H, –OCONH−), 7.26–7.32 (m, 5H, benzene protons) ppm.

## Biochemistry

### DNMT Inhibition Assay

Full-length histidine-tagged human DNMT1 (182 kDa) was produced and purified according to^37^ and catalytic human DNMT3A/DNMT3L as described in.^38^ Compound activities were determined with a fluorescence-based assay.^30^ In brief, a double-stranded DNA, comprising a unique CpG site overlaying an endonuclease restriction site for a methylation-sensitive enzyme, was used. This oligonucleotide comprises a 6-carboxyfluorescein (6-FAM) at one end and biotin on the other end, allowing immobilization into a 384-well plate (PerkinElmer) precoated with avidin. Compounds to be evaluated and SAM as methyl donor were added, followed by DNMT3*A*/3L to start the methylation reaction, which was prolonged 1 h at 37 °C. After several washes, with PBS tween (0.05%) containing NaCl (0.5 M) and PBS tween (0.05%). The restriction step was performed with HpyCH4IV (New England, BioLabs) to hand on only the specific fluorescence signal. Fluorescence was quantified on an EnVision 2103 Multilabel Reader (PerkinElmer). Inhibition percentages are defined as 100­(100-(X_meth_-X_restri_)/(X_DNA_-X_restri_), where X_meth_, X_restri_, and X_DNA_ are, respectively, the fluorescence signals of the compound methylation, restriction, and DNA controls. These percentages for a concentration range were then used to calculate the half maximal effective concentration (EC_50_) (average of at least 2 experiments) for the compound of interest by using the nonlinear regression with sigmoidal dose–response (variable slope) tool of GraphPad Prism software.

For DNMT1, the same assay was optimized using a hemimethylated duplex at 1 CpG site (FAM −5′ GCA TAT ATA TGA CGA TmCG TGT AGG TCA CTA CCA GAC ATG CAC TG 3′/5′ Biot- CAG TGC ATG TCT GGT AGT GAC CTA CAC GAT CGT CAT ATA TAT GC 3′). The methylation reaction is performed by adding human DNMT1 at a final concentration of 200 nM in a total volume of 30 μL in the presence of the chemical compound to be tested at the desired concentration and the cofactor SAM at the final concentration of 20 μM. After incubation (1 h 37 °C) the plate is washed, and the methylation sensitive restriction enzyme Bsh1285I (Thermo Fisher Scientific) is added. After 1.30 h incubation at 60 °C the plate is washed and the fluorescence measured with a PerkinElmer Envision Multilabel Plate Reader. The data are expressed as percentage of inhibition vs log concentration (M). Data are normalized referring to the “restriction control” (wells coated with labeled duplex not treated nor exposed to DNMT1, but only cleaved by the restriction enzyme) as the maximum of inhibition (100%) and to the “DMSO control” (wells coated with labeled duplex but treated just with DMSO 0.1%, exposed to DNMT1 and then to Bsh1285I) as the minimum of inhibition (0%) (total methylation).

### G9a and PRMT1 Inhibition Assays

The appropriate methyltransferase substrate (2.5 μM histone H3 (aa 1–21) peptide for G9a, and 5 μM histone H4 for PRMT1) was added in freshly prepared reaction buffer (50 mM Tris–HCl (pH 8.5), 5 mM MgCl_2_, 50 mM NaCl, 0.01% Brij35, 1 mM DTT, 1% DMSO). Compound **14** was tested in single-dose mode, in duplicate, at 50 and 100 μM. Control compound, SAH (*S*-(5′-adenosyl)-l-homocysteine), was tested in 10-dose IC_50_ mode with 3-fold serial dilution starting at 100 μM. The methyltransferase enzyme was delivered into the substrate solution, and the mixture was mixed gently. Afterward, the tested compounds dissolved in DMSO were delivered into the enzyme/substrate reaction mixture by using Acoustic Technology (Echo 550, LabCyte Inc., Sunnyvale, CA, USA) in the nanolitre range, and 1 μM ^3^H-SAM was also added into the reaction mixture to initiate the reaction. The reaction mixture was incubated for 1 h at 30 °C and then it was delivered to filter-paper for detection. The data were analyzed using Excel and GraphPad Prism software for IC_50_ curve fits.

### Thermal Denaturation Assay

DNA thermal denaturation experiments were conducted as described in.^28^ Hairpin DNA duplex (5′-GCCTTCGGTGGCTTTTGCCACCGAAGGC-3′) was used at 2 μM in the absence or in the presence of the inhibitor at 10 μM in the Tm assay buffer (100 mM NaCl, 10 mM lithium cacodylate pH 7.2). The temperature at which 50% of the duplex is denatured Tm was calculated as previously described.^28^

## Biology

### Cell Lines and Culture Conditions

U937 histiocytic lymphoma, H460 and A549 lung adenocarcinoma, MDA-MB-231 breast carcinoma, and HeLa epithelial cervix carcinoma cell lines were maintained in RPMI 1640 medium (Euroclone, Milan, Italy) contained 10% fetal bovine serum (FBS; Euroclone), 2 mM l-glutamine (Euroclone), and antibiotics (100 U/mL penicillin, 100 g/mL streptomycin, Euroclone) in a humidified atmosphere with 5% CO_2_ at 37 °C. HCT-116 colon carcinoma cells were propagated in Dulbecco’s modified Eagle medium (DMEM) (Euroclone) with 10% FBS, 2 mM l-glutamine, and antibiotics. BJ and HCT-116 P53^–/–^ cells were kindly provided by Dr. Erica Salvati, CNR IBPM. The human hTERT RPE-1 - epithelial cell line immortalized with hTERT—(kind gift of Dr Giulia Guarguaglini, CNR IBPM) was grown in complete DMEM/F12 (Dulbecco’s Modified Eagle Medium F-12). All cell lines were routinely tested for mycoplasma contamination. The experiments were performed with early (3–20) cell passages. Cell lines were treated with compounds at the indicated concentrations in the exponential growth phase.

### Combined Bisulfite Restriction Analysis Methylation Assay

This technique combines bisulfite conversion-based polymerase chain reaction with restriction digestion. DNA extraction and bisulfite treatment DNA was isolated from cultured cells treated or not with the compounds as indicated using the DNeasy Blood and Tissue Kit according to the manufacturer’s specifications (Qiagen). DNA bisulfite conversion was performed on 200 ng of DNA using the EZ DNA Methylation-Gold Kit according to the manufacturer’s specifications (Zymo Research) and bisulfited DNA was eluted with water.

### CDKN2A Promoter and LINE-1 PCR Amplification of Bisulfite-Treated DNA

DNA amplification was set up with 100 ng eluted bisulfited DNA in 50 μL PCR reaction containing 1 × PCR buffer, 2.5 mM MgCl_2_, 0.3 mM dNTPs, 400 nM each primer and 1.25 units EpiTaq HS (Takara) on C1000 Touch thermal cycler by 98 °C for 10 s, 55 °C for 30 s, 72 °C for 30 s × 40 cycles. Twenty μL of PCR amplicons were digested in 30 μL by 2 units of BsiEI (for CDKN2A promoter) or HinfI (for LINE-1) at 60 or 37 °C, respectively, for 90 min. DNA fragments were migrated by 2% agarose gel electrophoresis and each band was quantified by Image Lab Software v2.0 (BioRad Laboratories). The percentage of demethylation is calculated as demethylation (%) = 100­[NT-T/NT] where NT = DMSO-treated cells and T = treated cells. Primers used were: for CDKN2A promoter: forward, 5′-GGTTTTTTTAGAGGATTTGAGGGATAGG-3′ reverse, 5′-CTACCTAATTCCAATTCCCCTACAAACTTC-3′ for LINE-1: forward, 5′-TTGAGTTGTGGTGGGTTTTATTTAG-3′ reverse, 5′-TCATCTCACTAAAAAATACCAAACA-3′

### Analysis of Cell Proliferation

Cell lines were treated with compounds at the indicated concentrations in the exponential growth phase. Briefly, increasing concentrations of different compounds (ranging from 0.05 to 100 μM) were added, and cell proliferation was evaluated by an MTT assay after 48 h. Control (0 μM) consists of 0.5% DMSO-treated cells. After treatments, MTT solution was added for 5 h at 0.5 mg/mL, the obtained crystals were dissolved in DMSO (Sigma-Aldrich, Milan, Italy), and the absorbance was read at a wavelength of 570 nm with a reader. All the experiments were performed in triplicate and repeated at least three times. IC_50_ values of compounds against cellular viability were determined by using nonlinear regression fitting curves with GraphPad Prism 8.

### Analysis of Cell Cycle and Apoptosis

For cell cycle analysis, after treatment, both floating and adherent cells were collected and fixed with cold 70% ethanol. Fixed cells were then resuspended in an RNase A (0.3 mg/mL) and propidium iodide (PI) solution (0.025 mg/mL). 10,000 events were then analyzed with a BD FACSCalibur Flow Cytometer: 4-Color (Becton Dickinson & Co., San Jose, CA, USA). The percentage of apoptosis was measured by analyzing sub-G1 peaks. Apoptotic cell death was also evaluated by using annexin V–fluorescein isothiocyanate (FITC) apoptosis kit (BD Bioscience) and assayed according to the manufacturer’s instructions.

### Live Cell Time-Lapse Microscopy

A total of 3 × 10^4^ cells were seeded per well of a 4-well μ-slide. Following a 24 h incubation period, the cells were exposed to varying concentrations of compounds. Cells were recorded under an inverted microscope. During observations, cells were kept at 37 °C and 5% CO_2_. Images were acquired using 40× objective over 5 days at 7 min intervals. The recorded cells were analyzed over time for cellular processes including entry into mitosis and cell death phenotypes. Processing was conducted using NIS-Elements AR 4.0.

### Analysis of DNMT1 and DNMT3A Protein Levels

For experiments on DNMT1 and DNMT3A protein levels, HCT-116 5 × 10^5^ cells were plated in 60 mm plate and treated with **14** in comparison with MS9024.^36^ Compounds were used at 0.1, 0.5, and 1 μM for 24 h. After treatment cells were collected and lysed for Western Blot Analysis.

### Western Blot Analysis

After each treatment, floating and adherent cells were collected and lysed in RIPA lysis buffer (pH 8) containing a protease and phosphatase inhibitor cocktail. Between 30 and 50 μg of total protein were resolved under reducing conditions, extracted, fractionated by SDS-PAGE in a 10–13.5% gel, transferred, blocked, and subjected to an immunoblot assay. The following antibodies were used: P53 (Cell Signaling #48818, 1:1000); Phospho-γH2AX (Cell Signaling Ser139,#2577, 1:1000); Cleaved Caspase-3 Antibody (Cell Signaling #9661, 1:1000); PARP (Sigma-Aldrich AB3565, 1:200); Vinculin (Santa Cruz Biotechnologies, sc-73614, 1:1000)); α-DNMT1 (Novus Biologicals, Littleton, CO, USA 1:1000); α-DNMT3A (SantaCruz Biotechnologies, CA, USA 1:200); α-Tubulin (Santa Cruz Biotechnologies, sc-5286, 1:1000); GAPDH (Santa Cruz Biotechnologies sc-32233, 1:1000). Blots are representative of at least two independent experiments. A densitometric evaluation was performed using ImageJ software and values normalized to relative controls, depending on the analysis.

## Supplementary Material





## Notes

Elemental analysis of compounds **4–21** (Table S1); HPLC traces for compounds **7**, **9**, **10**, **12**–**14** (Figure S1–6); Effect of **14** on lysine methyltransferase G9a and arginine methyltransferase PRMT1 (Table S2); Expression levels of DNMT1 and DNMT3A in cancer cell lines (Table S3); Cytotoxic effect of **17** in HCT-116 cells (Figure S7); Cell viability curves relative to noncancerous RPE and BJ cells treated with compound **14** (Figure S8); FACS analysis of the effect of **17** in HCT-116 cells (Figure S9); Effects of **14** on DNMT1 and DNMT3A protein levels (Figure S10) (PDF)
Molecular formula strings (CSV)

## Abbreviations

5-azadC: decitabine; CBr4, tetrabromomethane
COBRA: Combined Bisulfite Restriction Analysis
DNMT: DNA methyltransferase
DNMT3Acat: human catalytic DNMT3*A*/3L
DNMTi: DNMT inhibitors
Et_3_N: triethylamine
EWS: Ewing sarcoma
FACS: fluorescence-activated cell sorting
6-FAM: 6-carboxyfluorescein
GAPDH: glyceraldehyde-3-phosphate dehydrogenase
γH2AX: phosphorylated form of the histone H2AX
LINE-1: Long Interspersed Nucleotide Element 1
MTs: methyltranferases
Me_4_Si: tetramethylsilane
MTT: 3-(4,5-dimethylthiazol-2-yl)-2,5-diphenyltetrazolium bromide
OS: osteosarcoma
PARP: poly-ADP ribose polymerase
Ph_3_P: triphenylphosphine
PyBOP: benzotriazol-1-yloxytripyrrolidinophosphonium hexafluorophosphate
RPE: retinal pigment epithelium
SAH: *S*-adenosyl-l-homocysteine
SAM: *S*-adenosyl- l-methionine.
