# Supplementary material for: Quinoline-Based DNA Methyltransferase Inhibitors Featuring Basic Side Chains: Design, Synthesis, and Insight in Biochemical and Anticancer Cell Properties
Source: J Med Chem. 2025 Nov 11;68(22):23886–909. doi: 10.1021/acs.jmedchem.5c01029 (PMC12670401; doi:10.1021/acs.jmedchem.5c01029)

Quinoline-Based DNA Methyltransferase Inhibitors Featuring Basic Side Chains: Design, Synthesis, and Insight in Biochemical and Anticancer Cell Properties

Clemens Zwergel,[†] Chiara Lambona,[†] Rossella Fioravanti,[†] Alessia Raucci,[†] Francesco Fiorentino,[†] Guilaine Nchugoua Tchieh,[#] Corinne Jallet,[#] Laurent Lacroix,[ɸ] Michela Pierrettori,[^] Francesca Romana Pellegrini,[^] Yan Xiong,[°] Jian Jin,[°] Paola Arimondo,[#] Daniela Trisciuoglio,[^] Antonello Mai,[†,*] and Sergio Valente[†,*]

[†]*Department of Drug Chemistry and Technologies, Sapienza University of Rome, Piazzale Aldo Moro 5, 00185 Rome, Italy*
[#]*Institut Pasteur, Université Paris Cité, CNRS UMR n°3523 Chem4Life, Epigenetic Chemical Biology, Department of Structural Biology and Chemistry, F-75015, Paris, France*
[ɸ]*IBENS, Département de biologie, École Normale Supérieure, Université PSL, CNRS, INSERM, 75005, Paris, France*
[^]*IBPM Institute of Molecular Biology and Pathology, CNR National Research Council of Italy, Via degli Apuli 4, Rome, 00185, Italy*
°*Mount Sinai Center for Therapeutics Discovery, Departments of Pharmacological Science, Oncological Science and Neuroscience, Tisch Cancer Institute, Icahn School of Medicine at Mount Sinai, New York, New York 10029, United States*

*Antonello Mai**:** antonello.mai@uniroma1.it
*Sergio Valente: sergio.valente@uniroma1.it

Supporting Information

Content:

Table S1. Elemental Analysis of Compounds **4-21**. p. S3

Figure S1. HPLC traces for **7** (lab code: MC3716). p. S4

Figure S2. HPLC traces for **9** (lab code: MC3807). p. S5

Figure S3. HPLC traces for **10** (lab code: MC3563). p. S6

Figure S4. HPLC traces for **12** (lab code: MC3669). p. S7

Figure S5. HPLC traces for **13** (lab code: MC3821). p. S8

Figure S6. HPLC traces for **14** (lab code: MC3817). p. S9

Table S2. Effect of **14** on lysine methyltransferase G9a and arginine methyltransferase PRMT1.

p. S10

Table S3. Expression levels of DNMT1 and DNMT3A in cancer cell lines (The Human Protein Atlas). p. S11

Figure S7. Effect of **17** in HCT-116 cells treated with 0.01-50 μM compound for 48 h. p. S12

Figure S8. Cell viability curves relative to non-cancerous RPE and BJ cells treated with 0.01-25 μM compound **14**. p. S13

Figure S9. FACS analysis of the effect of **17** in HCT-116 cells at 2 μM for 48 h. p. S14

Figure S10. Effects of **14** on DNMT1 and DNMT3A protein levels. p. S15

Table S1. Elemental Analysis of Compounds **4-21**.

cmpd	MW	Elemental Analyses							
		calculated				found			
		C%	H%	N%	Cl%	C%	H%	N%	Cl%
**4**	628.00	57.38	5.30	17.84	16.93	57.45	5.42	17.69	16.84
**5**	628.00	57.38	5.30	17.84	16.93	57.49	5.44	17.62	16.77
**6**	628.00	57.38	5.30	17.84	16.93	57.22	5.21	17.97	17.04
**7**	628.00	57.38	5.30	17.84	16.93	57.50	5.44	17.62	16.74
**8**	628.00	57.38	5.30	17.84	16.93	57.21	5.19	17.99	17.01
**9**	577.51	60.31	5.24	19.40	12.28	60.17	5.09	19.54	12.35
**10**	628.00	57.38	5.30	17.84	16.93	57.52	5.41	17.69	16.77
**11**	541.10	66.59	6.15	20.71		66.22	6.01	20.89	
**12**	719.54	55.09	5.46	17.52	19.71	55.26	5.59	17.41	19.62
**13**	650.99	57.20	4.65	19.36	16.34	57.33	4.77	19.22	16.25
**14**	721.55	54.93	5.73	17.47	19.65	55.08	5.88	17.54	19.55
**15**	618.56	64.08	5.38	11.32	11.46	64.39	5.45	11.12	11.37
**16**	618.56	64.08	5.38	11.32	11.46	63.85	5.22	11.44	11.56
**17**	527.07	68.36	5.93	15.95	6.73	68.09	6.11	16.04	6.88
**18**	648.03	63.02	5.13	12.97	16.41	63.13	5.22	12.82	16.37
**19**	648.03	63.02	5.13	12.97	16.41	62.85	4.94	13.08	16.54
**20**	607.97	51.37	5.47	23.04	17.49	51.25	5.38	23.22	17.57
**21**	607.97	51.37	5.47	23.04	17.49	51.54	5.15	22.92	17.40

Figure S1. HPLC traces for **7** (lab code: MC3716).

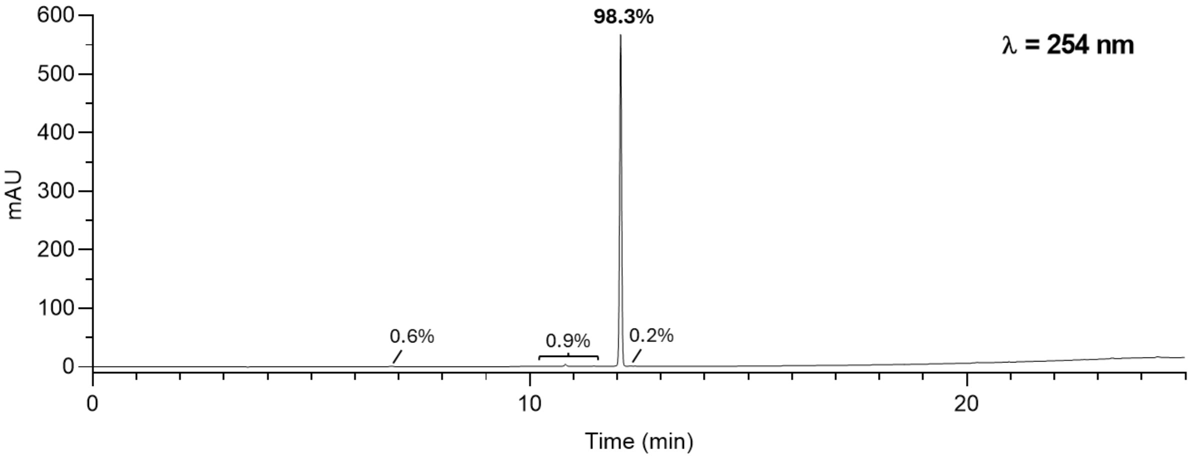

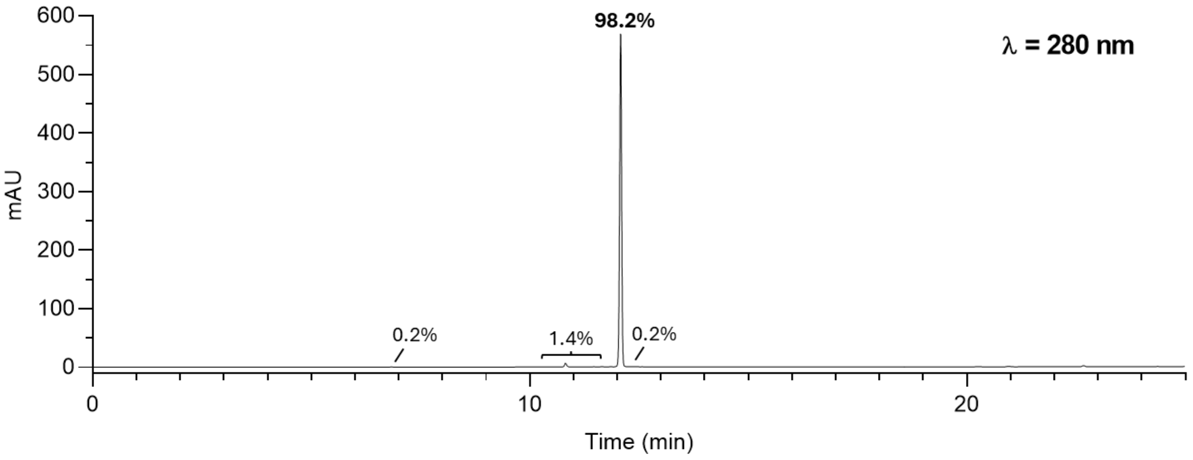

Figure S2. HPLC traces for **9** (lab code: MC3807).

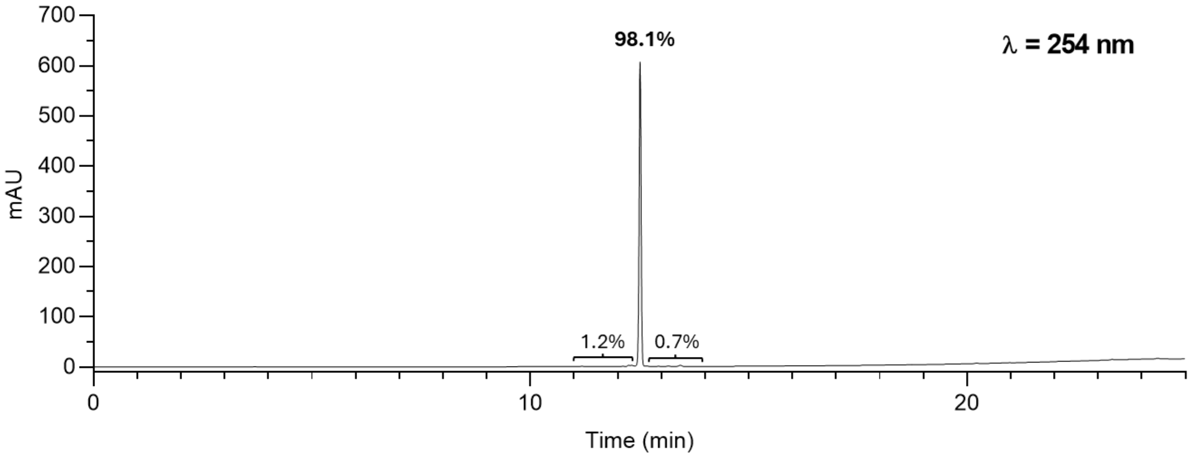

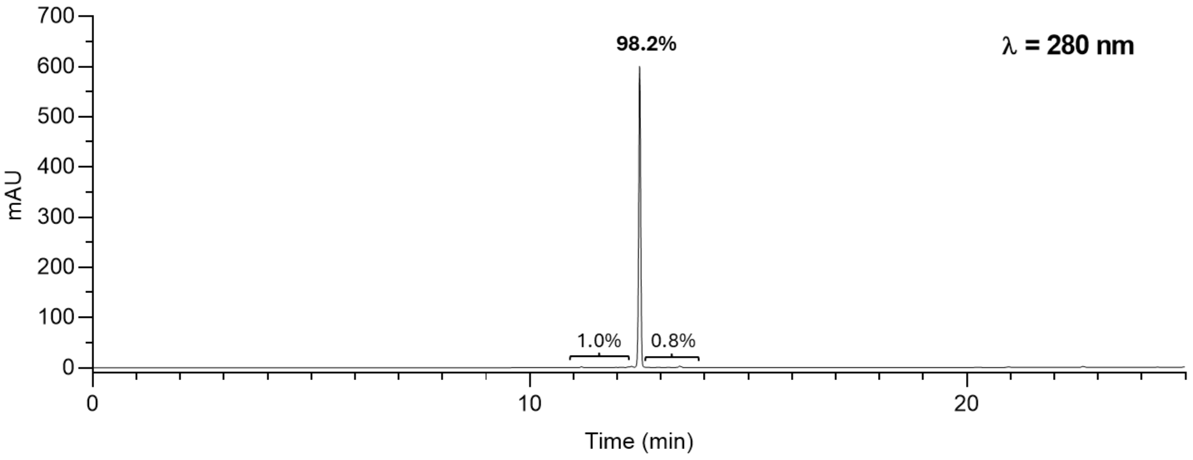

Figure S3. HPLC traces for **10** (lab code: MC3563).

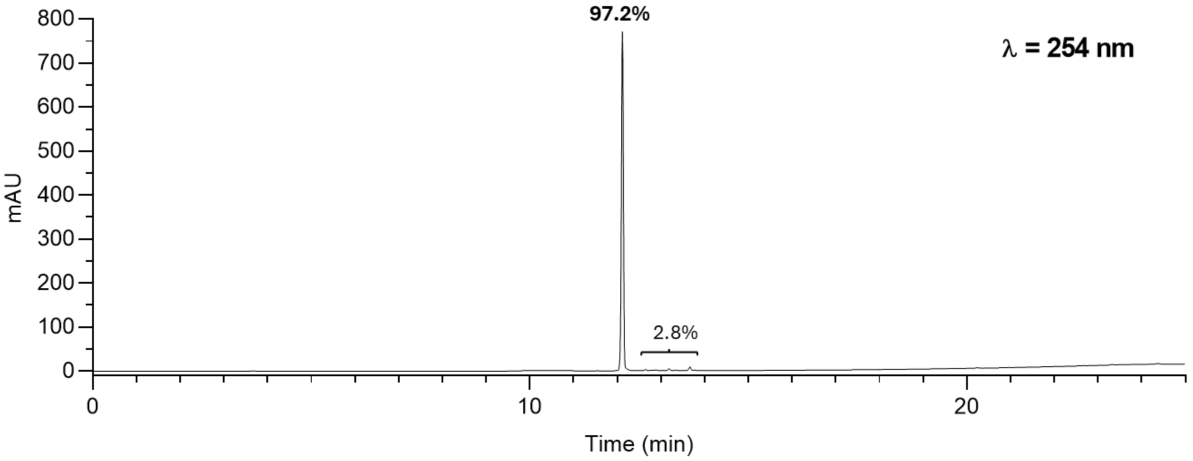

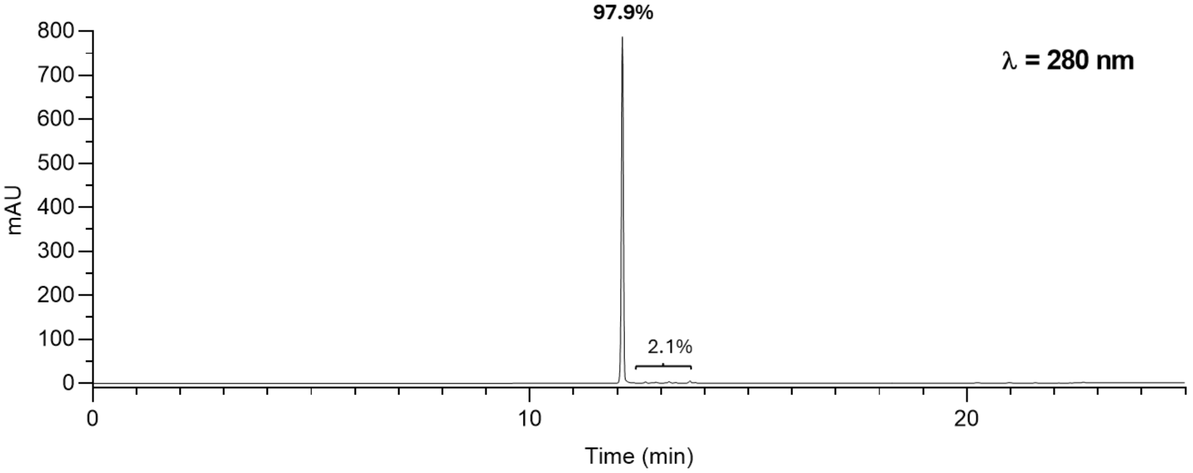

Figure S4. HPLC traces for **12** (lab code: MC3669).

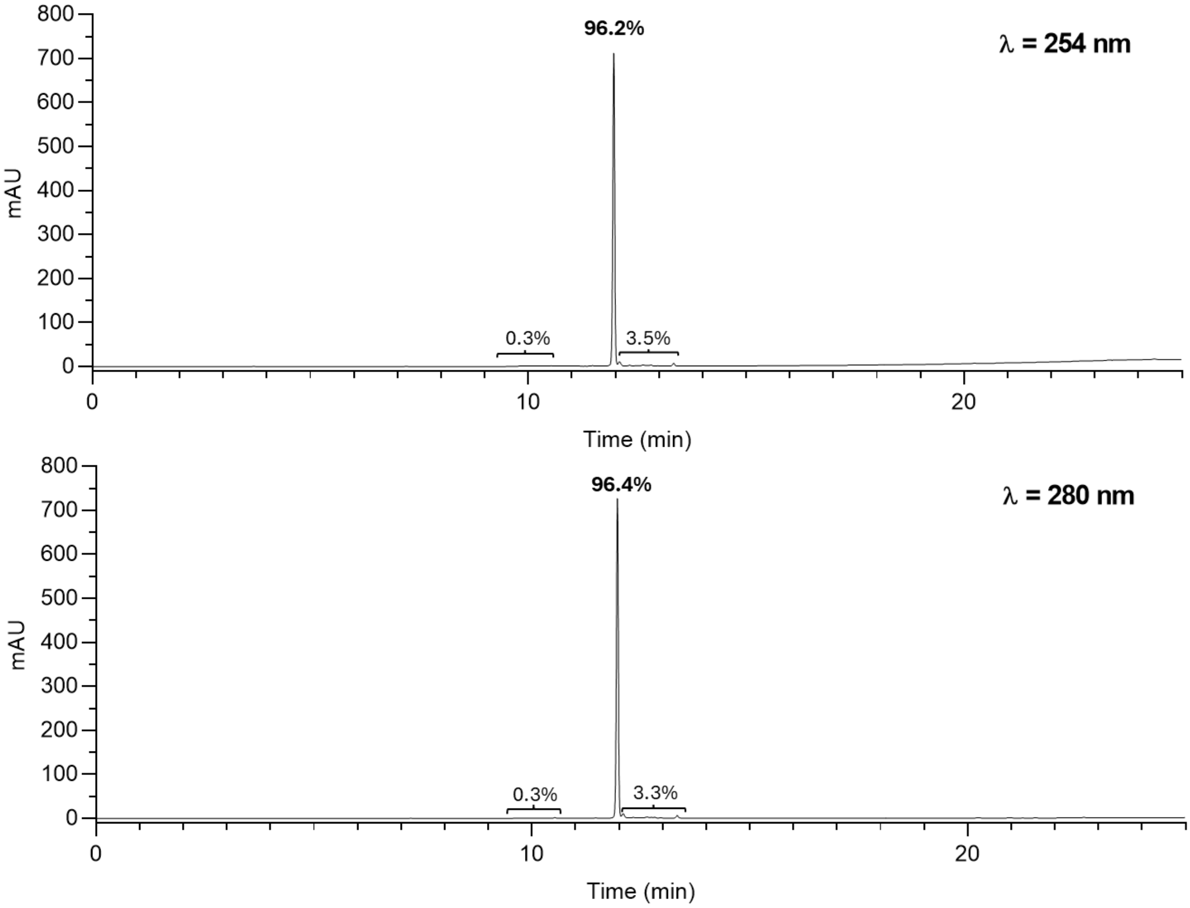

Figure S5. HPLC traces for **13** (lab code: MC3821).

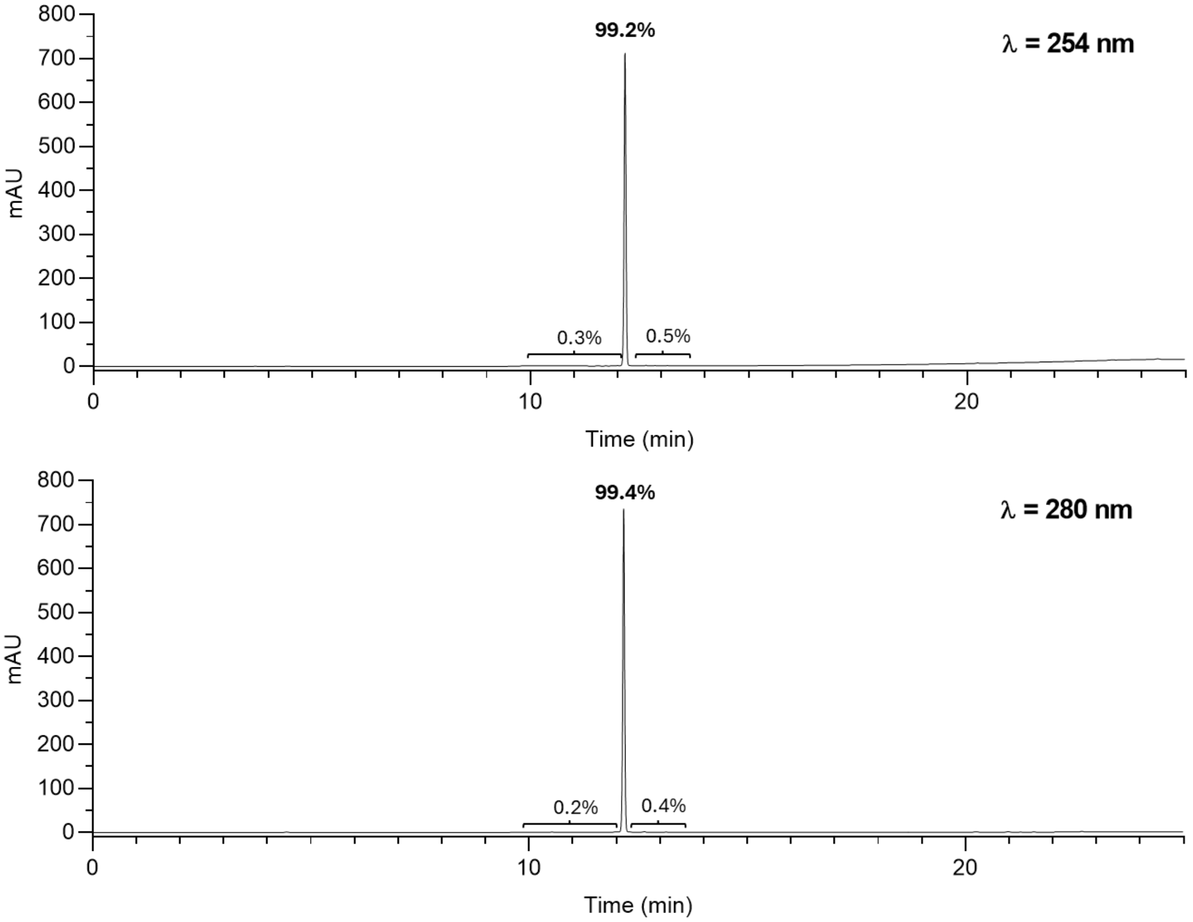

Figure S6. HPLC traces for **14** (lab code: MC3817).

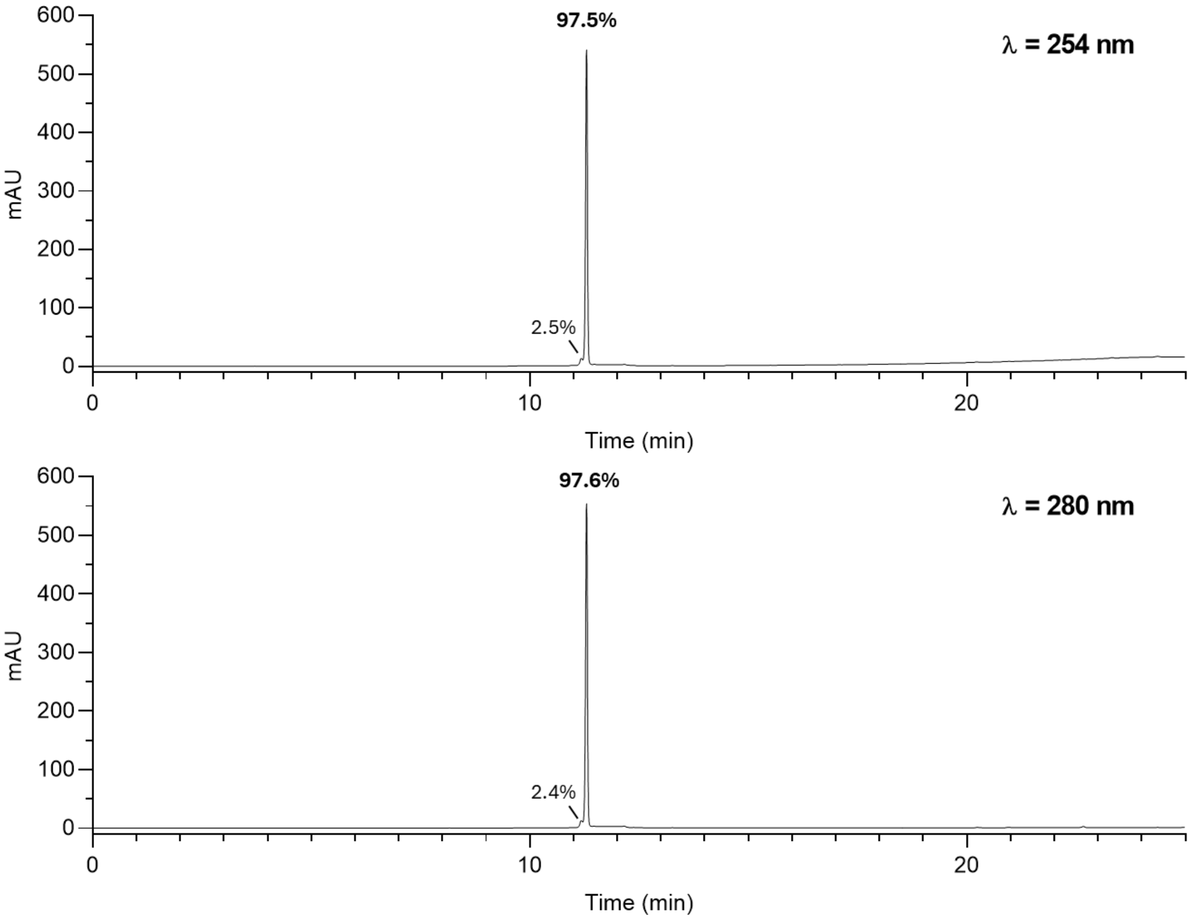

Table S2. Effect of **14** on lysine methyltransferase G9a and arginine methyltransferase PRMT1.

cpd	Enzyme % activity		IC_{50}, μM	
	G9a	PRMT1	G9a	PRMT1
**14**	69.7 (@50 μM)<br>30.1 (@100 μM)	107.3 (@50 μM)<br>105.9 (@100 μM)	>50	>100
SAH			3.53	0.93

Table S3. Expression levels of DNMT1 and DNMT3A in cancer cell lines (The Human Protein Atlas).

	Cancer cell line, nTPM					
	A549	H460	HCT-116	HeLa	MDA-MB-231	U937
DNMT1	42.6	ND[a]	91.2	44.7	89.2	78.4
DNMT3A	9.2	ND	4.2	16.1	3.9	8.0

[a]ND, not determined.

Figure S7. Effect of **17** in HCT-116 cells treated with 0.01-50 μM compound for 48 h.

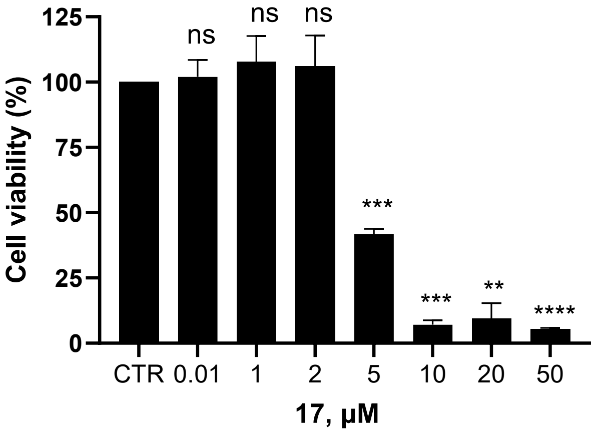

Figure S8. Cell viability curves relative to non-cancerous RPE (**A**) and BJ (**B**) cells treated with 0.01-25 µM compound **14**.

A

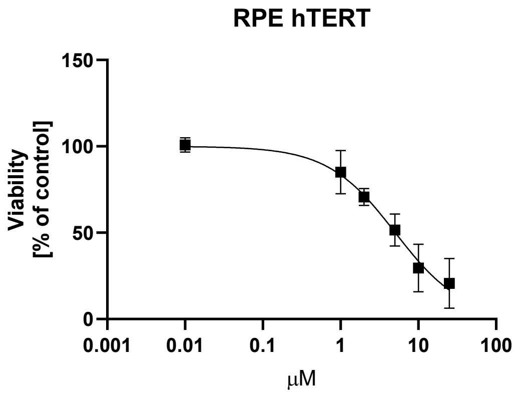

B

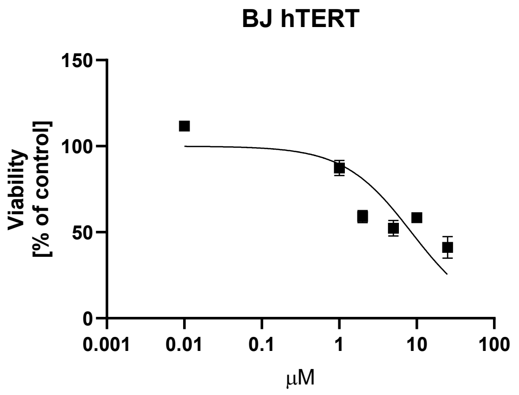

Figure S9. FACS analysis of the effect of **17** in HCT-116 cells at 2 μM for 48 h.

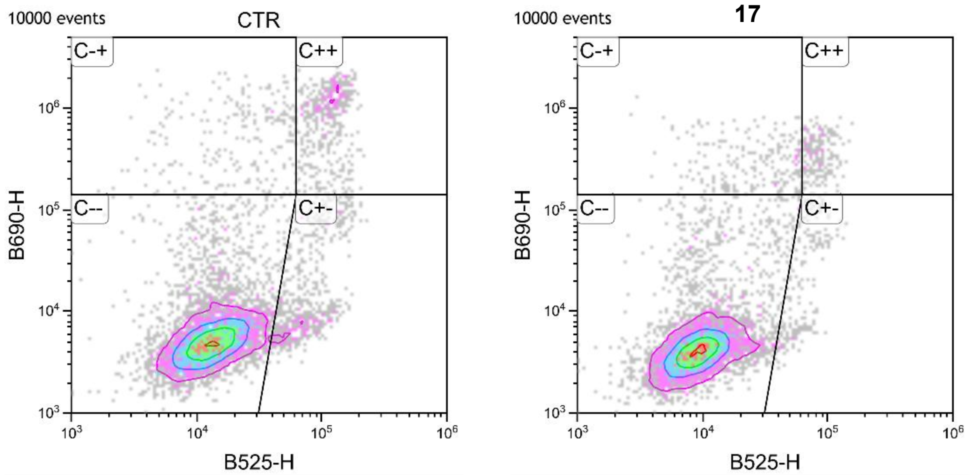

Gate	%Gated
All	100,00
C--	81,33
C-+	3,10
C+-	8,73
C++	6,85

Gate	%Gated
All	100,00
C--	90,74
C-+	3,19
C+-	2,95
C++	3,13

Figure S10. Effects of **14** on DNMT1 and DNMT3A protein levels.

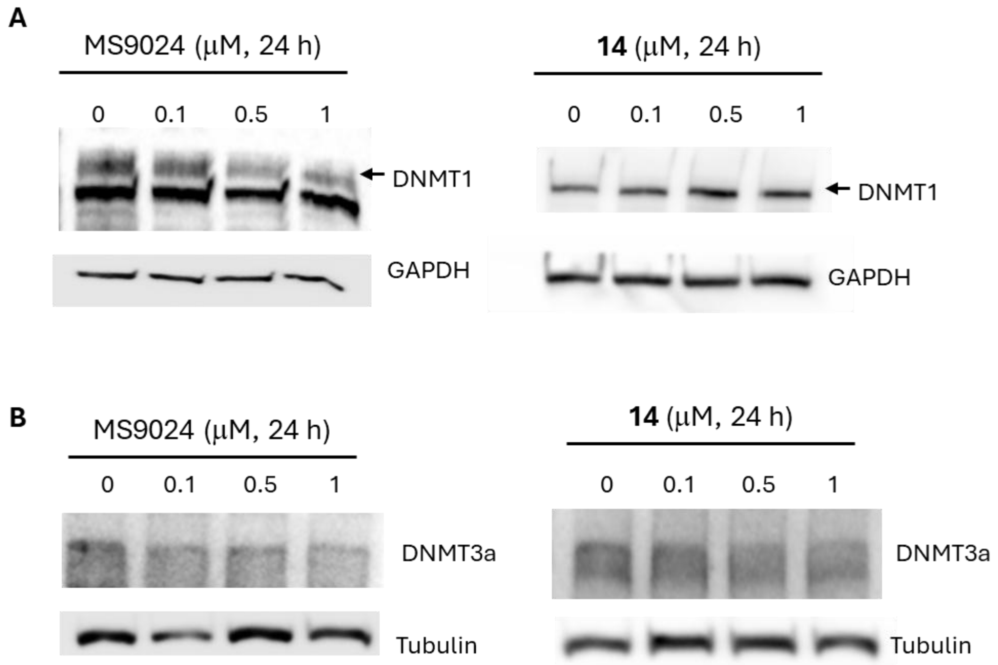

Supplement: Supplementary file 1 [file jm5c01029_si_001.pdf]
